# Exogenous Jasmonic Acid Alleviates Blast Resistance Reduction Caused by *LOX3* Knockout in Rice

**DOI:** 10.3390/biom13081197

**Published:** 2023-07-31

**Authors:** Shunyu Su, Ping Tang, Rubin Zuo, Hongfeng Chen, Tianqi Zhao, Shumin Yang, Jing Yang

**Affiliations:** 1 State Key Laboratory for Conservation and Utilization of Bio-Resources in Yunnan, Yunnan Agricultural University, Kunming 650201, China; 2021210313@stu.ynau.edu.cn (S.S.); 2001015@ynau.edu.cn (P.T.); 2022210344@stu.ynau.edu.cn (R.Z.); 2020210289@stu.ynau.edu.cn (H.C.); 2021210323@stu.ynau.edu.cn (T.Z.); 2019311106@stu.ynau.edu.cn (S.Y.); 2 Key Laboratory of Agro-Biodiversity and Pest Management of Ministry of Education, Yunnan Agricultural University, Kunming 650201, China

**Keywords:** rice, *Magnaporthe oryzae*, lipoxygenase 3, trade-off, rice quality and resistance

## Abstract

Lipoxygenase 3 (*LOX3*) is a lipid peroxidase found in rice embryos that is known to affect seed quality. Interestingly, deletion of the *LOX3* gene has been shown to improve rice seed quality but decrease resistance to rice blast disease and drought. To investigate these opposing effects, we generated a *LOX3* knockout construct (Δ*Lox3*) in rice (*Oryza sativa* L.) plants. Blast resistance and transcription levels of rice genes in Δ*Lox3* rice plants and the effects of exogenous jasmonic acid (JA) on resistance and transcriptional levels of rice genes in *Magnaporthe oryzae-*infected Δ*Lox3* rice plants were further elucidated. The results showed that the Δ*Lox3* plants exhibited normal phenotypes, with high levels of methyl-linolenate and reactive oxygen species (ROS), and the genes involved in three Kyoto Encyclopedia of Genes and Genomes (KEGG) pathways contributed to rice seed quality. *M. oryzae*-infected Δ*Lox3* plants exhibited serious blast symptoms with a reduced defense response but increased ROS-mediated cell death, and the genes involved in seven KEGG pathways contributed to rice seed quality. Exogenous JA treatment alleviated blast symptoms in infected Δ*Lox3* plants by hindering hyphal expansion, inhibiting ROS-mediated cell death, and increasing the defense response, and genes involved in 12 KEGG pathways contributed to rice seed quality. These findings demonstrate that *LOX3* plays an important role in rice growth and defense, and its knockout improves rice quality at the expense of disease resistance. Exogenous JA provides a means to compensate for the reduction in defense responses of *LOX3* knockout rice lines, suggesting potential applications in agricultural production.

## 1. Introduction

The growth in the global population and the consequent increase in food production demand necessitate an increase in global food supply by 60–100% by 2050 [1]. However, the challenge of maintaining global crop yield and food security is threatened by a wide range of pathogens that cause widespread diseases and epidemics [2]. Sustainable chemical controls and the use of disease-resistant crop cultivars with high yields and good quality are effective and economical approaches to alleviate crop yield losses [2]. However, some genes, such as *PigmR* and *OsLOX10*, known to improve rice resistance to various stresses, have been found to simultaneously reduce rice yield or seed quality [3,4]. Therefore, it is a significant challenge to balance yield, quality, and disease resistance through plant breeding [5]. Nevertheless, some elicitors can be exogenously applied to enhance plant resistance and maintain a balance between quality and resistance. For example, plant defense hormones, including salicylic acid (SA) and jasmonic acid (JA), can be exogenously applied to activate defense responses to enhance plant resistance [6,7]. Hence, the exogenous application of phytohormones may be a feasible solution to balance the trade-off between yield, quality, and disease resistance of crops.

Endogenous signals such as SA, JA, and ethylene (ET) can activate plant immunity and improve plant defense against disease [8,9,10]. The JA signaling pathways induce the expression of defense-related genes, stimulate the production of antimicrobial compounds such as reactive oxygen species (ROS) and peroxidase (POD), and cause the deposition of lignin and callose [11,12,13]. The deposition of lignin provides a physical and chemical barrier to protect plant cells from pathogen invasion [14]. Moreover, callose deposition between the cell wall and plasma membrane at the pathogen infection site and the plasmodesmata can slow the invasion and spread of pathogens [15]. The transcription of some defense-related genes, such as *OsWRKY45*, *OsPR1a*, and *OsbHLH35,* is induced by both JA and infection with *Magnaporthe oryzae*, a fungus that causes rice blast disease [16,17,18,19,20,21,22,23,24]. Biotrophy-associated secreted protein 4 (BAS4) is considered crucial in the biotrophic invasion of *M. oryzae* [25,26], while host cell death is induced by *M. oryzae* cell death-inducing protein 1 (MoCDIP1), which is only expressed during the necrotrophic phase [27]. ROS production mediated by *OsRbohB* results in host cell death. However, a moderate ROS concentration serves as a second messenger, which induces plant defense, while excessive ROS causes cell damage during abiotic or biotic stresses [28,29]. This issue can be controlled through the use of both POD and ROS-scavenging systems that neutralize the excessive ROS generated in plant cells [30]. Based on the above findings, as JA is a key regulator in providing defense against pathogens [31], researchers have focused on the influence of JA on rice quality and disease resistance.

In the initial synthesis step of jasmonate, which occurs in plastids, lipoxygenases (LOXs) catalyze the deoxygenation of fatty acids through the oxidation of polyunsaturated fatty acids such as α-linolenic acid and linoleic acid [32], converting them into peroxide derivatives that can act as signaling molecules to regulate plant growth, development, and stress response [33]. LOXs also play important roles in plant development and response to biotic stress and pathogen resistance [34,35]. Consequently, the disruption or deletion of *LOX* genes can block JA synthesis, therefore affecting plant development and stress responses. In rice embryos, three isozymes, *LOX3*, *LOX2*, and *LOX1*, were identified and found to be active in the bran fraction of the seed and the embryo [36,37]. Previous studies have shown that antisense *LOX3* suppression in rice endosperm enhanced rice seed longevity [38,39]. However, it also decreased rice resistance to drought, bacterial blight, and rice blast [34,40]. Additionally, other studies have revealed that *LOX3* is involved in disease resistance through the analysis of antisense rice plants of *LOX3* or *LOX3* gene identification in infected rice plants by *M. oryzae* [34,40], indicating that *LOX3* antisense suppression results in a trade-off between improved seed quality and decreased resistance in rice. However, there has been a lack of research on how to alleviate the decrease in plant resistance, therefore achieving a balance between resistance and quality. Although JA and SA can be exogenously applied to activate defense responses to enhance plant resistance [6,7], there is no report that exogenous JA can balance the trade-off between seed quality and resistance in rice, and the effect of JA on rice blast disease differs depending on the method or timing of JA treatment [41,42].

To examine the influence of *LOX3* knockout on rice resistance and seed quality during *M. oryzae* infection of rice, some experiments, including cytological, physiological, and molecular analyses of the *LOX3* knockout rice line (Δ*Lox3*) inoculated with the rice blast strain, were performed. To reveal the quality of Δ*Lox3* rice seeds, rice seed germination and the contents of methyl-linoleate (LA) and methyl-linolenate (ALA) were evaluated. Exogenous JA was applied to Δ*Lox3* rice lines at either 36 or 48 h post-inoculation (hpi) with the strain of *M. oryzae* to reveal the exogenous application of JA to balance seed quality and decreased resistance trade-off during *M. oryzae* infecting Δ*Lox3* rice. We further focused on defense-related and metabolic pathways and screened the key differentially expressed genes (DEGs) between 48 hpi Δ*Lox3* rice lines with or without JA treatment using RNA-seq analysis. Furthermore, the contributions of genes and KEGG pathways to rice seed quality were analyzed accordingly [43]. Our results provide insight into the molecular mechanism underlying the decreased resistance and improved seed quality in rice caused by *LOX3* knockout, as well as valuable information about the use of elicitors to balance the trade-off between improved seed quality and decreased resistance.

## 2. Materials and Methods

### 2.1. Construction of LOX3 Knockout Rice Lines

The Δ*Lox3* rice lines were generated using the wild-type (WT) cultivar (*Oryzae sativa* ssp. *japonica* cv. Ilmibyeo), which is a Korean domestic rice cultivar developed from the three-way cross of Milyang’96/Milyang’95/Seomijinbyeo [44]. The virulent rice blast strain 95234I-1b, capable of causing severe rice blast disease symptoms in Ilmibyeo, was maintained in the laboratory, as reported previously [41,45].

To knockout *LOX3,* Δ*Lox3* was produced by using CRISPR-P (http://crispr.hzau.edu.cn/CRISPR2/, accessed on 15 September 2021) for the design of the sgRNA guide sequence. The sgRNA1 and sgRNA2 templates for Δ*Lox3* were annealed to *LOX3* using two primers, 5′-TGTGTGCTCCTCGACAACGTCCATGGTTT-3′/5′-TCTAAAACCATGGACGTTGTCGAGGAGCA-3′ and 5′-GGCGGCTCACAGTGAGGTTCAACT-3′/5′-AAACAGTTGAACCTCACTGTGAGC-3′, and cloned into *BsaI*-digested MP-01DS (Abbkine, Wuhan, China) to construct the sgRNA expression vector, MP-01DS-sgRNA1/sgRNA2. After proper confirmation of the knockout vector by sequencing, the pMP-01DS-sgRNA1/sgRNA2 plasmid was transformed into *Agrobacterium tumefaciens* EHA105 and rice (Ilmibeyo) callus cells [41,46].

Independent transgenic lines (#2, #9, and #12) were achieved and confirmed by sequencing with the primer OsU6-SF2 (AGAGGCGGGAGGAACAGTTT). Genomic DNA was extracted from the rice plants using the hexadecyltrimethylammonium bromide (CTAB) method [47] and served as a template for sequencing through first-generation sequencing technology [48]. The sequence results of the mutated region from three rice transgenic lines revealed the presence of double peaks at the same position, representing mutations. The sequencing results of three Δ*Lox3* transgenic rice lines are displayed in Figure S1. Target specificity was evaluated through CRISPR-P (http://cbi.hzau.edu.cn/cgi-bin/CRISPR, accessed on 15 September 2021), and no potential off-target sites were verified (Figure S1E). Additionally, we conducted PCR amplification to check *Cas9* in three Δ*Lox3* rice plants using the primers (F: GTGGCCTATTCTGTGCTGGT, R: ATCCCGGTGCTTGTTGTAGG). The PCR results showed that all three transgenic rice plants displayed a 483 bp PCR product (Figure S1F). We selected the three transgenic lines from the T_1_ generation for investigation. We collected and stored this material in our laboratory.

### 2.2. Growth of Rice Plants, M. oryzae Inoculation, Microscopic Observation and Biomass Analysis

Seeds of the T1 generation of Δ*Lox3* rice lines (#2, #9, and #12) and WT were surface-sterilized for 20 min with 75% ethanol and hypochlorite (2%), respectively. These seeds were then germinated in sterile water, utilizing the protocol of Liu et al. [49]. The germinated seeds were then sown in paddy soil:humus (3:1) in a greenhouse at 28 °C/26 °C (day/night) with 16 h light (approximately 250 mmol m^−2^ s^−1^)/8 h dark at a relative humidity (RH) of 50%. Each treatment was performed in triplicate, and ten randomly selected plants from each treatment were analyzed for agronomic characteristics, including plant height and tiller number, from 90-day-old rice plants. We also analyzed 30 randomly selected rice seeds for germination percentage and 100 randomly selected rice seeds for 100-grain weight. Fourteen-day-old rice seedling leaves of the three Δ*Lox3* rice lines and WT were collected to measure POD, ROS, lignin, methyl-linoleate, methyl-linolenate, and defense-related gene expression.

*Magnaporthe oryzae* strain 95234I-1b, which causes rice blast disease on rice plants, was cultured as previously described [41]. Hyphae were collected from potato dextrose agar (PDA) medium in a petri dish (containing 200 g glucose, 10 g sugar, and 15 g agar powder per liter of sterile water). After mycelial growth covered the petri dish, the surface mycelium was scraped away, and the spores were produced in darkness for three days and then washed from the petri dish using sterilized water to prepare the spore suspension. Plant inoculation was conducted following Duan et al. [45]. Briefly, 14-day-old rice plants with three leaves and a total of 180 leaves from 60 rice plants were inoculated with the above-prepared spore suspension (1 × 10^5^/mL) and incubated at 28 °C for 24 h before being transferred to a greenhouse. The inoculated leaves were collected to measure dead cells, callose, POD, ROS, lignin, and defense-related gene expression. At 168 hpi, 120 plants were chosen randomly for the evaluation of disease severity.

To further investigate the infection process, *M. oryzae* strain 95234I-1b was inoculated into rice sheaths. Briefly, the 95234I-1b spore suspension was used to inoculate leaf sheaths of 40-day-old rice plants, which were then kept in the dark at 28 °C. They were then assessed at 24, 36, and 48 hpi using the propidium iodide (PI) staining method for detecting dead cells, as described by Jones et al. [50]. A 1× PI working solution was prepared by diluting 10× assay buffer (catalog #KTA1001, Abbkine, Wuhan, China) with deionized water and warming it to 37 °C before use. The PI-stained samples were examined utilizing a fluorescence microscope (DM 2000, Leica, Wetzlar, Germany) to detect primary mycelia and secondary hyphae.

For punch inoculation, the leaves of 40-day-old rice plants were lightly punched, and 10 µL of spore suspension (3 × 10^5^/mL) was injected into the wound. The inoculated leaves were kept moist in a dark box with a relative humidity of 100%. At 144 hpi, lesion lengths were measured using a ruler. DNA was extracted from a single lesion of an infected leaf using a Micro Extraction Kit (G2N70, Sigma‒Aldrich, St. Louis, MO, USA). Relative fungal biomass within the lesion was assessed using DNA-based quantitative PCR (qPCR) [51] and was defined as the ratio of the *M. oryzae Pot2* gene level relative to that of the rice ubiquitin gene (*OsUBQ*). PCR was performed following the amplification cycle parameters: initial denaturation for 3 min at 95 °C, 44 denaturation cycles at 95 °C for 20 s, and annealing extension at 59 °C for 20 s, with the fluorescence signal detected at 65 °C. The melt curve parameters involved a temperature increase from 58 °C, with the fluorescence signal recorded for 80 cycles, increasing by 0.5 °C per cycle. The relative quantity of fungal DNA was determined by comparing the cycle threshold (C_T_) of *M. oryzae Pot2* DNA to the Ct of rice genomic ubiquitin DNA. There were three sets of each inoculation experiment.

### 2.3. Exogenous JA Treatment of Inoculated Rice Leaves

The method for JA treatments and its concentration referred to Wang et al. [52]. At 36 or 48 hpi, inoculated WT and Δ*Lox3* rice leaves were individually sprayed with JA (5 mL, 50 µM) in 50 mM sodium phosphate buffer. As a control, leaves were sprayed with buffer only (5 mL). At 168 hpi, blast symptoms of these rice leaves (referred to hereafter as 36 hpi JA-treated and 48 hpi JA-treated rice samples) were investigated. The JA + *M. oryzae*-infected 36 hpi rice samples were collected at 36, 48, and 72 hpi, whereas the JA + *M. oryzae*-infected 48 hpi rice samples were collected at 48, 72, and 96 hpi. These samples were used to investigate blast symptoms, measure dead cells, callose, POD, ROS, lignin, and expression of defense-related genes.

### 2.4. Callose Deposition and Dead Cell Analysis

The staining methods for callose and dead cells were based on Wang et al. [41] and were repeated three times. To observe callose deposition and dead cells, 20 freshly inoculated young leaves or JA + *M. oryzae*-infected 36 hpi and JA + *M. oryzae*-infected 48 hpi young leaves of each sample were studied. To analyze callose deposition, fresh leaves were cleared with 95% ethanol and incubated in an ethanol-emulsifiable solution (glycerol:phenol:water:ethanol:lactic acid = 1:1:1:8:1) at 65 °C until the removal of the green color. The leaves were then washed with 50% ethanol, followed by sterilized water. The number of deposited callose was counted in 20 fields of vision after staining the treated leaves with 0.1% aniline blue for 1 h. Observations and photographs were taken using a DMI4000B fluorescence inverted microscope (Leica Camera AG, Wetzlar, Germany).

To observe dead cells, fresh leaves were cleaned with sterile water and incubated in 100% ethanol until the complete removal of the green color. The leaves were then stained for 24 h in DAB (3,3-diaminobezidin, 1 mg/mL, pH 5.8). The number of dead cells was measured through photographic analysis of 20 separate visual fields.

### 2.5. Determination of ROS Content, Lignin Content, and POD Activity

The ROS content was determined using an enzyme-linked immunosorbent assay (ELISA) kit (Jiangsu Enzyme Immunity Industry Co., Ltd., Yancheng, China), and the OD_450_ nm was measured utilizing a Varioskan LUX-type multifunctional enzyme instrument (Thermo Fisher, Waltham, MA, USA). Each biochemical analysis was repeated three times.

The lignin content was determined according to Lee et al. [53] with slight modifications. In brief, rice leaves (0.1 g) were ground into a powder in liquid nitrogen, and 900 mL of PBS (pH 7.2) was added to the resulting suspension and then centrifuged for 20 min at 12,000 rpm before collecting the supernatants.

POD activity was determined using a POD activity detection kit (catalog #BC0095; Beijing Solarbio Science & Technology Co., Ltd., Beijing, China) and the Varioskan LUX-type multifunctional enzyme instrument accordingly [54]. Using a change in OD_470_ nm per minute of 0.05 as the relative enzyme activity unit, POD activity was calculated.

### 2.6. GC‒MS Quantification of Methyl-Linoleate and Methyl-Linolenate Contents of Rice Leaves under MRM Condition

Detection of methyl-linoleate and methyl-linolenate was performed using multiple reaction monitoring (MRM) (Shanghai Applied Protein Technology Co. Ltd., Shanghai, China). One gram of rice leaves or *M. oryzae*-infected 48 hpi rice leaves was mixed with chloroform (4 mL) in a clean EP tube, vortexed for 30 s, and 2 mL of 0.9% NaCl solution was added and vortexed for 30 s. Then, the resulting mixture was centrifuged, and the lower layer of liquid was transferred to another test tube, followed by the addition of 2 mL of dichloromethane and centrifugation for 15 min. Then, the lower liquid was blow-dried with nitrogen, 2 mL of methanol (containing 5% sulfuric acid) was added, and the mixture was placed in a water bath for 2 h at 80 °C. After cooling to room temperature, the samples were mixed with *n*-hexane (2 mL) and 1 mL of water by vortexing for 30 s and centrifuged, and the supernatant was removed. Water (1 mL) was added to the samples, mixed again, and centrifuged. The supernatant was removed and dried with nitrogen, and an appropriate volume of isooctane was added according to the sample concentration. The resulting solution was vortexed for 30 s, left to stand for 5 min, and transferred to a sample bottle for detection.

Gas chromatography‒mass spectrometry (GC‒MS) analysis was carried out utilizing an Agilent Technologies 7820A gas chromatography system coupled to a 5977B inert mass selective detector (MSD) quadrupole mass spectrometer (Agilent Technologies, Santa Clara, CA, USA) and an HP-5MS capillary column coated with polyimide (30 m × 0.25 mm, 0.25 μm film thickness; Agilent Technologies). The samples (1 μL aliquots) were injected into the column in a splitless manner to separate the electronic circulation folder (ECF)-derivatized free fat acids (FFAs). High concentrations of FFA were analyzed in a second run by injecting an aliquot (1 μL) in 1:10 split mode. The oven temperature was initially set at 100 °C for 5 min, followed by a temperature increase to 240 °C at 4 °C/min and maintenance for 15 min. The mass selective detector had a scan range of 30–450 amu and an ionization potential of 70 eV. MS detection was performed using an electrospray ionization (ESI) source in negative ion mode under the following conditions: source temperature, 500 °C; ion source gas 1, 45; ion source gas 2, 45; curtain gas, 30; ion spray voltage floating, −4500 V. MRM transitions were as follows: methyl-linolenate, *m/z* 286 → 201*;* methyl-linoleate, *m/z* 286 → 181. ESI evaluations were conducted in triplicate, and compounds were identified by searching the NIST05 mass spectral database and co-eluting with appropriate standards.

### 2.7. HPLC‒ESI‒MS/MS Analysis of JA, JA-Ile, and SA Contents in Rice Leaves

High-performance liquid chromatography–electrospray ionization/mass spectrometry (HPLC**‒**ESI**‒**MS/MS) (Shanghai Applied Protein Technology Co. Ltd.) was used to determine the concentrations of SA, JA, and JA-Ile in rice leaves, *M. oryzae*-infected 48 hpi rice leaves, and JA + *M. oryzae*-infected 36 hpi and 48 hpi rice plants. To extract the rice leaves, frozen plant material (100 mg) was mixed with ice-cold aqueous acrylonitrile (ACN) (1 mL, 50%, *v*/*v*) and sonicated for 3 min at 4 °C. The samples were then extracted using a benchtop laboratory rotator for 30 min at 4 °C. The supernatant was collected through centrifugation (12,000 rpm, 10 min, at 4 °C), transferred to sterile plastic microtubes, and washed with 1 mL of MeOH and deionized water. C18 reversed-phase, polymer-based, solid-phase extraction (RP-SPE) cartridges were equilibrated with aqueous ACN (50%, *v*/*v*) and used to purify the samples. After collecting a sample from the loaded cartridge, a final rinsing with 1 mL of 30% ACN (*v*/*v*) was performed. Subsequent to one-step SPE, the samples were dried and frozen at −20 °C for later analysis.

For HPLC**‒**ESI**‒**MS/MS analysis, the samples were dissolved in ACN (200 μL, 30% *v*/*v*) and transferred to insert-equipped vials. Sample extracts were analyzed using a Vanquish UPLC-QE-Orbitrap-MS system. The parameters used for analysis were as follows: UPLC: column, Waters ACQUITY UPLC HSS T3 (1.8 μm, 2.1 mm × 50 mm); injection volume, 2 μL; flow rate, 0.3 mL/min; column temperature, 40 °C; solvent system, water (0.1% acetic acid):ACN (0.1% acetic acid); gradient program, 90:10 *v*/*v* at 0 and 1.0 min, 10:90 *v*/*v* at 5.0 and 7.0 min, 90:10 *v*/*v* at 7.1 and 9.0 min. High-Resolution Mass Spectroscopy (HRMS) data were acquired using the selected-ion monitoring (SIM) acquisition methods on a Q Exactive hybrid Q-Orbitrap mass spectrometer with a heated ESI source (Thermo Fisher Scientific). The following are the ESI source.

Parameters: sheath gas pressure, 40 arb; spray voltage, −2.8 kV; sweep gas pressure, 0 arb; auxiliary gas pressure, 10 arb; auxiliary gas heater temperature, 350 °C; and capillary temperature, 320 °C. Data were acquired using Xcalibur 4.1 (Thermo Scientific, Waltham, MA, USA) and processed using TraceFinder 4.1 (Thermo Scientific), with the quantitative information exported to Excel.

### 2.8. Real-Time Quantitative PCR (RT-qPCR)

To analyze gene expression, RT-qPCR was used to measure the expression levels of rice genes (POD-related gene of *OsPOX1*, ROS production gene of *OsRbhoB*, JA response or synthetic genes of *OsbHLH35* and *OsLOX3*, and SA response genes of *OsWRKY45* and *OsPR1a*) in rice leaves, and these six rice defense-related genes and *M. oryzae* genes (*MoBAS4* and *MoCDIP1*) in *M. oryzae*-inoculated leaves and JA + *M. oryzae*-infected 36 hpi and 48 hpi rice plants. For rice leaves, three-leaf stage seedlings from WT and Δ*Lox3* transgenic rice lines were collected. For *M. oryzae*-inoculated leaves, inoculation was performed using a 95234I-1b spore suspension (3 × 10^5^ spores/mL) on WT and Δ*Lox3* transgenic rice line (three-leaf stage) seedlings, followed by the collection of fresh leaves at 0, 24, 36, 48, 72, and 96 hpi. The RNAiso Plus Kit (TransGen Biotech., Beijing, China) was used to extract total RNA from 100 mg of freshly harvested rice leaves that had been ground into a powder using liquid nitrogen. The isolated RNA was reverse transcribed into cDNA using TransScript All-in-One First-Strand cDNA Synthesis SuperMix (TransGen). RT-qPCR was performed on a CFX96 Real-Time System (Bio-Rad, Hercules, CA, USA) using a TransStart Top Green qPCR SuperMix kit (TransGen). The relative expression ratio was calculated using the 2^−∆∆Ct^ method, as described by Wang et al. [41]. The primer pairs were constructed using genes from both *M. oryzae* and rice and presented in Table S1.

### 2.9. RNA-seq Analysis

RNA-seq analysis was performed to determine the gene expression profile of leaf samples from the WT and Δ*Lox3* transgenic rice line (#2), *M. oryzae*-inoculated rice leaves of WT and #2 at 48 hpi, JA + *M. oryzae*-infected 36 hpi WT and #2 (collected at 36, 48 and 72 hpi), and JA + *M. oryzae*-infected 48 hpi WT and #2 (collected at 48, 72 and 96 hpi). Each treatment was performed with three biological replicates. The cDNA libraries were constructed and sequenced following the manufacturer’s instructions (MetWare Biological Science and Technology Co., Ltd., Wuhan, China), with a typical insert size of 250 bp. The Illumina HiSeq 2000 platform (Illumina, San Diego, CA, USA) was used for cDNA sequencing.

Raw reads with more than 10% unknown bases or shorter than 20 bases were discarded, and adapter sequences were removed from the remaining reads. The clean reads were then mapped to the reference genome sequence of *M. oryzae* 70-15 (version 8, Magnaporthe Comparative Sequencing Project, Broad Institute of Harvard and MIT). Differentially expressed genes (DEGs) were analyzed using DESeq2 or edgeR (3.5.1), with the criteria for screening DEGs set at adjusted *p* value (padj) < 0.05 and |log2 (fold change)| > 1. The DEGs were then subjected to enrichment analyses of gene ontology (GO) and Kyoto Encyclopedia of Genes and Genomes (KEGG) pathways using the DAVID tool (Fisher’s exact test, *p* values ≤ 0.05) [55].

### 2.10. Statistical Analysis

IBM SPSS Modeler 17.0 software (http://www-01.ibm.com/software/analytics/spss/, accessed on 20 October 2022) was used to analyze all data, with the results expressed as the mean ± standard deviation (SD). One-way analysis of variance (ANOVA) followed by Bonferroni correction was employed to identify statistically significant differences between WT and transgenic lines. All tests were two-sided, with each letter representing a significant difference at the *p* < 0.05 level. SigmaPlot 10.0 (https://systatsoftware.com/, accessed on 20 October 2022) was utilized to generate graphical representations.

## 3. Results

### 3.1. Effects of LOX3 Knockout on Rice Agronomic Traits, Antimicrobial Compounds, and Defense-Related Gene Expression

**Figure 1:**
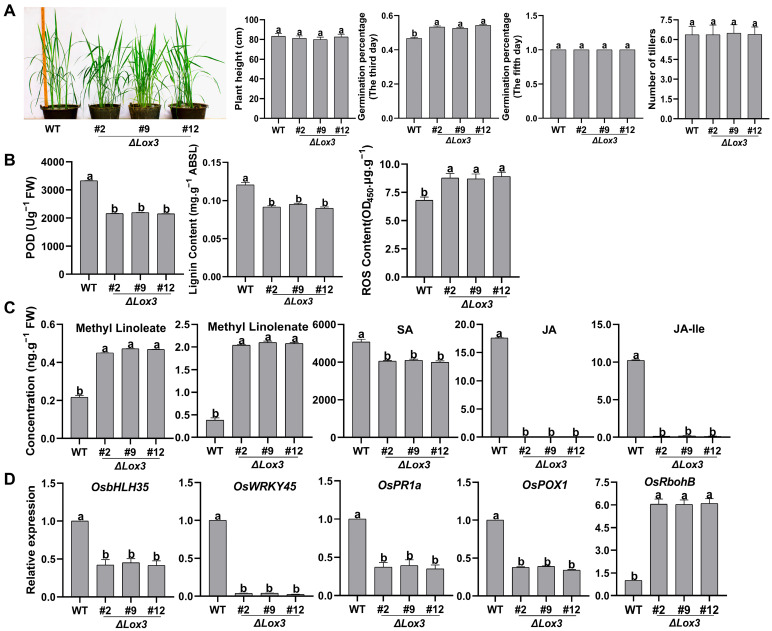
Phenotype, physiological traits, and defense-related gene expression in three *LOX3* knock -out rice lines: (**A**) Plant height, seed germination on the third and fifth days of seed soaking, and tiller number. (**B**) POD activity, lignin content, and ROS content. (**C**) Contents of the compounds methyl-linoleate and methyl-linolenate and hormones SA and JA. (**D**) Expression levels of some defense-related genes by RT-qPCR. All data are expressed as the mean ± standard deviation (SD). Different lowercase letters above the bars indicate significant differences (*p* < 0.05). Abbreviations: WT, wild type; FW, fresh weight; POD, peroxidase; ABSL, acetyl bromide spectrophotometric lignin; ROS, reactive oxygen species; SA, salicylic acid; JA, jasmonic acid; JA-Ile, JA-isoleucine.

The *LOX3* knockout gene construct (Δ*Lox3*) was introduced into rice using *Agrobacterium tumefaciens-*mediated transformation. Three T_1_ transgenic rice lines (#2, #9, and #12) and WT plants were grown in a greenhouse for further analysis. *LOX3* expression was not detected in the T1, T2, and T3 generations of the three Δ*Lox3* transgenic rice lines (Figure S2). The effects of *LOX3* knockout on the agronomic traits of rice were investigated. The germination percentage of seeds from the three Δ*Lox3* rice lines was obviously higher than that of the WT by the third day of seed soaking, but all seeds from the WT and Δ*Lox3* could fully germinate by the fifth day. These results indicated that the germination speed of Δ*Lox3* was faster than that of WT. The plant height, tiller number, and 100-grain weight of the three Δ*Lox3* rice lines were not significantly different from those of the WT (Figure 1A and Figure S3), demonstrating that the elimination of *LOX3* accelerated rice seed germination without any penalty in plant height, number of tillers, or 100-grain weight.

The levels of antimicrobial compounds, including POD, lignin, and ROS, were next measured, and it was found that the POD activity and lignin content of the three *LOX3* knockout lines were significantly lower than those in WT, while the ROS content was notably higher (Figure 1B). No JA or JA-Ile was detected in the three *LOX3* knockout lines, and the SA content markedly decreased. Moreover, higher levels of methyl-linoleate (LA) and methyl-linolenate (ALA) were observed than in the WT (Figure 1C). These results indicated that these two compounds were not converted to peroxide derivatives, and no JA was synthesized in *LOX3* knockout transgenic rice lines. Additionally, the effect of *LOX3* knockout on the expression of defense-related genes was further evaluated, revealing that it suppressed the expression of *OsbHLH35*, *OsWRKY45*, *OsPR1a*, and *OsPOX1* but upregulated the expression of *OsRbohB* (Figure 1D). These results suggested that *LOX3* knockout improved rice quality by increasing germination speed and the levels of LA and ALA and influencing the expression of multiple defense-related genes. Because of the similar phenotypes and physiological traits in the three lines, we randomly selected #2 for further experiments.

### 3.2. Reduced Resistance to Rice Blast in ΔLox3 Transgenic Rice Plants

**Figure 2:**
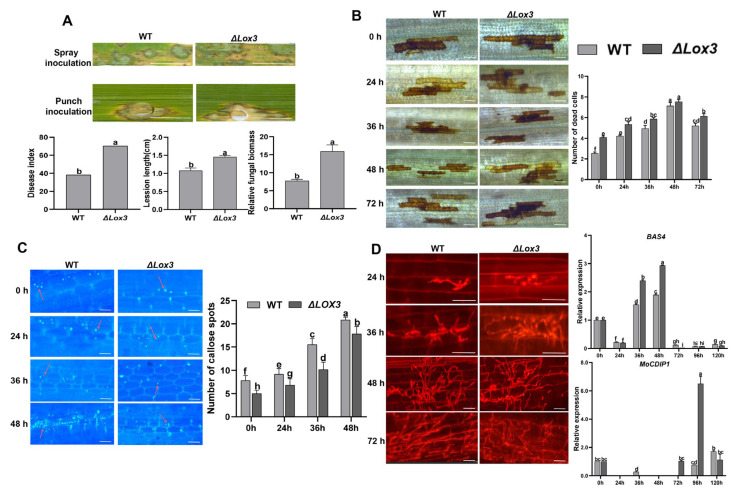
Rice blast symptom, detection of callose deposition and cell death, and infection process during *M. oryzae* infecting rice: (**A**) Image showing differential blast symptoms on infected WT and infected #2 (left) and quantitative data for disease index, lesion length, and relative fungal biomass of a single lesion (right). Scale bars, 1 mm. (**B**,**C**) Image of dead cells and callose deposition (left), with their corresponding quantitative data (right). Scale bars, 50 µm. (**D**) Images of the infection process (left) and expression of *MoBAS4* and *MoCDIP1* by RT-qPCR (right). Scale bars, 25 µm (24 and 36 h) and 50 µm (48 and 72 h). All data are expressed as the mean ± SD. Different lowercase letters above the bars indicate significant differences (*p* < 0.05). In panel C, red arrows indicate callose dots that were counted.

To evaluate the resistance of Δ*Lox3* transgenic rice plants against rice blast disease, we inoculated the leaves of one Δ*Lox3* rice line (#2) and WT plants with *M. oryzae* and observed more severe blast symptoms and increased disease indexes in the infected Δ*Lox3* plants relative to WT. Punch inoculation also resulted in larger lesions and increased fungal biomass in Δ*Lox3* plants compared to WT plants (Figure 2A). These results revealed that *LOX3* knockout increased rice susceptibility to rice blast fungus.

To further understand the mechanism underlying the increased susceptibility of Δ*Lox3* plants to rice blast, we observed a continuous increase in dead cells, with more such cells in infected Δ*Lox3* plants than in WT plants (Figure 2B). Callose deposition also continued to increase upon infection in both Δ*Lox3* and WT plants but to a lesser extent in Δ*Lox3* (Figure 2C). As the infection progressed, secondary hyphae from primary mycelia emerged in both groups at 36 hpi, and they were more abundant at 48 hpi. The expression of the *M. oryzae* effector gene *BAS4* was higher at 36–48 hpi in infected Δ*Lox3* than in WT, with the highest expression at 48 hpi, consistent with the emergence of more secondary hyphae at 36 and 48 hpi. However, the expression of *MoCDIP1* was lower at 72–120 hpi in infected WT than in Δ*Lox3* (Figure 2D), consistent with the differences in the number of dead cells (Figure 2B).

**Figure 3:**
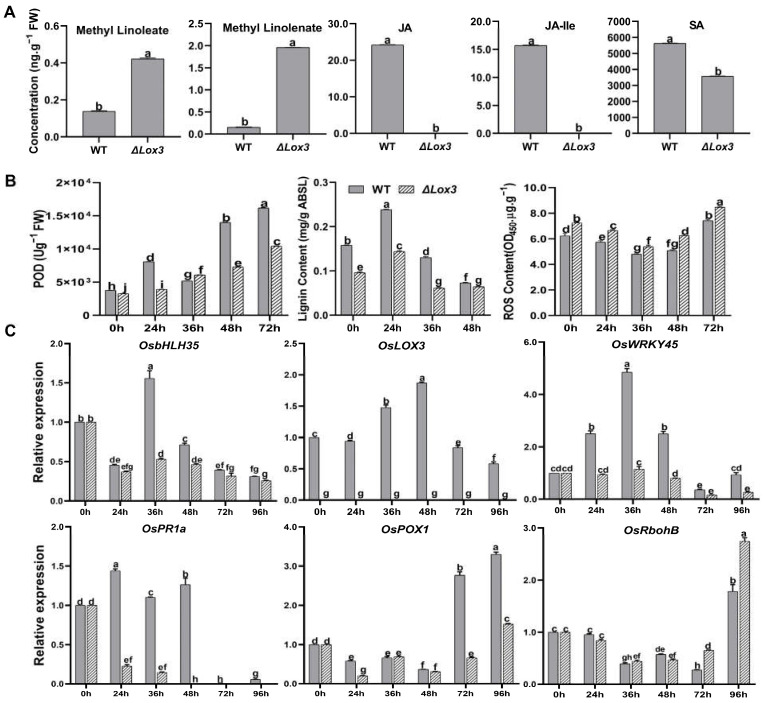
Quantitative analysis of plant defense compounds and hormones, physiological traits, and defense-related genes during *M. oryzae* infecting rice. (**A**) Contents of methyl-linoleate, methyl-linolenate, JA, JA-Ile, and SA. (**B**) POD activity, lignin content, and ROS content. (**C**) Expression levels of JA-related and defense-related genes. All data are expressed as the mean ± SD. Different lowercase letters above the bars indicate significant differences (*p* < 0.05).

The contents of LA and ALA in *M. oryzae*-infected 48 hpi Δ*Lox3* rice plants were higher than those in *M. oryzae*-infected 48 hpi WT rice plants (Figure 3A). In contrast, JA or JA-Ile was not detected in *M. oryzae*-infected 48 hpi Δ*Lox3*, whereas both compounds were highly abundant in infected WT (Figure 3A), indicating that *M. oryzae*-induced JA biosynthesis in WT rice plants but not in Δ*Lox3* rice plants. Furthermore, we analyzed the levels of the antimicrobial compounds POD, lignin, and ROS and found that the POD activity and lignin content were higher in infected WT than in Δ*Lox3*, whereas the ROS content was lower (Figure 3A,B). This indicates that JA, POD, and lignin did not function in the defense response of infected Δ*Lox3* plants, while the significant increase in ROS levels led to many cell deaths, contributing to the colonization of *M. oryzae* hyphae.

We next analyzed JA-related and defense-related gene expression and found that *LOX3* was not expressed in Δ*Lox3* plants but was highly expressed in infected WT plants. *OsbHLH35*, *OsWRKY45*, *OsPOX1*, and *OsPR1a* had lower expression levels in infected Δ*Lox3* than in infected WT, whereas *OsRbohB* was more highly expressed in infected Δ*Lox3* than in infected WT (Figure 3C).

### 3.3. Influence of Exogenous JA on Rice Blast Resistance of the ΔLox3 Transgenic Rice Line

**Figure 4:**
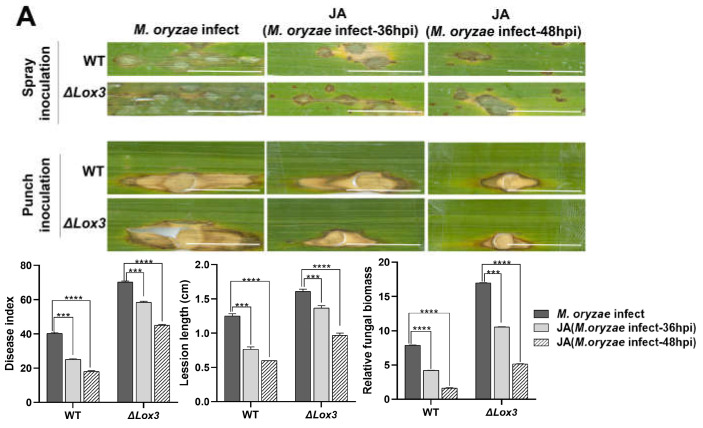

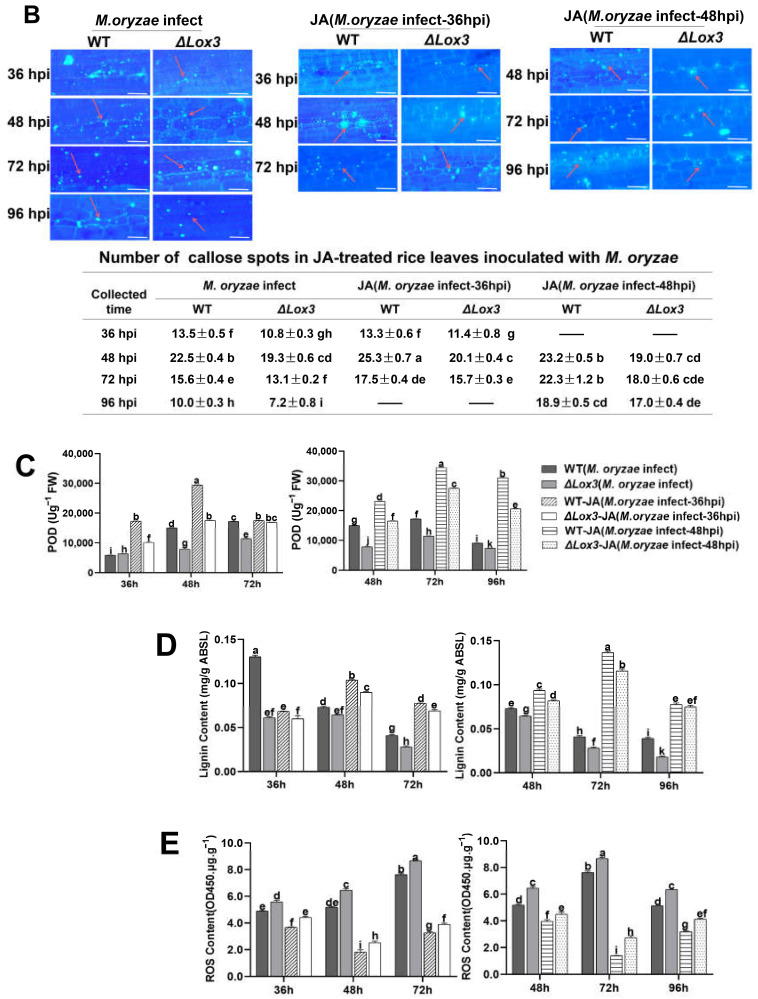

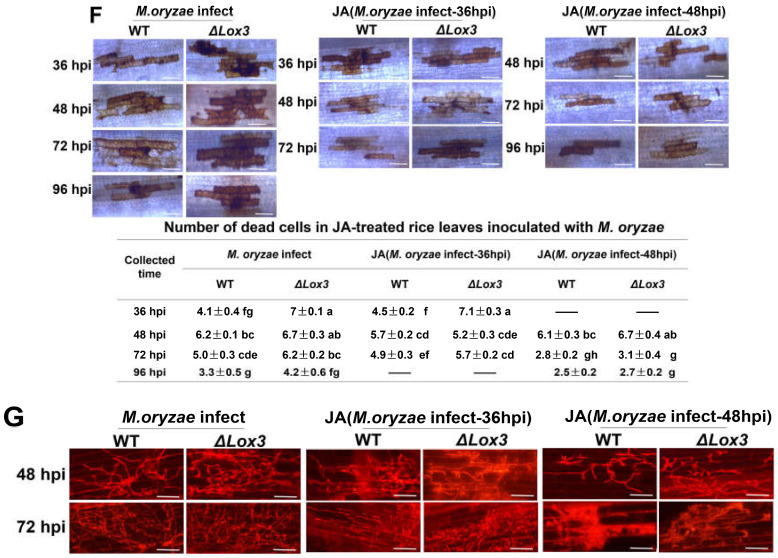
Effects of exogenous JA application on rice blast symptoms, callose deposition, cell death, POD activity, ROS and lignin contents, and the infection process in rice plants infected with *M. oryzae*. (**A**) Blast symptoms, disease index, lesion length, and relative fungal biomass of a single lesion. Scale bar, 1 mm. More stars indicate higher significance. (**B**) Callose deposition. Scale bars, 50 µm. Red arrows indicate the location of callose dots that were counted. (**C**) POD activity. (**D**) Lignin content. (**E**) ROS content. (**F**) Cell death number. Scale bars, 50 µm. (**G**) Images of the infection process over time. Scale bars, 50 µm. All data are expressed as the mean ± SD. Different lowercase letters above the columns indicate significant differences (*p* < 0.05).

To analyze whether exogenous JA can enhance the resistance of the Δ*Lox3* transgenic rice line (#2) to *M. oryzae*, exogenous JA (50 µM) was applied to either *M. oryzae*-infected rice leaves at 36 hpi or 48 hpi. The results showed that exogenous JA effectively decreased blast symptoms at both timepoints in *M. oryzae*-infected WT and Δ*Lox3* plants compared to *M. oryzae*-infected rice leaves without exogenous JA treatment (Figure 4A). The impact of exogenous JA on rice blast symptoms was significantly greater at 48 hpi than at 36 hpi in both infected WT and Δ*Lox3* plants. However, the symptom-alleviating effect of JA was greater in infected WT than in Δ*Lox3* plants, as confirmed by the punch-inoculation results (Figure 4A).

We also analyzed the effects of exogenous JA on dead cell number and the production of callose, POD activity, ROS, and lignin contents during *M. oryzae* infection. Our results revealed that exogenous JA induced callose deposition and increased lignin content and POD activity in both WT and Δ*Lox3* at 36 and 48 hpi, but the effects were greater in WT than in Δ*Lox3* (Figure 4B–D). Conversely, analyses of ROS content and dead cell numbers showed contrasting results at the same stages of *M. oryzae* infection, with more dead cells and ROS detected in Δ*Lox3* than in WT (Figure 4E,F). Additionally, we assessed the influence of exogenous JA on the *M. oryzae* infection process by applying it to rice sheaths infected with *M. oryzae* at 36 and 48 hpi. Our findings indicate that JA effectively inhibited mycelial expansion and fungal colonization in rice cells, with significant inhibition of mycelial development in JA + *M. oryzae*-infected 48 hpi rice (WT and Δ*Lox3*) (Figure 4G).

**Figure 5:**
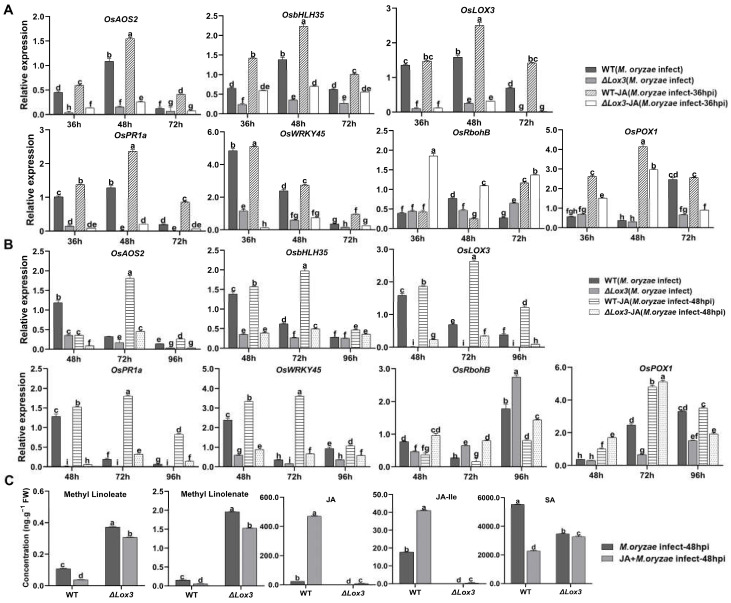
Effects of JA application on the expression of genes related to SA and JA signaling pathways and plant defense, and the contents of associated compounds in *M. oryzae*-infected WT and Δ*Lox3* rice line (#2). (**A**,**B**) Expression levels of genes related to methyl-linoleate, methyl-linolenate, and JA following JA application in *M. oryzae*-infected rice at 36 and 48 hpi. (**C**) Contents of methyl-linoleate, methyl-linolenate, JA, JA-Ile, and SA in JA + *M. oryzae*-infected 48 hpi WT and Δ*Lox3* (#2). All data are expressed as the mean ± SD. Different lowercase letters above the bars indicate significant differences (*p* < 0.05).

We subsequently determined the expression levels of genes related to JA, SA, POD, and ROS in *M. oryzae*-infected 36 hpi and 48 hpi WT and Δ*Lox3* rice after exogenous JA treatment. The results revealed that JA treatment triggered significant upregulation of the JA-related genes *OsAOS2*, *OsLOX3*, and *OsbHLH35* and SA-related genes *OsPR1a* and *OsWRKY45* in *M. oryzae*-infected 36 hpi and 48 hpi WT plants but not in *M. oryzae*-infected 36 hpi and 48 hpi Δ*Lox3* plants (Figure 5A,B). In addition, JA treatment induced upregulation of *OsRbohB* in both infected groups at both timepoints, but to a greater degree in infected Δ*Lox3* than in infected WT plants at both timepoints (Figure 5A,B), which was consistent with the results of ROS content (Figure 4E). Similarly, JA induced upregulation of the POD-related gene *OsPOX1* in 36 hpi and 48 hpi *M. oryzae*-infected WT rice and Δ*Lox3*, and a higher expression level appeared in *M. oryzae*-infected 48 hpi Δ*Lox3* than in *M. oryzae*-infected 36 hpi WT and Δ*Lox3* and 48 hpi WT rice (Figure 5A,B). As there was JA-related gene expression induction by exogenous JA in Δ*Lox3*, and exogenous JA was more effective in alleviating blast symptoms of *M. oryzae*0infected 48 hpi WT and Δ*Lox3* plants than 36 hpi infected plants (Figure 4A), we further examined whether exogenous JA could modulate levels of JA, LA, ALA, JA-Ile, and SA in *M. oryzae*-infected 48 hpi WT and Δ*Lox3* plants. The results showed that the levels of ALA and LA were significantly decreased, but JA-Ile and JA distinctly increased in JA + *M. oryzae*-infected 48 hpi WT. However, there was no change in the contents of ALA and LA, but the JA and JA-Ile contents were slightly elevated in JA + *M. oryzae*-infected 48 hpi Δ*Lox3* rice plants. Also, the SA content in JA + *M. oryzae*-infected 48 hpi WT, and Δ*Lox3* increased compared with infected WT and Δ*Lox3* (Figure 5C and Figure 1C), indicating that exogenous JA failed to induce activation of JA defense responses in infected Δ*Lox3*. JA mitigated rice blast symptoms mainly by limiting hyphae expansion and colonization in infected Δ*Lox3* plants.

### 3.4. Different Treatments Impacted Genes Contributing to Rice Blast Resistance and Rice Quality

**Figure 6:**
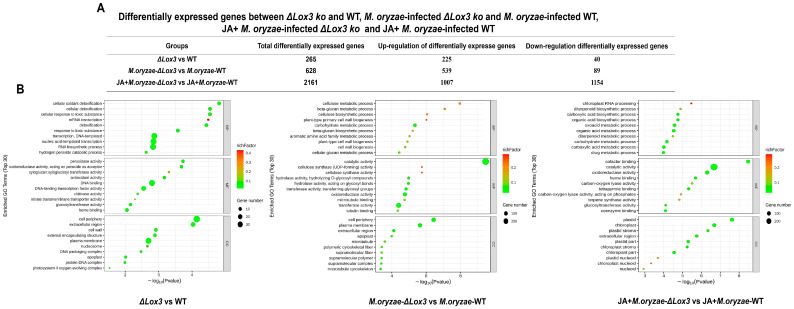
Effects of *LOX3* knockout, *M. oryzae* infection, and exogenous JA on gene expression. (**A**) Differentially expressed genes (DEGs) between Δ*Lox3* and WT; *M. oryzae*-infected Δ*Lox3* and *M. oryzae*-infected WT; and JA + *M. oryzae*-infected Δ*Lox3* and JA + *M. oryzae*-infected WT. (**B**) GO enrichment categories of these DEGs.

**Figure 7:**
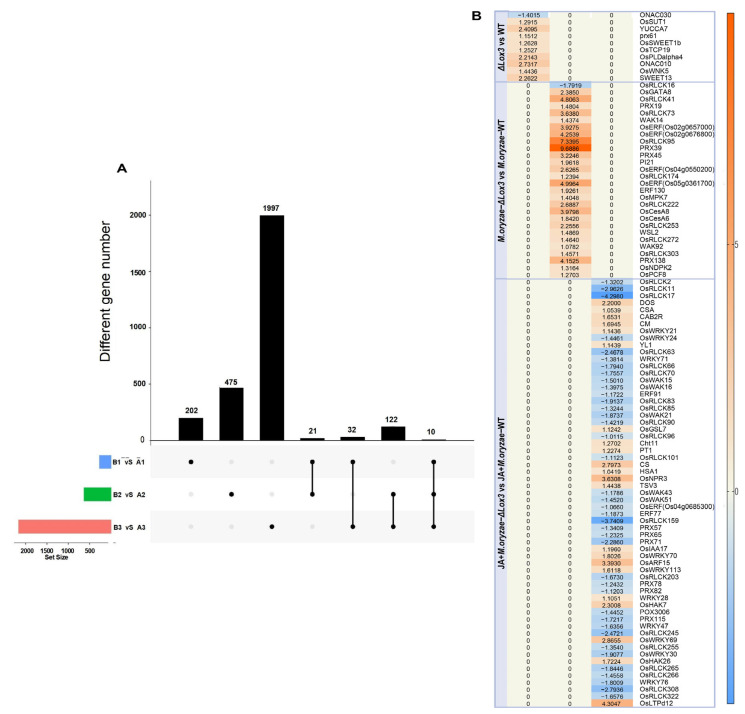
Common and specific DEGs in Δ*Lox3* vs. WT, *M. oryzae*-infected Δ*Lox3* vs. *M. oryzae*-infected WT, and JA + *M. oryzae*-infected Δ*Lox3* vs. JA + *M. oryzae*-infected WT: (**A**) Common and specific genes in three different comparisons. (**B**) Specific DEG analysis of three different comparisons. A1: WT; A2: *M. oryzae*-infected WT; A3: JA + *M. oryzae*-infected WT; B1: Δ*Lox3*; B2: *M. oryzae*-infected Δ*Lox3*; B3: JA + *M. oryzae*-infected Δ*Lox3*. The black spots: gene set of DEGs belonging to a comparison. The black line: interconnected black dots indicate that there are common differentially expressed genes in two or three comparisons anchored by black dots. Column chart: the number of differentially expressed genes intersected in each comparison.

We performed large-scale transcriptome analyses on the *OsLOX3* knockout rice line, *M. oryzae*-infected Δ*Lox3 rice* line, and JA + *M. oryzae*-infected Δ*Lox3* rice line. In the Δ*Lox3* vs. WT comparison, we detected 265 DEGs, of which 225 were upregulated, and 40 were downregulated (Figure 6A). Among the DEGs selected based on the top 30 items sorted by *p* value under three GO categories, most played roles in cellular oxidant detoxification and types of enzyme activities, etc. (Figure 6B). We further found upregulation of *OsFAD2*, *SWEET13,* and *OsPsbR2* (Figure 7A,B; Table S2), revealing that *LOX3* knockout induced the expression of these genes that are mainly involved in rice growth and increased antioxidant capacity, indicating that the Δ*Lox3* rice line had good quality.

In the *M. oryzae*-infected Δ*Lox3* vs. *M. oryzae*-infected WT comparison, we detected 628 DEGs, of which 539 were upregulated, and 89 were downregulated (Figure 6A). These DEGs primarily played roles in catalytic activity, cellular metabolic processes, and the cell periphery (Figure 6B). We found eight upregulated DEGs, including *OsGATA8*, *OsPCF8*, *OsNDPK2*, and five *ERF* genes, which may be involved in regulating rice growth, as well as 20 other DEGs, including four *PRXs*, eight *OsRLCKs*, *WAK92*, *WSL2*, *OsCesA6*, *OsCesA8*, *OsMPK7*, *WAK14*, and *PI21*, that may play roles in defense (Figure 7A,B; Table S2). These results suggested that several genes played major roles in defense, but only a few were involved in rice growth in infected Δ*Lox3* plants, indicating that *LOX3* knockout led to an imbalance between growth and resistance.

In the JA + *M. oryzae*-infected Δ*Lox3* vs. JA + *M. oryzae*-infected WT comparison, we identified 2161 DEGs, of which 1007 were upregulated, and 89 were downregulated (Figure 6A). The DEGs were primarily involved in cofactor binding and function in plastids and chloroplasts (Figure 6B). Most of the upregulated DEGs, including *CAS*, *DOS*, *CAB2R*, *CS*, *CM*, *HSA1*, *TSV3*, *YL1*, *PT1*, *OsIAA17*, *OsARF15*, *OsHAK7*, *OsHAK26*, *OsLTPd12*, and *OsWRKY21* (Figure 7A,B; Table S2), were involved in rice growth, and several DEGs, including *OsWRKY21*, *OsWRKY28*, *WRKY70*, *OsWRKY69*, *OsWRKY113*, *OsGSL7*, *Cht11*, and *OsNPR3*, were involved in rice defense. Interestingly, we found that 19 *OsRLCK* genes and six *PRX* genes were downregulated compared with those in infected Δ*Lox3* plants. These results suggested that exogenous JA triggered the transcription of more genes that functioned in rice growth or defense, indicating that JA can balance the trade-off between growth and resistance.

### 3.5. Contributions of KEGG Pathways to Rice Blast Resistance and Rice Quality

**Figure 8:**
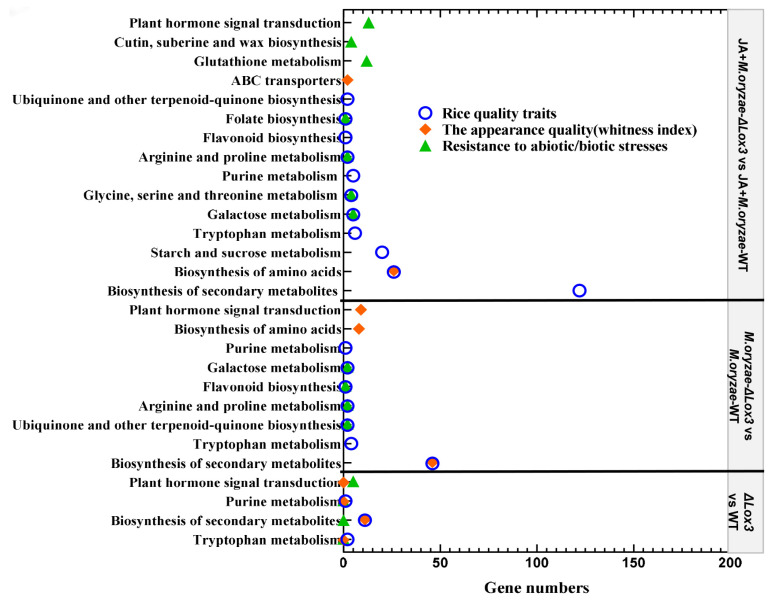
Contribution of gene number and KEGG pathways to rice quality and resistance.

We mainly analyzed the contributions of KEGG pathways to rice seed quality based on the metabolic pathways described by Chen et al. [27]. It was found that tryptophan metabolism, secondary metabolite biosynthesis, and purine metabolism contributed to rice quality traits and appearance quality, as well as enhancing plant hormone signal transduction function in defense in the Δ*Lox3* rice line (Figure 8). Seven KEGG pathways, including biosynthesis of secondary metabolites, ubiquinone and other terpenoid-quinones, tryptophan metabolism, proline and arginine metabolism, flavonoid biosynthesis, galactose metabolism, and purine metabolism, contributed to rice quality traits. Biosynthesis of secondary metabolites and amino acids contributed to the appearance quality, and biosynthesis pathways of ubiquinone and other terpenoid-quinone and flavonoid biosynthesis contributed to energy metabolism and carbon assimilation. Moreover, signal transduction of plant hormone, proline, and arginine metabolism, flavonoid biosynthesis, galactose metabolism, and biosynthesis pathways of ubiquinone and other terpenoid-quinones played a role in resistance during *M. oryzae* infection of Δ*Lox3* rice plants (Figure 8).

When exogenous JA was applied to the *M. oryzae*-infected Δ*Lox3* rice line, twelve KEGG pathways were involved in rice quality traits, including arginine and proline metabolism; secondary metabolite and amino acid biosynthesis; tryptophan metabolism; sucrose and starch metabolism; galactose metabolism; purine metabolism; serine, threonine, and glycine metabolism; flavonoid biosynthesis; folate biosynthesis; biosynthesis of ubiquinone; and other terpenoid-quinones. It was observed that the biosynthesis of amino acids and secondary metabolites and ABC transporters contributed to rice appearance quality, and ubiquinone and other terpenoid-quinone biosynthesis pathways and flavonoid biosynthesis contributed to energy metabolism and carbon assimilation. Moreover, cutin, suberin, and wax biosynthesis; glutathione, galactose, arginine, proline, glycine, serine, and threonine metabolisms; flavonoid biosynthesis; and plant hormone signal transduction played roles in resistance (Figure 8).

**Figure 9:**
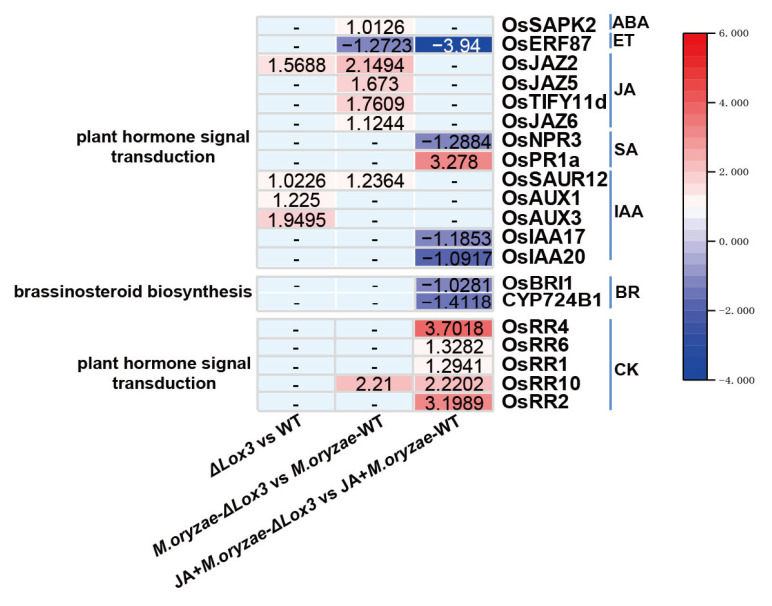
The DEGs enriched in plant hormone signal transduction and brassinosteroid biosynthesis in the Δ*Lox3* vs. WT comparison, *M. oryzae*-infected Δ*Lox3* vs. *M. oryzae*-infected WT comparison, JA + *M. oryzae*-infected Δ*Lox3* vs. JA + *M. oryzae*-infected WT comparison.

As *LOX3* is involved in the initial step of JA biosynthesis, changes in JA content may impact other plant hormones. Consequently, we further focused on genes involved in two KEGG pathways, plant hormone signal transduction and brassinosteroid (BR) biosynthesis. We found that these genes functioned in signals of JA, SA, ET, IAA (auxin), ABA (abscisic acid), CK (cytokinin), or BR (Figure 9). In comparing Δ*Lox3* rice plants with WT rice plants, four genes (*OsSAUR12*, *OsAUX3*, *OsAUX1*, and *OsJAZ2*) were enriched in plant hormone signal transduction and functioned in IAA and JA signals. These genes were upregulated, indicating that JA defense was suppressed, but IAA signaling was activated. In the comparison of *M. oryzae*-infected Δ*Lox3* rice plants and *M. oryzae*-infected WT rice plants, eight genes were enriched in plant hormone signal transduction, functioning in signals of JA, IAA, ABA, CK, and ET. Among them, *OsJAZ2*, *OsJAZ5, OsTIFY11d*, *OsJAZ6, OsSAUR12*, *SAPK2*, and *OsRR10* were upregulated, but *OsERF87* was downregulated, indicating that JA defense and ET signal were suppressed, but IAA, ABA, and CK signals were activated. In the comparison of JA + *M. oryzae*-infected Δ*Lox3* rice plants and JA + *M. oryzae-*infected WT rice plants, 11 genes were enriched in plant hormone signal transduction, and one gene was enriched in BR biosynthesis. These genes functioned in signals of SA, IAA, JA, CK, ET, and BR. Among them, three genes (*OsJAZ2*, *OsJAZ5*, and *OsJAZ6)* were repressed, six genes (*OsPR1a*, *OsRR4*, *OsRR6*, *OsRR1*, *OsRR10*, and *OsRR2)* were upregulated, and five genes (*OsNPR3*, *OsIAA17*, *OsIAA20*, *OsBRI1*, and *CYP724B1)* were downregulated, indicating that JA defense, SA, and CK signals were activated, but BR and IAA signals were suppressed. These results revealed that *LOX3* knockout significantly affected the signals of plant hormones, including JA, SA, ET, IAA, ABA, CK, and BR, leading to the suppression of JA defense, but exogenous JA was able to restore JA defense in *M. oryzae*-infected Δ*LOX3* rice plants.

## 4. Discussion

Lipoxygenases (LOXs) are key enzymes in the biosynthesis of JA [56] (Figure S4) and play important roles in growth, development, and stress responses in plants. Previous studies have demonstrated that decreased *LOX3* expression can alleviate drought stress, defend against pathogens in rice plants [40], and enhance the longevity and storage stability of rice seeds [38,39]. In this study, we observed that *LOX3* knockout significantly decreased rice resistance to rice blast fungus, which is consistent with the findings of Liu et al. [40]. The germination speed of Δ*Lox3* rice seeds was significantly improved compared with that of WT seeds, in line with the findings of Bollinedi et al. [38]. Linoleic and α-linolenic acids play multifaceted roles in plants, including defense regulation [57], seed development and quality [58,59,60], and influencing the economic value of oil crops [61]. In this work, although plant height, tiller number, and 100 g grain weight remained unchanged in the Δ*Lox3* rice line, LA and ALA levels accumulated significantly in the Δ*Lox3* rice plants, and Δ*Lox3* rice seeds exhibited faster germination speed than those of WT plants, indicating potentially superior seed quality. Our future studies will further measure more seed quality indicators, such as starch and nutrient contents, for a comprehensive evaluation of seed quality. We further explored the effects of *LOX3* knockout on the innate immunity of rice and found that POD activity, lignin content, JA-Ile, and JA decreased, while ROS content and dead cells increased in the Δ*Lox3* rice line. Moreover, there was a downregulation of JA-related genes, indicating that *LOX3* knockout affected the innate immunity of rice, especially when the JA defense was blocked. In conclusion, *LOX3* knockout enhanced the germination speed of rice seeds, but it also seemed to damage the plants’ immune systems, as reflected by decreases in POD activity and lignin content and downregulation of defense-related genes. We further found that the levels of ALA and LA in infected Δ*Lox3* were similar to those in uninfected Δ*Lox3*, and there was no JA, low levels of SA, POD activity and lignin, and low expression levels of JA- and SA signal genes in infected Δ*Lox3*, compared with those in infected WT.

Some studies have demonstrated that *OsLOX1* and *OsLOX2* can also contribute to the production of JA through the 13-lipoxygenase pathway [37,62,63]. However, *OsLOX3* has been identified as the primary isozyme among the three isozymes of *OsLOX1*, *OsLOX2,* and *OsLOX3*, and the expression of the *OsLOX3* antisense gene not only downregulated *OsLOX3* expression but also *OsLOX1* and *OsLOX2* [39]. Here, *OsLOX3* was knocked out in rice, which possibly significantly affected the expression of *OsLOX1* and *OsLOX2*, although we did not detect their expression. We further analyzed RNA-seq data and found that *OsJAZ2* was upregulated in Δ*Lox3* rice plants compared to WT rice plants, and *OsJAZ2*, *OsJAZ5,* and *OsJAZ6* were upregulated in *M. oryzae*-infected Δ*Lox3* rice plants compared to *M. oryzae*-infected WT rice plants. However, *OsJAZs* were repressed in JA + *M. oryzae*-infected Δ*Lox3* rice plants compared to JA + *M. oryzae*-infected WT rice plants, which revealed that JA defense was suppressed in Δ*Lox3* rice plants and *M. oryzae*-infected Δ*Lox3* rice plants, indicating that no JA or JA-Ile was detected in Δ*Lox3* rice plants or *M. oryzae*-infected Δ*Lox3* rice plants. Therefore, we speculated that *OsLOX1* and *OsLOX2* were not involved in JA or JA-Ile biosynthesis. These results revealed that *LOX3* knockout inhibited JA defense responses without impairing rice growth, indicating that seeds had good quality but decreased resistance.

JA and SA act as elicitors that induce similar defense responses in plants [64]. Specifically, JA plays a vital role in plant resistance against necrotrophic pathogens [65]. The widespread expansion of thin secondary hyphae marks the beginning of the necrotrophic phase in hemibiotrophic fungi [66]. Based on the optimum concentration of exogenous JA for controlling rice blast disease reported by Wang et al. [52], we applied 50 µM exogenous JA to *M. oryzae*-infected Δ*Lox3*, which resulted in a slight increase in endogenous JA, as well as an elevation in lignin content and POD activity, but a decrease in ROS content and the number of dead cells, compared with those of JA + *M. oryzae*-infected WT rice plants. The levels of LA and ALA were similar in infected and uninfected Δ*Lox3* rice plants, indicating that exogenous JA could not induce activation of JA and SA defense responses in the infected Δ*Lox3* rice line. Thin secondary hyphae began to develop from primary hyphae at 36 hpi, while extensive growth of many thin secondary hyphae was observed at 48 hpi during *M. oryzae* infection of both WT and Δ*Lox3* rice plants, showing that 48 hpi was the temporal onset of the necrotrophic phase of *M. oryzae*. We also found that exogenous JA more effectively decreased rice blast symptoms in *M. oryzae*-infected rice at 48 hpi than at 36 hpi, indicating that JA could hinder the extensive growth of secondary hyphae in infected rice tissue. Previous work has shown that JA antagonizes ROS-induced cell death [67], revealing that exogenous JA alleviates rice blast symptoms in infected Δ*Lox3* mainly by inducing an increase in POD activity and lignin content and inhibiting ROS-induced cell death. Ding et al. [68] described that exogenous methyl-JA attenuated the effect of *LOX3* mutation and enhanced plant tolerance to stress damage. Our results demonstrated that exogenous JA could alleviate rice blast symptoms in the infected Δ*Lox3* rice line, mainly by hindering extensive growth of secondary hyphae and inhibiting ROS-induced cell death, without penalty of rice growth, indicating that exogenous JA not only controlled rice blast disease but also maintained good growth status in the infected Δ*Lox3* rice line.

Previous research demonstrated that antisense suppression of *LOX3* improved the longevity of rice seeds [39]. In addition, three pathways (tryptophan metabolism, biosynthesis of secondary metabolites, and purine metabolism) that contribute to rice quality and appearance traits were identified [69]. In the Δ*Lox3* rice line, we found the upregulation of a Δ12-fatty acid desaturase (*OsFAD2*), sucrose transporters (*SWEET13* and *OsSUT1*), and *OsPsbR2,* which play a role in photosynthesis. In addition, RNA-seq data showed the activation of genes in the abovementioned three pathways, revealing that *LOX3* knockout improved rice growth and indicating that Δ*Lox3* seeds possess good quality. In the *M. oryzae-*infected Δ*Lox3* rice line, we found the upregulation of cell-wall-related genes, including *WAK92* (wall-associated kinase), *WSL2* (synthesis of leaf cuticular wax), *OsCesA6* (cellulose synthase), *OsCesA8* (cellulose synthase), *OsMPK7* and *WAK14* (wall-associated kinases), and *PI21* (proline-rich protein), indicating that these genes function in rice defense. However, four upregulated *PRX* genes were similar to peroxidase when combined with the results of high ROS content and more dead cells at the late stage of infection, which indicated that *PRXs* played an important role in ROS production. Excessive ROS can lead to lots of cell death, which contributes to *M. oryzae* uptake of nutrition from dead cells and continuous colonization of rice, indicating that *PRXs* played a negative role in the defense of the Δ*Lox3* rice line and might be one of the important factors that resulted in a resistance decrease in the infected Δ*Lox3* rice line. A few upregulated genes (*OsGATA8*, *OsPCF8*, *OsNDPK2*, and five *ERF* genes) functioned in leaf greening, chloroplast development, and chlorophyll biosynthesis, which were found in the infected Δ*Lox3* rice line, revealing that these genes tended to be involved in rice growth. In exogenous JA-treated *M. oryzae*-infected Δ*Lox3* rice plants, we found more upregulated genes involved in chloroplast development, nutrient transport, and lipid transfer, indicating that these genes function in rice growth (including *CM*, *DOS*, *CSA*, *CAB2R*, *YL1*, *HSA1*, *CS*, *TSV3*, *OsIAA17*, *OsHAK7*, *OsHAK26*, and *OsLTPd12*). However, only a few upregulated genes played roles in rice defense (*WRKY21*, *WRKY28*, *WRKY70*, *WRKY69*, *WRKY113*, *CHT11*, *OsNPR3*, and *OsGSL7*), while 19 *OsRLCK* genes and six *PRX* genes were downregulated. These findings revealed that *OsRLCK* and *PRX* genes showed opposite changes between the infected *LOX3* knockout rice line and the JA-treated *M. oryzae-*infected *LOX3* knockout rice line, revealing that the expression of *OsRLCKs* and *PRXs* was negatively regulated by JA. Combined with the results of decreased ROS and fewer dead cells, we speculated that JA could effectively alleviate the symptoms of rice blast disease mainly through the negative regulation of *PRXs*-mediated ROS production in JA-treated *M. oryzae*-infected Δ*Lox3* rice plants. Thus, exogenous JA improved rice growth by activating the transcription of growth-related genes while enhancing rice resistance mainly by downregulating *PRX* genes to decrease ROS-mediated cell death. The biosynthesis pathways of ubiquinone and other terpenoid-quinones have been reported to exert a role in energy metabolism, oxidative stress, and carbon assimilation [70]. Here, we also detected these pathways in JA-treated *M. oryzae*-infected Δ*Lox3* rice plants, revealing that these pathways can function in rice growth and *M. oryzae* resistance, indicating that exogenous JA can activate some pathways or genes to function in rice growth and defense.

To discover the impact of *LOX3* knockout on the biosynthesis, signal, or response of plant hormones, two KEGG pathways of plant hormone signal transduction and brassinosteroid biosynthesis were screened. In Δ*Lox3* rice plants, *LOX3* knockout affected the signals of IAA and JA. In *M. oryzae*-infected Δ*Lox3* rice plants, the signals of JA, IAA, ABA, CK, and ET were affected. Exogenous JA application affected the signals of SA, IAA, JA, CK, ET, and BR in *M. oryzae*-infected Δ*Lox3* rice plants. Furthermore, *OsJAZs* were found to be upregulated in both Δ*Lox3* rice plants and *M. oryzae-*infected Δ*Lox3* rice plants, indicating that JA defense was suppressed. However, *OsJAZs* were repressed in JA + *M. oryzae*-infected Δ*Lox3* rice plants, indicating that JA defense was restored by exogenous JA application. These findings suggested that *LOX3* knockout led to the suppression of JA biosynthesis and JA defense, significantly impacting the biosynthesis, signals, or response of other plant hormones in rice. These results highlight the central role played by JA signaling in plant hormone crosstalk [71,72,73].

## 5. Conclusions

When taking these results together, *LOX3* knockout rice plants exhibited good growth status, which indicated that *LOX3* knockout seeds had good quality and could provide necessary sources for rice plants to grow well, even during *M. oryzae* infection of rice leaves. Many key genes, such as *OsRLCKs* and *PRXs*, could be used as targets for gene engineering to improve crop varieties. Although *LOX3* knockout decreased blast resistance and suppressed JA biosynthesis and response, leading to affected biosynthesis or signals of other plant hormones, including SA, IAA, ABA, CK, ET, and BR, exogenous JA balanced the trade-off between resistance reduction and seed quality improvement caused by *LOX3* knockout in rice. Our results provide valuable information for rice resistance breeding and reveal a potential application prospect for balancing rice seed quality and resistance through exogenously applying environmentally friendly elicitors, such as JA, in agricultural production.

## Data Availability

Data are available from authors.

## Supplementary Materials

The following supporting information can be downloaded at: https://www.mdpi.com/article/10.3390/biom13081197/s1, Figure S1: The mutant allele and sequences of *LOX3* knockout rice lines by the first-generation sequencing technique; Figure S2: Expression of *LOX3* in T_1_ generations of *LOX3* knockout transgenic rice lines and WT; Figure S3: The 100 g grain weight of three Δ*Lox3* transgenic rice lines and WT; Figure S4: JA biosynthesis through octadecanoid pathway in plants. Table S1: Primers used in this study; Table S2. Specific DEGs in #2 vs. WT, *M. oryzae-*infected #2 vs. *M. oryzae*-infected WT, JA + *M. oryzae*-infected #2 vs. JA + *M. oryzae-*infected WT.
