# Supplementary material for: Exogenous Jasmonic Acid Alleviates Blast Resistance Reduction Caused by LOX3 Knockout in Rice
Source: Biomolecules. 2023 Jul 31;13(8):1197. doi: 10.3390/biom13081197 (PMC10452216; doi:10.3390/biom13081197)
Supplement: Supplementary file 1 [file biomolecules-13-01197-s001.zip › biomolecules-2478080-supplementary.pdf]

biomolecules

MDPI

Article

Exogenous Jasmonic Acid Alleviates Blast Resistance Reduction Caused by *LOX3* Knockout in Rice

Shunyu Su [1,2,†], Ping Tang [1,2,†], Rubin Zuo [1,2], Hongfeng Chen [1,2], Tianqi Zhao [1,2], Shumin Yang [1,2] and Jing Yang [1,2,*]

1 State Key Laboratory for Conservation and Utilization of Bio-Resources in Yunnan, Yunnan Agricultural University, Kunming 650201, China; 2021210313@stu.ynau.edu.cn (S.S.); 2001015@ynau.edu.cn (P.T.); 2022210344@stu.ynau.edu.cn (R.Z.); 2020210289@stu.ynau.edu.cn (H.C.); 2021210323@stu.ynau.edu.cn (T.Z.); 2019311106@stu.ynau.edu.cn (S.Y.)

2 Key Laboratory of Agro-Biodiversity and Pest Management of Ministry of Education, Yunnan Agricultural University, Kunming 650201, China

* Correspondence: yangjing@ynau.edu.cn

† These authors contributed equally to this study.

A

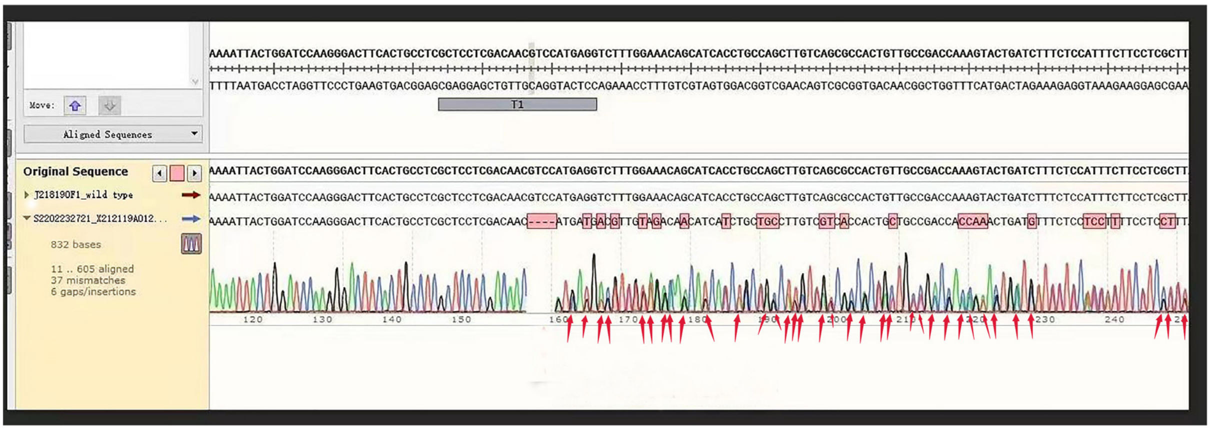

B

>*ΔLox3* #2
ACTGCCTACTTTTTGTCCTGACTACAGTATCACAGAATGCTGGGAGGGCTCAAGGACAAGCTGACAGGGAA
AAATGGAAATAAGATCAAAGGCTTGGCTGTGCTGATGAGCCGAAAATTACTGGATCCAAGGGACTTCACTG
CCTCGCTCCTCGACAACATGATGACGTTGTAGACAACATCATCTGCTGCCTTGTCGTCACCACTGCTGCCGAC
CACCAAACTGATGTTTCTCCTCCTTTTCCTCCTTTACTACTTTTGATGATGTACATGATGGCTGTGATGAAAAA
ATAATCGATGATCACTCCTCACTCCTCACAAATTATAGAGAAACAACGAGGAGACAGCCAGCGACGACACTG
ATGAGACTACTCTACTGTTGTATATGCTGTGATGAGACAACGATGACGGGGGGATGATAGGAGCATAAGAGA
ATATGCAGCAGGGGGTGACGAATTAACCACCATCATCATCACGAGAGGACACCATCATCACGATGATGATAA
CGGGAAAATGACCAACATGGTGGTGCCAGGCCCATCTTCTTCAGAACCACCACCAGAACCAGGTCCTTCTT
CAGAACAATAACCTTCAAGGTGGCCCTGGTTGTACATATGTGTTGTGCCACTCCTGGATCTACCCTGTGGAAA
GACCACTACATCTATCTCTTGCAACATGAATCAGATGAATAACCAGCTATTACACCACCGCTCTTCTGGTCTGG
CAGAATCTCCTATCTGGTAGTAATCTGAATATCACTCTGATGCTAGTACGACTGATGACTATATATATCATCGATC
ATACTAGTAAGTCGCATAGTCTACATACATCGA

C

>*ΔLox3* #9
TTGCCTTCATAATTGTTACTGTACTACAGTATCACAGAATGCTGGGAGGGCTCAAGGACAAGCTGACAGGGA
AAAATGGAAATAAGATCAAAGGCTTGGCTGTGCTGATGAGCCGAAAATTACTGGATCCAAGGGACTTCACT
GCCTCGCTCCTCGACAACGTCATGAGGTCTTTGGAAACAGCATCACCTGCCAGCTTGTCAGCGCCACTGTTG
CCGACCAAAGTACTGATCTTTCTCCATTTCTTCCTCGCTTACTTTGCATGAGTTTGTACATGTTTGCTATGTAGA
AAGAATCAAGGATCAGTACTCAGTACTCAGCACAGATTGAACGGAAACAAGGTGAAAACGGCCAGTGACA
CAAATGAAGTGACTAACCTATTGTCTTATATGCTATGTGCAGACAATGAGGGCAGGGGGATAGTTGGATCAG
AGGCGAATCTGGAGCAGGGGTTGACAGATTTACCATCGGTATCACAAGGGGAGTCCAAGCTCACAGTGAG
GTTCACTGTGAGATATGGACAAATGTGGTGTGCCGGGGGCCATCATCCTCAAAAACCACCACTACACCAAGC
TCTTCCTCAAGACCATCCCCCTCCGTGATGCCCGTGTGTGAGACTCTATTGTGTGTGGTGCCCTCTCCTGTATC
TACCGTGGTGGAAAGCACCACTACTATCATATCTTCTTTGCACACTGTGAAAGTCGAGAGAGAATAGCGAGT
CTATACACCACCACGTCTCTCTTTGTGCTCTTGAGATATCTTCATATTGTGTATAGTGATATCGAGAGTATTATAA
CATCGTGAAAGCGTATATTTTACGCGATAGAGAGTGACTATATTATATTTTAAATACTCCTCGATCATATACTTAA
AAAAAAGCTCTGATTAAATCTCAACAAAAAAAAAAGCAAAAAAATT

D

>*ΔLox3* #12
ACGCTACTTTTGTCCTGACTACAGTATCACAGAATGCTGGGAGGGCTCAAGGACAAGCTGACAGGGAAAAA
TGGAAATAAGATCAAAGGCTTGGCTGTGCTGATGAGCCGAAAATTACTGGATCCAAGGGACTTCACTGCCT
CGCTCCTCGACAACGTCATGAGGTCTTTGGAAACAGCATCACCTGCCAGCTTGTCAGCGCCACTGTTGCCGA
CCAAAGTACTGATCTTTCTCCATTTCTTCCTCGCTTACTTTGCATGAGTTTGTACATGTTTGCTATGTAGAAAG
AATCAAGGATCAGTACTCAGTACTCAGCACAGATTGAACGGAAACAAGGTGAAAACGGCCAGTGACACAA
ATGAAGTGACTAACCTATTGTCTTATATGCTATGTGCAGACAATGAGGGCAGGGGGATAGTTGGATCAGAGG
CGAATCTGGAGCAGGGGTTGACAGATTTACCATCGGTATCACAAGGGGAGTCCAAGCTCACAGTGAGGTTC
ACTGGGAGATGGACAAGCATGGTGTGCCAGGGGCCATCATCATCAAGAACCACCACAGTACCAAGTTCTTC
CTCAAGACCATCACCCTCCATGATGTCCCTGGCTGTGACACTATTGTGTTTGTTGCCAACTCCTGGATCTACCC
TGTTGGAAAGTACCACTACAATCGTATCTTCTTTGCAAACAATGTAAGTCAGGAGTGATTAGCAAGTCTAAAT
ACCACAACGCCTCCCTTTCTGTTCTTGGCAGATCTTCTTATCCTGTTTAAGGTTATCGTGAGTAATTTAACATCT
TGTAAGTGCATAGTTTAAACGATAGTGAATGACTATATTATATTTAATTAATCATCGATCATATACTTAGTAAAAA
GTTCTGCAATTAAGCTCACACATCATCATACGATAGAGAAAACCTT

Figure S1. The mutant allele and sequences of *LOX3* knockout rice lines by the first-generation sequencing technique. (A) Sequencing result of *LOX3* knockout rice line #2. (B, C and D) The sequences from three *LOX3* knockout rice lines #2, #9 and #12. Red arrows: double peak.

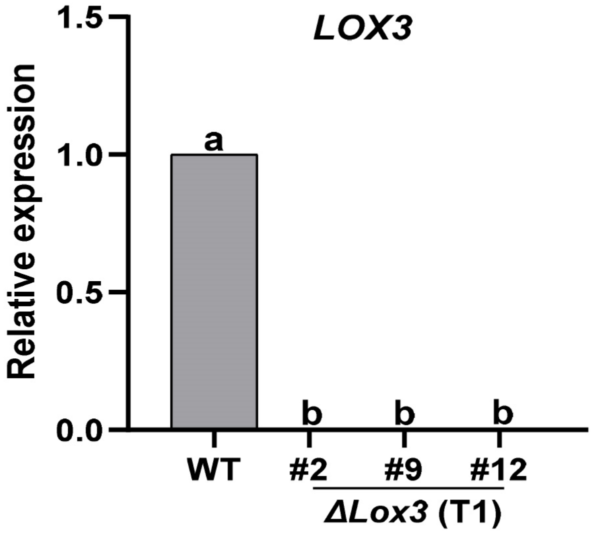

Figure S2. Expression of *LOX3* in T_1 generations of *LOX3* knockout transgenic rice lines and WT. The data are expressed as mean ± standard deviation (SD). Different lowercase letters above the columns indicate significant differences ($p < 0.05$).

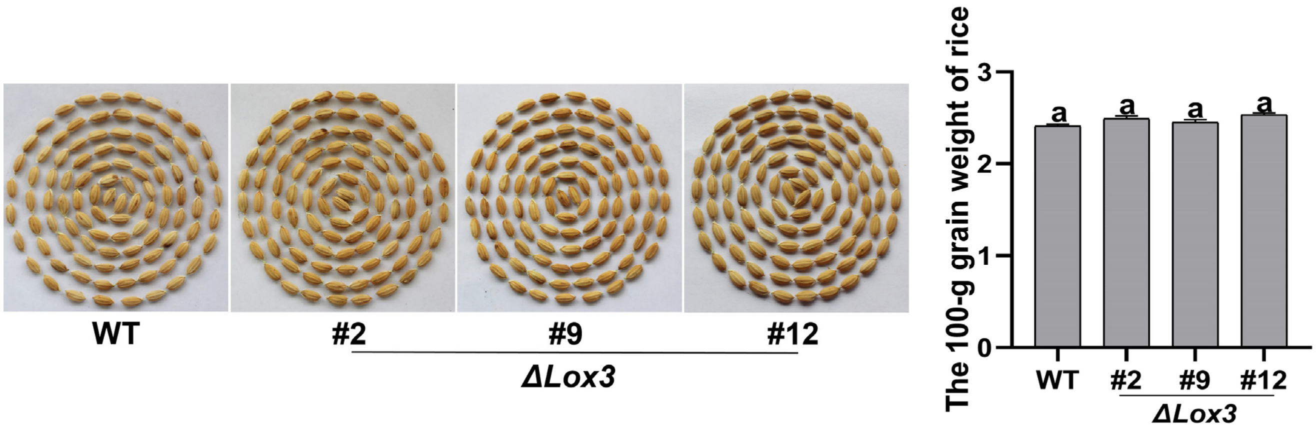

Figure S3. The 100-g grain weight of three *ΔLox3* transgenic rice lines and WT. The data are expressed as mean ± standard deviation (SD). Different lowercase letters above the columns indicate significant differences ($p < 0.05$).

Table S1. Primers used in this study.

Gene name	Primer sequence (5'–3')
Rice gene expression	
Ubiqutin	F: CTGTCAACTGCCGCAAGAAG<br>R: GGCGAGTGACGCTCTAGTTC
OsbHLH35	F: GTCATTTTTGCGACACCCCA<br>R: CCTCGCAAGAAAACAACCACC
OsAOS2	F: GGAGGAAGCTGCTGCAATAC<br>R: GGAGGTTGAAGCTTTGGTGA
OsWRKY45	F: AATCATGGATGGACACGGGC<br>R: AGCTGAGACGACACATCAACA
OsPR1a	F: TCGTATGCTATGCTA CGTGTTT<br>R: CACTA AGCAAATACGGCTGACA
OsLOX3	F: TCGCATTGGCTCGCACCCT<br>R: TCGTCTTCTTCAGCCGCACGAT
OsRbohB	F: AGCTGCACCCTTGCATCTTCTCTCT<br>R: ATCCGTAGGGCTTTGACATT
OsPOX1	F: TATGCGTTTCACTTGCTT<br>R: CTCCTACACTTTGCGTTT
M. oryzae gene expression	
MoActin	F: CCATGTACCCTGGTCTTTCG<br>R: TTCGAGATCCACATCTGCTG
MoBAS4	F: TGTGTCAGAACCTACCCCGA<br>R: CGCCAAGGTTAGGGCATTTC
MoCDIP1	F: CCTTACTTTGGGCTCCGGTT<br>R: CGCACGTTGAAGTTGTCTGG
Fungal relative growth	
q-gUBQ	F: TTCTGGTCCTTCCACTTTCAG<br>R: ACGATTGATTTAACCAGTCCATGA
q-gPot2	F: ACGACCCGTCTTTACTTATTTGG<br>R: AAGTAGCGTTGGTTTTGTTGGAT

Table S2. Specific DEGs in #2_vs_WT, *M. oryzae* infected #2_vs_*M. oryzae* infected WT, JA + *M. oryzae* infected #2_vs_JA + *M. oryzae* infected WT.

Specific DEGs in B1_vs_A1 comparing B1_vs_A1, B2_vs_A2 and B3_vs_A3			
**Gene ID**	**Up/down**	**Gene name**	**Description**
gene-psbA	DOWN	-	-
Os01g0130900	UP	-	Conserved hypothetical protein
Os01g0155800	UP	-	Conserved hypothetical protein
Os01g0162400	UP	-	Conserved hypothetical protein
Os01g0176700	UP	-	Myb-like DNA-binding domain, SHAQKYF class domain containing protein
Os01g0212400	UP	OsEFCAX1	EF-Hand type domain containing protein
Os01g0224100	DOWN	OsERF	-
Os01g0256500	UP	-	Similar to ZnI
Os01g0332150	DOWN	-	Hypothetical protein
Os01g0339500	DOWN	ONAC030	Similar to No apical meristem protein
Os01g0392600	UP	-	Conserved hypothetical protein
Os01g0537250	UP	-	Protein of unknown function DUF3778 domain containing protein
Os01g0566701	UP	OsbHLH117	Non-protein coding transcript
Os01g0580500	UP	ACO7	ACC oxidase, Ethylene biosynthesi
Os01g0587400	UP	-	Serine/threonine protein kinase-related domain containing protein
Os01g0606900	DOWN	OsDjC10	Heat shock protein DnaJ, N-terminal domain containing protein
Os01g0692400	UP	-	Conserved hypothetical protein
Os01g0701700	UP	-	SAM dependent carboxyl methyltransferase family protein
Os01g0707500	UP	OsbHLH118	Similar to Transcription factor LAX PANICLE
Os01g0723100	UP	-	Senescence-associated family protein
Os01g0740300	UP	-	Hypothetical protein
Os01g0740651	DOWN	-	Hypothetical protein
Os01g0756300	UP	-	Conserved hypothetical protein
Os01g0806200	UP	OsFbox045	Cyclin-like F-box domain containing protein
Os01g0823700	UP	-	Protein of unknown function DUF641, plant domain containing protein
Os01g0826000	UP	OsATX	Heavy metal transport/detoxification protein domain containing protein
Os01g0835500	DOWN	rbohE	Similar to Respiratory burst oxidase protein
Os01g0856500	UP	OsLAX1	Auxin transporter, Primary root and root hair elongation, Cd stress respons

Os01g0866200	UP	-	Similar to Histone H3
Os01g0871100	DOWN	-	Alpha/beta hydrolase fold-1 domain containing protein
Os01g0892800	UP	OsSTA40	Similar to Ankyrin-kinase
Os01g0910800	UP	-	Conserved hypothetical protein
Os01g0956200	UP	-	Glycosyltransferase AER61, uncharacterized domain containing protein
Os02g0100200	DOWN	-	Steroid nuclear receptor, ligand-binding domain containing protein
Os02g0129800	UP	-	Conserved hypothetical protein
Os02g0490500	UP	-	UDP-glucuronosyl/UDP-glucosyltransferase family protein
Os02g0535901	UP	OsFbox092	Cyclin-like F-box domain containing protein
Os02g0580900	UP	OsNRT1.3A	TGF-beta receptor, type I/II extracellular region family protein
Os02g0623400	UP	OsG1L3	Similar to G1-like protein
Os02g0671800	UP	-	Integrase, catalytic core domain containing protein
Os02g0673500	UP	-	Basic helix-loop-helix dimerisation region bHLH domain containing protein
Os02g0684500	UP	-	Similar to Histone H4
Os02g0689900	UP	OsNRT1.5A	TGF-beta receptor, type I/II extracellular region family protein
Os02g0696900	UP	OsKANADI1	Myb-like DNA-binding domain, SHAQKYF class domain containing protein
Os02g0702300	UP	-	Conserved hypothetical protein
Os02g0716500	UP	OsFAD2	Delta12-fatty acid desaturase
Os02g0720500	UP	-	Peptidase A1 domain containing protein
Os02g0755400	UP	-	Similar to RNA recognition motif-containing protein SEB-4
Os02g0772100	UP	-	Conserved hypothetical protein
Os02g0787300	UP	SMG1	Mitogen-activated protein kinase kinase 4, Defense response, Cell proliferation, Grain growt
Os02g0787600	UP	OsGLR1.2	Glutamate receptor-related domain containing protein
Os02g0814000	DOWN	-	Protein of unknown function DUF862, eukaryotic domain containing protein
Os03g0116400	UP	OsRhmbd7	Similar to Membrane protein
Os03g0149300	UP	-	Protein of unknown function DUF6, transmembrane domain containing protein
Os03g0152000	DOWN	-	Heavy metal transport/detoxification protein domain containing protein
Os03g0170900	UP	OsSUT1	Sucrose transporter
Os03g0183200	UP	OsERF	-
Os03g0195300	UP	OsSultr2	Similar to Low affinity sulphate transporter 3
Os03g0200200	UP	-	Conserved hypothetical protein
Os03g0223301	UP	-	Hypothetical gene

Os03g0230300	UP	OsSRO1c	Homologue of SRO (similar to RCD one), Regulation of stomatal closure, Abiotic stress respons
Os03g0261800	UP	-	Protein of unknown function DUF3049 domain containing protein
Os03g0282232	UP	-	Conserved hypothetical protein
Os03g0299800	UP	-	Protein of unknown function Cys-rich family protein
Os03g0304500	UP	-	Tetratricopeptide-like helical domain containing protein
Os03g0321300	UP	OsFbox137	Cyclin-like F-box domain containing protein
Os03g0371000	UP	OsSTA101	Similar to Cytochrome P450 family protein, expressed
Os03g0375833	UP	-	Non-protein coding transcript
Os03g0389100	UP	OsMC8	Peptidase C14, caspase catalytic domain containing protein
Os03g0426350	UP	-	Conserved hypothetical protein
Os03g0561283	UP	-	Conserved hypothetical protein
Os03g0613100	UP	-	Conserved hypothetical protein
Os03g0624300	UP	-	Conserved hypothetical protein
Os03g0668500	UP	SIP31	Conserved hypothetical protein
Os03g0669300	UP	-	Beta-1,3-glucanase-like protein
Os03g0685300	UP	-	Glutamine amidotransferase class-I domain containing protein
Os03g0711150	DOWN	-	Hypothetical protein
Os03g0758000	UP	OsWRKY3	Similar to WRKY23 transcription factor (Fragment)
Os03g0787300	DOWN	OsDjA5	Similar to DnaJ homolog
Os03g0799000	UP	-	Similar to Histone H1
Os03g0826900	UP	OsEnS-58	Conserved hypothetical protein
Os04g0128900	UP	YUCCA7	Flavin monooxygenase-like enzyme, Auxin biosynthesi
Os04g0129900	UP	-	Similar to OSIGBa0103I21.1 protein
Os04g0200700	UP	-	Hypothetical protein
Os04g0249500	UP	SUS7	Similar to H0211A12.6 protein
Os04g0310400	UP	OsWAK36	Similar to H0211A12.15 protein
Os04g0389901	DOWN	-	Hypothetical gene
Os04g0397800	UP	-	Conserved hypothetical protein
Os04g0398000	UP	OsERF101	ERF transcription factor, A member of APETALA2/Ethylene-Responsive Element Binding Protein(AP2/EREBP) family, Regulation of drought stress response, Innate immunit
Os04g0429600	UP	OsCslH3	Putative cellulose synthase-like protein H3
Os04g0430600	UP	-	Similar to OSIGBa0160I14.1 protein
Os04g0431700	UP	Bph6	Exocyst-localized protein, Resistance to planthoppers, (Nipponbare: BPH-susceptible
Os04g0475600	UP	-	2OG-Fe(II) oxygenase domain containing protein
Os04g0493600	UP	-	Similar to Lectin-C precursor (PL-C)

Os04g0498900	UP	-	Similar to N-acetyltransferase ESCO1
Os04g0514400	DOWN	-	Peptidoglycan binding-like domain containing protein
Os04g0531900	UP	-	Short-chain dehydrogenase/reductase SDR domain containing protein
Os04g0580800	UP	OsRFPH2-6	Zinc finger, RING/FYVE/PHD-type domain containing protein
Os04g0612301	DOWN	-	Hypothetical protein
Os04g0684600	UP	-	Similar to H0913C04.5 protein
Os04g0688300	UP	prx61	Haem peroxidase, plant/fungal/bacterial family protein
Os04g0689700	UP	-	RNA recognition motif, RNP-1 domain containing protein
Os05g0114700	UP	-	Myb-type HTH DNA-binding domain domain containing protein
Os05g0142000	UP	-	Conserved hypothetical protein
Os05g0186900	UP	OsIAA16	Similar to Auxin-responsive protein IAA16
Os05g0209500	UP	-	Conserved hypothetical protein
Os05g0269500	UP	-	Generative cell specific-1, HAP2-GCS1 domain containing protein
Os05g0277350	DOWN	-	Similar to Leucine Rich Repeat family protein, expressed
Os05g0361500	UP	OsPME17	Similar to Pectinesterase
Os05g0380900	DOWN	OsCML15	Similar to Polcalcin Jun o 2 (Calcium-binding pollen allergen Jun o 2)
Os05g0401500	UP	-	Similar to oxidoreductase, 2OG-Fe oxygenase family protein
Os05g0426000	UP	OsSWEET1b	MtN3 and saliva related transmembrane protein family protein
Os05g0447200	UP	OsLAX3	Similar to auxin transporter-like protein 1
Os05g0461600	UP	-	Similar to SIK1 protein (Nucleolar protein NOP56)
Os05g0465000	UP	-	Conserved hypothetical protein
Os05g0468800	UP	-	Phosphatidylethanolamine-binding protein PEBP domain containing protein
Os05g0503650	DOWN	-	Non-protein coding transcript
Os05g0506000	UP	-	Similar to MS5-like protein (Fragment)
Os05g0590201	DOWN	-	Hypothetical gene
Os06g0118700	UP	-	Peptidase A1 domain containing protein
Os06g0130900	UP	-	Similar to Histone H3 (Fragment)
Os06g0226700	UP	OsTCP19	Similar to PCF1
Os06g0294000	UP	-	Conserved hypothetical protein
Os06g0317200	UP	-	Similar to glycine-rich cell wall structural protein
Os06g0559600	UP	-	Hypothetical conserved gene
Os06g0604200	UP	OsPLDalpha4	Chloroplast-localized phospholipase D, Herbivore defens
Os06g0667000	UP	-	Serine/threonine protein kinase domain containing protein
Os06g0672400	UP	OsG1L2	Protein of unknown function DUF640 domain containing protein

Os07g0111400	UP	OsWRKY29	WRKY transcription factor 29
Os07g0147550	UP	OsPsbR2	Similar to Photosystem II 10 kDa polypeptide, chloroplast
Os07g0178800	DOWN	-	Similar to Low molecular mass early light-inducible protein HV90, chloroplast precursor (ELIP)
Os07g0190800	UP	OsTRXh5	Similar to Thioredoxin h
Os07g0442800	UP	-	Conserved hypothetical protein
Os07g0480800	UP	OsXTH21	Cellulase (EC 3.2.1.4)
Os07g0509800	UP	OsAPRL1	Similar to adenosine 5'-phosphosulfate reductase 2
Os07g0542400	UP	-	Similar to Receptor protein kinase
Os07g0543100	DOWN	-	Similar to Beta-amylase (EC 3.2.1.2)
Os07g0566500	UP	ONAC010	Similar to NAC domain protein
Os07g0584100	UP	OsWNK5	Similar to MAP kinase-like protein
Os07g0605200	DOWN	MADS18	APETALA1 (AP1)/ FRUITFULL (FUL)-like MADS box transcription factor, Specification of inflorescence meristem identit
Os07g0624100	DOWN	-	Similar to cDNA clone:J033024N05, full insert sequence
Os07g0631200	DOWN	-	Zinc finger, RING/FYVE/PHD-type domain containing protein
Os07g0664600	UP	RMP8	Glucose/ribitol dehydrogenase family protein
Os08g0108100	UP	-	Pectinesterase inhibitor domain containing protein
Os08g0113000	UP	prx117	Similar to Peroxidase 47 precursor (EC 1.11.1.7) (Atperox P47) (ATP32)
Os08g0139700	DOWN	OsTPS29	Similar to terpene synthase 6
Os08g0158600	UP	-	Similar to Cell wall invertase (EC 3.2.1.26)
Os08g0246800	UP	-	Conserved hypothetical protein
Os08g0389601	UP	-	Non-protein coding transcript
Os08g0403300	UP	-	Heavy metal transport/detoxification protein domain containing protein
Os08g0525000	UP	OsRab11G2	Ras GTPase family protein
Os08g0540400	UP	OsCDPK21	Similar to Calcium-dependent protein kinase
Os08g0546800	DOWN	HSFB2B	Similar to Heat stress transcription factor B-2b
Os09g0109600	UP	OsDSSR1	Small peptide, Drought toleranc
Os09g0330700	UP	-	Ankyrin repeat domain containing protein
Os09g0356800	DOWN	-	Protein kinase, core domain containing protein
Os09g0403000	UP	-	Hypothetical conserved gene
Os09g0408550	UP	-	Heavy metal-associated domain, HMA domain containing protein
Os09g0412400	UP	-	Conserved hypothetical protein
Os09g0433600	UP	-	Similar to Histone H4
Os09g0455300	UP	OsbHLH120	Similar to INDEHISCENT protein

Os09g0457900	DOWN	ERF102	AP2/ERF transcription factor, Regulation of the internode elongatio
Os09g0494600	UP	-	Protein of unknown function DUF599 family protein
Os09g0559600	UP	-	Thioredoxin domain 2 containing protein
Os10g0104000	UP	-	Hypothetical conserved gene
Os10g0109600	UP	poxA	Peroxidase (EC 1.11.1.7)
Os10g0154700	UP	OsCYP18-4	Similar to Cyclophilin Dicyp-2
Os10g0210500	UP	OsUMAMIT15	Protein of unknown function DUF6, transmembrane domain containing protein
Os10g0409400	UP	RD22	beta subunit of polygalacturonase 1, Abiotic stress respons
Os10g0478000	UP	G1L5	Transcriptional regulator, Regulator of meristem activity, Regulation of inflorescence developmen
Os10g0509401	UP	-	Conserved hypothetical protein
Os10g0516900	UP	-	Conserved hypothetical protein
Os10g0551200	UP	-	Similar to Scl1 protein (Fragment)
Os10g0567900	UP	-	Similar to F-box protein interaction domain containing protein, expressed
Os10g0576600	UP	-	Tetratricopeptide-like helical domain containing protein
Os11g0133001	UP	-	Similar to Ser/Thr protein kinase (Fragment)
Os11g0133100	UP	-	Similar to D-mannose binding lectin family protein
Os11g0149200	UP	-	Conserved hypothetical protein
Os11g0170900	UP	OsBURP17	Similar to BURP domain-containing protein 17
Os11g0177400	UP	OsABCG27	Similar to ATPase, coupled to transmembrane movement of substances
Os11g0213700	UP	-	Leucine-rich repeat, typical subtype containing protein
Os11g0222900	UP	-	NB-ARC domain containing protein
Os11g0226201	DOWN	-	Similar to Protein kinase domain containing protein, expressed
Os11g0246250	UP	-	Non-protein coding transcript
Os11g0289700	UP	-	Cytochrome P450 family protein
Os11g0417800	UP	-	Non-protein coding transcript
Os11g0470500	UP	-	Similar to Protein kinase domain containing protein, expressed
Os11g0471300	DOWN	-	Similar to OSIGBa0145N07.3 protein
Os11g0543100	UP	-	Conserved hypothetical protein
Os11g0677101	UP	-	Similar to NB-ARC domain containing protein
Os11g0685500	DOWN	-	Hypothetical protein
Os11g0701500	UP	-	Similar to Class III chitinase homologu
Os12g0199000	UP	-	Similar to Jasmonate-induced protein
Os12g0210500	UP	-	Conserved hypothetical protein
Os12g0476200	UP	SWEET13	Sucrose transporter, TAL effector PthXo2-dependent disease susceptibility to bacterial pathoge

Os12g0516000	UP	-	Conserved hypothetical protein
Os12g0552400	UP	-	Rossmann-like alpha/beta/alpha sandwich fold domain containing protein
Os12g0554100	UP	OsTBL34	Conserved hypothetical protein
Os12g0567300	UP	R2R3-MYB	Myb transcription factor domain containing protein
Os12g0569700	DOWN	OsHsp23.7	Similar to Heat shock protein 70
Os12g0595800	UP	-	Similar to cDNA clone:J023098M23, full insert sequence
Os12g0630200	UP	-	Thaumatin, pathogenesis-related family protein
**Specific DEGs in B2_vs_A2 comparing B2_vs_A2, B1_vs_A1 and B3_vs_A3**			
**Gene ID**	**up/down**	**Gene name**	**Description**
Os01g0106400	UP	-	Similar to Isoflavone reductase homolog IRL (EC 1.3.1.-)
Os01g0111800	UP	-	Conserved hypothetical protein
Os01g0111900	UP	SIP34	Glutelin family protein
Os01g0117200	DOWN	OsRLCK16	Similar to ARK protein (Fragment)
Os01g0140400	UP	PLRRP	Similar to leucine-rich repeat protein-related
Os01g0174100	DOWN	-	Conserved hypothetical protein
Os01g0194000	UP	-	Conserved hypothetical protein
Os01g0195400	UP	-	Harpin-induced 1 domain containing protein
Os01g0210400	UP	-	Protein of unknown function DUF616 domain containing protein
Os01g0214600	UP	OsGELP2	Lipase, GDSL domain containing protein
Os01g0248701	UP	-	Terpenoid synthase domain containing protein
Os01g0249200	UP	OsGLP1-1	Similar to Nectarin 1 precursor (EC 1.15.1.1) (Superoxide dismutase [Mn])
Os01g0281600	UP	OsENODL4	Cupredoxin domain containing protein
Os01g0329900	UP	OsGELP14	Similar to Lipase homolog (Fragment)
Os01g0330100	UP	OsGELP15	Similar to Alpha-L-fucosidase 2
Os01g0343300	UP	OsGATA8	Zinc finger, NHR/GATA-type domain containing protein
Os01g0508000	UP	Os1bglu1	Similar to Beta-glucosidase
Os01g0508100	UP	RH3	Ferritin/ribonucleotide reductase-like family protein
Os01g0508500	UP	RH2	Conserved hypothetical protein
Os01g0511800	UP	-	Conserved hypothetical protein
Os01g0525700	UP	-	Similar to DVL4/RTFL17 (ROTUNDIFOLIA LIKE 17)
Os01g0545100	UP	OsMYB86-L1	Conserved hypothetical protein
Os01g0555200	DOWN	-	Asp/Glu racemase family protein
Os01g0601200	UP	OsRLCK41	Similar to Protein kinase family protein
Os01g0603300	UP	-	Similar to MCB2 protein
Os01g0609900	UP	OsPDR8	Similar to Pleiotropic drug resistance protein 4

Os01g0618900	UP	OsPGL31	Similar to Polygalacturonase
Os01g0619800	DOWN	-	Similar to Hydrolase, hydrolyzing O-glycosyl compounds
Os01g0619900	DOWN	OsTCL2	Similar to DNA binding
Os01g0625200	UP	-	Similar to kinesin motor family protein
Os01g0652800	UP	OsTBL57	Protein of unknown function DUF231, plant domain containing protein
Os01g0656400	UP	OsWRKY15	Similar to WRKY transcription factor 15
Os01g0659900	UP	OsFBK1	Kelch-domain-containing F-box protein, Component of an SCF E3 ligase, Regulation of anther and root secondary cell wall thickenings, Regulation of lignificatio
Os01g0660300	UP	-	Similar to Pyruvate kinase
Os01g0670500	UP	-	Conserved hypothetical protein
Os01g0681700	DOWN	-	Hypothetical conserved gene
Os01g0690600	UP	-	Conserved hypothetical protein
Os01g0695800	UP	MDR4	Similar to MDR-like ABC transporter
Os01g0697100	UP	-	UDP-glucuronosyl/UDP-glucosyltransferase family protein
Os01g0702700	UP	OsMYB14	Similar to Transcription factor MYB86 (Myb-related protein 86) (AtMYB86) (Myb homolog 4) (AtMyb4)
Os01g0714800	UP	OsWRKY26	WRKY transcription factor 26
Os01g0716200	UP	-	IQ calmodulin-binding region domain containing protein
Os01g0717000	UP	-	Similar to GmCK1p (EC 2.7.1.32)
Os01g0723600	UP	-	Ribose-phosphate pyrophosphokinase 3 (EC 2.7.6.1) (Phosphoribosyl pyrophosphate synthetase 3)
Os01g0738300	UP	-	Protein kinase, core domain containing protein
Os01g0744000	UP	-	Similar to Kinesin heavy chain (Fragment)
Os01g0750000	UP	OsRab11D2	Similar to ras-related protein RIC2
Os01g0787000	UP	PRX19	Similar to Peroxidase (EC 1.11.1.7)
Os01g0796000	UP	-	Conserved hypothetical protein
Os01g0805900	UP	OsTUB4	Tubulin beta-2 chain (Beta-2 tubulin)
Os01g0813700	UP	Os1bglu2	Similar to Non-cyanogenic beta-glucosidase
Os01g0816100	UP	OsNAC4	Similar to NAC domain protein
Os01g0831250	UP	-	Hypothetical gene
Os01g0834900	UP	-	Hypothetical conserved gene
Os01g0842500	UP	OsLAC5	Similar to Laccase (EC 1.10.3.2)
Os01g0863300	UP	-	Similar to MCB2 protein
Os01g0868900	UP	OsSub11	Peptidase S8, subtilisin-related domain containing protein
Os01g0872566	UP	-	Hypothetical protein
Os01g0878900	UP	-	Similar to 4,5-DOPA dioxygenase extradiol-like protein
Os01g0879200	UP	DRR206	Plant disease resistance response protein domain containing protein

Os01g0882800	UP	OsAAP11E	Similar to Amino acid carrier (Fragment)
Os01g0883800	DOWN	20ox2	Similar to GA C20oxidase2
Os01g0896200	UP	-	Similar to SF16 protein
Os01g0897700	UP	OsFH1	Similar to FH protein NFH1
Os01g0908600	UP	OsProT1	Amino acid transporter, transmembrane domain containing protein
Os01g0914400	UP	STOMAGEN	Similar to EPIDERMAL PATTERNING FACTOR-like protein 9
Os01g0926600	UP	OsGT47A	Similar to Pectin-glucuronyltransferase
Os01g0926700	UP	OsIRX10	Similar to secondary cell wall-related glycosyltransferase family 47
Os01g0942300	UP	OsEGL2	Similar to Beta glucanase precursor (EC 3.2.1.73) (Fragment)
Os01g0951100	UP	-	Arf GTPase activating protein family protein
Os01g0952000	UP	GER8	Similar to Germin-like protein 1-3
Os01g0971400	UP	-	Similar to Cysteine proteinase
Os02g0125400	UP	OsCYCP4	Negative regulatory factor PREG domain containing protein
Os02g0151300	UP	OsGH9B7	Similar to Cellulase (Fragment)
Os02g0199600	DOWN	-	Similar to F-box domain containing protein
Os02g0275200	UP	-	Xyloglucan fucosyltransferase family protein
Os02g0303500	UP	-	Eggshell protein family protein
Os02g0322400	UP	-	Similar to NPKL2 (Fragment)
Os02g0327500	UP	-	Protein of unknown function DUF266, plant family protein
Os02g0329800	UP	XAX1	Glycosyltransferase, Xylan biosynthesi
Os02g0465600	UP	-	Kelch-type beta propeller domain containing protein
Os02g0526000	UP	-	Similar to H0502B11.6 protein
Os02g0538700	UP	-	Harpin-induced 1 domain containing protein
Os02g0549200	UP	OsRLCK73	Similar to Ser Thr specific protein kinase-like protein
Os02g0549401	UP	-	Hypothetical gene
Os02g0569400	UP	CYP76M8	Cytochrome P450 family protein
Os02g0589900	UP	-	Hypothetical conserved gene
Os02g0597300	UP	-	Leucine-rich repeat domain containing protein
Os02g0602900	DOWN	-	Conserved hypothetical protein
Os02g0618400	UP	OsMPS	R2R3-type MYB transcription factor, Adaptive growth regulatio
Os02g0620400	UP	-	RmlC-like jelly roll fold domain containing protein
Os02g0625300	UP	OsPPCK2	Similar to PEPC kinase
Os02g0626400	UP	OsPAL	Phenylalanine ammonia-lyase (EC 4.3.1.5)
Os02g0627100	UP	OsPAL1	Similar to Phenylalanine ammonia-lyase (EC 4.3.1.5)
Os02g0632800	UP	WAK14	Wall-associated kinase, Positive regulation of rice blast resistanc
Os02g0638650	UP	OsERF	-
Os02g0639300	UP	OsCBSX11	Conserved hypothetical protein
Os02g0640300	UP	-	Similar to Membrane steroid binding protein 1 (AtMP1)

Os02g0654300	UP	-	Similar to Protein kinase KIPK
Os02g0655800	UP	-	Serine/threonine protein kinase domain containing protein
Os02g0657000	UP	OsERF	-
Os02g0668500	DOWN	-	Cellular retinaldehyde-binding/triple function, C-terminal domain containing protein
Os02g0675700	UP	-	Protein of unknown function DUF248, methyltransferase putative family protein
Os02g0676800	UP	OsERF	-
Os02g0677300	UP	OsDREB1G	Similar to CRT/DRE binding factor 1
Os02g0684400	DOWN	-	Similar to OXS
Os02g0712700	UP	-	Concanavalin A-like lectin/glucanase domain containing protein
Os02g0715300	UP	-	Conserved hypothetical protein
Os02g0718100	UP	OsAGP22	Non-protein coding transcript
Os02g0718600	UP	OsAGP21	Conserved hypothetical protein
Os02g0719600	UP	JMT-2	SAM dependent carboxyl methyltransferase family protein
Os02g0720600	UP	-	Peptidase A1 domain containing protein
Os02g0738200	UP	OsPUB40	Zinc finger, RING/FYVE/PHD-type domain containing protein
Os02g0738300	UP	-	Conserved hypothetical protein
Os02g0755000	UP	OsTSD2	Putative methyltransferase, Pectin synthesis and methylesterification, Root development, Cellular adhesio
Os02g0768400	UP	-	Hypothetical gene
Os02g0771400	UP	OsPFA-DSP2	Protein-tyrosine phosphatase, SIW14-like domain containing protein
Os02g0771700	UP	Gns9	Glycoside hydrolase, family 17 protein
Os02g0775600	UP	-	Zinc finger, C2H2-like domain containing protein
Os02g0781400	UP	-	Similar to 20 kDa chaperonin, chloroplast precursor (Protein Cpn21) (Chloroplast protein Cpn10) (Chloroplast chaperonin 10) (Ch-CPN10)
Os02g0803300	UP	-	Epsin-like, N-terminal domain containing protein
Os02g0829000	UP	-	Hypothetical gene
Os03g0113000	UP	OsRLCK95	Serine/threonine protein kinase domain containing protein
Os03g0116300	UP	-	Similar to catalytic/ hydrolase
Os03g0118800	UP	-	Similar to Hydroxymethylglutaryl-CoA synthase
Os03g0123800	UP	-	Similar to Hydrolase
Os03g0126300	UP	-	Similar to cDNA clone:001-042-G03, full insert sequence
Os03g0130100	UP	-	Similar to Acyl-activating enzyme 11
Os03g0160600	DOWN	-	Similar to type I inositol-1,4,5-trisphosphate 5-phosphatase CVP2
Os03g0162900	UP	-	Pentatricopeptide repeat domain containing protein
Os03g0183500	UP	-	Protein of unknown function DUF581 family protein

Os03g0203700	UP	osACA1	Similar to Calcium-transporting ATPase 2, plasma membrane-type (EC 3.6.3.8) (Ca(2+)-ATPase isoform 2)
Os03g0232800	UP	-	Similar to Lecithin:cholesterol acyltransferase family protein, expressed
Os03g0234900	UP	PRX39	Similar to Peroxidase (EC 1.11.1.7)
Os03g0246500	UP	-	Protein of unknown function DUF869, plant family protein
Os03g0258200	UP	-	Alpha/beta hydrolase fold-3 domain containing protein
Os03g0262700	UP	-	Harpin-induced 1 domain containing protein
Os03g0264400	UP	OASA2	Anthranilate synthase alpha 2 subunit
Os03g0272650	DOWN	-	Non-protein coding transcript
Os03g0280400	DOWN	-	Similar to plant-specific domain TIGR01589 family protein
Os03g0285200	UP	-	Conserved hypothetical protein
Os03g0285300	UP	-	Conserved hypothetical protein
Os03g0289800	UP	-	Similar to Leucoanthocyanidin dioxygenase-like protein
Os03g0300900	UP	-	Glycosyl transferase, family 8 protein
Os03g0301800	UP	-	Similar to Kinesin-like polypeptides 9 (Fragment)
Os03g0304800	UP	-	Lg106-like family protein
Os03g0305400	UP	-	UspA domain containing protein
Os03g0316200	UP	OsGolS1	Similar to Galactinol synthase (Fragment)
Os03g0323600	DOWN	OsDjC29	Similar to DnaJ domain containing protein, expressed
Os03g0341600	DOWN	-	Pollen Ole e 1 allergen/extensin domain containing protein
Os03g0342100	DOWN	-	Pollen Ole e 1 allergen/extensin domain containing protein
Os03g0344400	UP	-	Hypothetical conserved gene
Os03g0368900	UP	PRX45	Haem peroxidase family protein
Os03g0386500	UP	OsTPP9	Similar to Trehalose-6-phosphate phosphatase
Os03g0389700	UP	OsDAHPS 1	3-Deoxy-D-arabino-heptulosonate 7-phosphate synthase, Chorismate biosyn-thesi
Os03g0402800	UP	OsJAZ5	Tify domain containing protein
Os03g0404800	UP	-	NAD(P)-binding domain containing protein
Os03g0405500	UP	-	Similar to PDI-like protein
Os03g0412300	UP	-	Heavy metal transport/detoxification protein domain containing protein
Os03g0413100	UP	OsACR7	Similar to ACR4
Os03g0416200	UP	BC1	BRITTLE CULM1
Os03g0431600	DOWN	-	Hypothetical conserved gene
Os03g0567100	UP	-	Conserved hypothetical protein
Os03g0620400	DOWN	-	Hypothetical conserved gene
Os03g0629800	UP	-	Conserved hypothetical protein
Os03g0646100	UP	FtsZ2-1	Similar to Plastid division protein ftsZ1 precursor
Os03g0658800	UP	Os-CYP87C2	Cytochrome P450 family protein
Os03g0703100	UP	Os3bglu8	Similar to Beta-glucosidase

Os03g0713600	UP	-	Aldo/keto reductase domain containing protein
Os03g0722500	UP	-	Glycoside hydrolase, family 17 protein
Os03g0730500	UP	OsSCP20	Peptidase S10, serine carboxypeptidase family protein
Os03g0739500	UP	-	Conserved hypothetical protein
Os03g0740833	UP	-	Conserved hypothetical protein
Os03g0751600	UP	-	Similar to Circumsporozoite protein
Os03g0757900	UP	-	Similar to UDP-glucose 6-dehydrogenase (EC 1.1.1.22) (UDP-Glc dehydrogenase) (UDP-GlcDH) (UDPGDH)
Os03g0761000	UP	-	SWIB/MDM2 domain containing protein
Os03g0788200	UP	-	MD-2-related lipid-recognition domain containing protein
Os03g0790500	UP	GID1	Alpha/beta hydrolase fold-3 domain containing protein
Os03g0793800	UP	OsLTPG7	Plant lipid transfer protein and hydrophobic protein, helical domain containing protein
Os03g0802500	UP	-	ATPase, AAA-type, core domain containing protein
Os03g0806800	UP	-	Conserved hypothetical protein
Os03g0815200	UP	-	Similar to Methylenetetrahydrofolate reductase (EC 1.5.1.20)
Os03g0817800	UP	OsTBL6	Protein of unknown function DUF231 domain containing protein
Os03g0831700	UP	-	Conserved hypothetical protein
Os03g0837100	UP	OsCesA5	Similar to Cellulose synthase-6
Os03g0852400	UP	OsAGP31	Conserved hypothetical protein
Os03g0859900	UP	-	Protein of unknown function DUF547 domain containing protein
Os04g0103700	DOWN	-	Serine/threonine protein kinase-related domain containing protein
Os04g0116600	UP	-	Glucose/ribitol dehydrogenase family protein
Os04g0142400	UP	-	Conserved hypothetical protein
Os04g0154800	UP	-	Protein of unknown function DUF594 family protein
Os04g0168300	UP	-	Conserved hypothetical protein
Os04g0203450	UP	-	Hypothetical protein
Os04g0243700	UP	OsCTR1	Zinc finger, RING/FYVE/PHD-type domain containing protein
Os04g0271450	DOWN	-	Non-protein coding transcript
Os04g0282400	DOWN	-	Similar to FPF1 protein-like (RAA1)
Os04g0344100	UP	-	Similar to OSIGBa0106G08.3 protein
Os04g0383700	UP	OsRALF-16	Conserved hypothetical protein
Os04g0387000	DOWN	-	Hypothetical conserved gene
Os04g0396500	UP	LAX2	Regulation of axillary meristem formatio
Os04g0399300	DOWN	-	Similar to Nudix hydrolase 13, mitochondrial precursor (EC 3.6.1.-) (AtNUDT13)
Os04g0401000	UP	PI21	Proline-rich protein, Blast resistanc
Os04g0401100	UP	-	Similar to OSIGBa0113L04.8 protein
Os04g0406600	UP	-	Prephenate dehydratase domain containing protein
Os04g0412900	UP	-	Zinc finger, Sec23/Sec24-type domain containing protein

Os04g0416700	UP	-	Harpin-induced 1 domain containing protein
Os04g0439600	UP	OsCRR1	Regulator of chromosome condensation/beta-lactamase-inhibitor protein II domain containing protein
Os04g0447100	UP	SPH	Similar to Lipoxygenase
Os04g0482300	UP	-	Kelch related domain containing protein
Os04g0497000	UP	Os-NADPH1	Similar to Allyl alcohol dehydrogenase
Os04g0499200	UP	-	Enhancer of rudimentary family protein
Os04g0514800	UP	-	Similar to Dual specificity kinase 1
Os04g0538400	UP	-	Similar to Nodulin 21 (N-21)
Os04g0550200	UP	OsERF	-
Os04g0554200	UP	-	Similar to OSIGBa0143N19.13 protein
Os04g0567800	UP	OsDof18	Similar to OSIGBa0103M18.3 protein
Os04g0569900	UP	-	IQ calmodulin-binding region domain containing protein
Os04g0577800	UP	-	Uncharacterised protein family UPF0136, Transmembrane domain containing protein
Os04g0578600	DOWN	OsFRO2	Similar to H0404F02.15 protein
Os04g0583200	DOWN	-	Conserved hypothetical protein
Os04g0589400	UP	-	Conserved hypothetical protein
Os04g0593400	UP	-	Similar to OSIGBa0142I02-OSIGBa0101B20.20 protein
Os04g0597600	UP	OsPTR5	TGF-beta receptor, type I/II extracellular region family protein
Os04g0600900	UP	GRF3	Similar to B0403H10-OSIGBa0105A11.9 protein
Os04g0606000	UP	-	Transferase family protein
Os04g0634800	DOWN	-	Similar to H0315F07.8 protein
Os04g0640700	UP	-	Similar to Alpha-L-arabinofuranosidase/beta-D-xylosidase isoenzyme ARA-I
Os04g0654500	UP	-	Conserved hypothetical protein
Os04g0671200	UP	PAO4	Polyamine oxidase, Seed germinatio
Os04g0677700	UP	OsKTN80b	Katanin P80 ortholog, Katanin regulatory subunit P80
Os04g0685900	UP	OsRLCK174	Similar to Receptor-like protein kinase-like protein (Fragment)
Os04g0689500	UP	-	Similar to H0814G11.12 protein
Os04g0690100	UP	-	Zinc finger, C2H2-type domain containing protein
Os05g0103000	UP	OsbHLH083	Similar to MYC1
Os05g0114000	UP	-	Similar to PRLI-interacting factor F (Fragment)
Os05g0119850	UP	-	Hypothetical protein
Os05g0130300	UP	-	Conserved hypothetical protein
Os05g0130600	UP	-	Conserved hypothetical protein
Os05g0159300	UP	OsGELP62	Similar to Alpha-L-fucosidase 2
Os05g0163300	UP	OsFLA9	Similar to Arabinogalactan protein-like

Os05g0176100	UP	OsCesA1	Similar to Cellulose synthase BoCesA1
Os05g0189300	UP	-	Vegetative storage protein/acid phosphatase domain containing protein
Os05g0190300	UP	-	Acid phosphatase (Class B) family protein
Os05g0190500	UP	OsVSP2	Similar to Acid phosphatase
Os05g0191500	UP	-	Similar to stem 28 kDa glycoprotein
Os05g0199100	UP	-	Similar to NDR1/HIN1-Like protein 2
Os05g0210600	UP	-	Molecular chaperone, heat shock protein, Hsp40, DnaJ domain containing protein
Os05g0211100	UP	CYP51G3	Similar to Cytochrome P450-like protein
Os05g0273800	UP	-	Similar to Soluble epoxide hydrolase
Os05g0320700	UP	C4H2	Similar to Cytochrome P450
Os05g0350900	UP	-	Hypothetical conserved gene
Os05g0353500	UP	-	Similar to ATP binding protein
Os05g0361700	UP	OsERF	-
Os05g0368000	UP	RH1	Non-protein coding transcript
Os05g0386700	UP	-	Zinc finger, C2H2-type domain containing protein
Os05g0402900	UP	OsBphi262	Similar to EDGP
Os05g0406300	UP	-	Hypothetical protein
Os05g0410200	UP	Hsr203j	Alpha/beta hydrolase fold-3 domain containing protein
Os05g0427900	UP	-	Similar to DnaJ-like protein
Os05g0428100	UP	OsBgal6	Similar to Beta-galactosidase
Os05g0456300	UP	AKR4C15	Aldose reductase
Os05g0462000	DOWN	-	Similar to plant-specific domain TIGR01589 family protein
Os05g0462500	UP	-	Transmembrane receptor, eukaryota domain containing protein
Os05g0480400	UP	-	Serine/threonine protein kinase domain containing protein
Os05g0497200	UP	ERF130	AP2/ERF (APETALA2/ethylene-responsive factor) protein, Regulation of spikelet meristem determinancy and floral organ identity
Os05g0499800	UP	UGT705A3	UDP-glucuronosyl/UDP-glucosyltransferase family protein
Os05g0501001	UP	-	Hypothetical gene
Os05g0514400	UP	-	Hypothetical conserved gene
Os05g0522600	UP	-	Leucine-rich repeat, plant specific containing protein
Os05g0535900	UP	-	IQ calmodulin-binding region domain containing protein
Os05g0550800	UP	-	Serine/threonine protein kinase domain containing protein
Os05g0566400	UP	OsMPK7	Similar to Blast and wounding induced mitogen-activated protein kinase
Os05g0576600	UP	-	Conserved hypothetical protein
Os05g0585400	UP	-	Similar to Protein ABIL1
Os06g0109300	UP	-	Protein of unknown function DUF6, transmembrane domain containing protein
Os06g0117101	UP	-	Hypothetical gene
Os06g0123000	UP	-	Conserved hypothetical protein

Os06g0127100	UP	OsDREB1C	Dehydration-responsive element-binding protein 1C
Os06g0131700	UP	ONAC007	Similar to NAM protein
Os06g0143400	UP	FATB1	Similar to Acyl-ACP thioesterase (Fragment)
Os06g0147100	DOWN	-	Similar to cDNA clone:J023088C01, full insert sequence
Os06g0163300	UP	-	Harpin-induced 1 domain containing protein
Os06g0203200	UP	-	Flavin-containing monooxygenase FMO family protein
Os06g0203600	UP	CHI2	Chalcone isomerase domain containing protein
Os06g0223800	UP	CSIL1	C2 calcium-dependent membrane targeting domain containing protein
Os06g0247900	UP	OsGH9B9	Similar to Endo-beta-1,4-glucanase precursor (EC 3.2.1.4)
Os06g0270200	UP	OsHAK24	Similar to Potassium transporter 24
Os06g0284200	UP	-	Protein of unknown function DUF246, plant family protein
Os06g0318500	UP	OsNHX4	Similar to Sodium/hydrogen exchanger
Os06g0529900	UP	-	Hypothetical conserved gene
Os06g0538400	UP	-	Conserved hypothetical protein
Os06g0559400	DOWN	-	Conserved hypothetical protein
Os06g0566500	UP	-	Hypothetical conserved gene
Os06g0573600	UP	OsBgal2	Similar to Beta-galactosidase precursor (EC 3.2.1.23) (Lactase)
Os06g0579200	DOWN	OsLCT1	Low-affinity cation transporter, Regulation of cadmium transport to the grain
Os06g0589500	UP	-	Conserved hypothetical protein
Os06g0594600	UP	OsAT10	BAHD acyltransferase, Glucuronoarabinoxylan modification in grass cell wal
Os06g0595900	UP	LC3	SPOC domain-containing protein, Regulator of leaf inclinatio
Os06g0598800	UP	WSL1	Similar to Fatty acid elongase 1-like protein
Os06g0671000	UP	OsHAK13	Similar to Potassium transporter 1 (AtPOT1) (AtKUP1) (AtKT1)
Os06g0678500	DOWN	-	Hypothetical conserved gene
Os06g0707200	UP	-	Glycosyltransferase AER61, uncharacterized domain containing protein
Os06g0725200	UP	OsGELP90	Lipase, GDSL domain containing protein
Os06g0730150	UP	-	Hypothetical protein
Os07g0116600	DOWN	-	Similar to HLS1 (HOOKLESS 1); N-acetyltransferase
Os07g0121000	UP	-	Protein of unknown function DUF1719, Oryza sativa family protein
Os07g0121750	UP	-	Conserved hypothetical protein
Os07g0122100	UP	-	Protein of unknown function DUF1719, Oryza sativa family protein
Os07g0134200	UP	OsRLCK222	Receptor-like protein kinase 3
Os07g0148800	UP	-	Conserved hypothetical protein
Os07g0192000	UP	-	ATPase, AAA-type, core domain containing protein
Os07g0194800	UP	-	Similar to galactosyltransferase/ transferase, transferring hexosyl groups
Os07g0208500	UP	OsCesA8	Similar to Cellulose synthase-4
Os07g0209100	DOWN	-	Similar to Seed imbibition protein (Fragment)
Os07g0241500	UP	OsUGT710C2	UDP-glucuronosyl/UDP-glucosyltransferase family protein

Os07g0241600	UP	-	UDP-glucuronosyl/UDP-glucosyltransferase domain containing protein
Os07g0252400	UP	OsCesA6	Similar to Cellulose synthase-8
Os07g0274700	UP	-	HvB12D protein (B12Dg1 protein)
Os07g0413800	UP	-	Conserved hypothetical protein
Os07g0414000	UP	-	Transferase domain containing protein
Os07g0418700	UP	OsRePRP2.1	Proline-rich glycoprotein, ABA-dependent inhibition of root growt
Os07g0450100	UP	-	Hypothetical conserved gene
Os07g0457200	UP	-	Hypothetical protein
Os07g0490100	UP	UGT709A4	UDP-glucuronosyl/UDP-glucosyltransferase family protein
Os07g0490500	UP	-	Xanthine/uracil/vitamin C permease family protein
Os07g0523965	UP	-	Similar to Glucose-6-phosphate/phosphate-translocator precursor
Os07g0526400	UP	OsPKS15	Polyketide synthase, type III domain containing protein
Os07g0526600	UP	-	Alpha/beta hydrolase fold-3 domain containing protein
Os07g0550900	UP	-	Similar to Receptor-like protein kinase 6
Os07g0560400	UP	-	Hypothetical protein
Os07g0564500	UP	-	Pyridine nucleotide-disulphide oxidoreductase, NAD-binding region domain containing protein
Os07g0569800	UP	-	Similar to protein kinase
Os07g0572400	UP	-	Conserved hypothetical protein
Os07g0597200	UP	-	Protein kinase, core domain containing protein
Os07g0598000	UP	-	Similar to NADPH HC toxin reductase (Fragment)
Os07g0601600	UP	-	Similar to VEP1 (VEIN PATTERNING 1); binding / catalytic
Os07g0615200	UP	OsJAZ6	Tify domain containing protein
Os07g0622000	UP	SAPK2	Serine/threonine protein kinase, Hyperosmotic stress response, Abscisic acid (ABA)-dependent gene regulatio
Os07g0622200	UP	OsDAHP1	3-Deoxy-D-arabino-heptulosonate 7-phosphate synthase, Chorismate biosyn-thesi
Os07g0628500	UP	OsbHLH1	Basic helix-loop-helix dimerisation region bHLH domain containing protein
Os07g0633600	UP	-	Similar to SUB1; calcium ion binding
Os07g0635500	UP	Os-CYP709C5	Similar to Cytochrome P450
Os07g0656200	DOWN	Os7bglu26	Similar to Beta-glucosidase 26
Os07g0674400	UP	-	Pollen Ole e 1 allergen/extensin domain containing protein
Os07g0683900	UP	-	Ricin B-related lectin domain containing protein
Os07g0685500	UP	-	Alpha/beta hydrolase family protein
Os07g0686300	DOWN	-	Zinc finger, RING/FYVE/PHD-type domain containing protein
Os08g0103100	UP	-	Hypothetical protein
Os08g0137800	UP	OsUCL24	Cupredoxin domain containing protein

Os08g0140300	UP	TDC1	Aromatic L-amino acid decarboxylase (AADC), Senescence-induced serotonin biosynthesis
Os08g0140700	UP	PR5	Similar to CsAtPR5
Os08g0141400	UP	-	Similar to mRNA, clone: RTFL01-03-I20
Os08g0150600	DOWN	-	F-box domain, cyclin-like domain containing protein
Os08g0168000	UP	OsTPS31	Similar to Sesquiterpene synthase
Os08g0200750	DOWN	-	Hypothetical protein
Os08g0238200	UP	-	Dienelactone hydrolase domain containing protein
Os08g0249000	UP	OsBBX25	Zinc finger, B-box domain containing protein
Os08g0280600	UP	-	Reticulon domain containing protein
Os08g0356700	UP	-	Protein of unknown function DUF247, plant family protein
Os08g0374600	UP	OsRLCK253	Similar to Receptor kinase-like protein
Os08g0384500	UP	OsA-BCG44	ABC transporter-like domain containing protein
Os08g0410950	UP	-	Hypothetical conserved gene
Os08g0417000	UP	-	2OG-Fe(II) oxygenase domain containing protein
Os08g0434300	UP	MDH	Similar to Malate dehydrogenase precursor (EC 1.1.1.37)
Os08g0441500	UP	OsCCR	Cinnamoyl-CoA reductase, Lignin formatio
Os08g0453200	DOWN	-	Similar to Auxin-repressed protein
Os08g0460600	DOWN	OsCKX11	Similar to Cytokinin dehydrogenase 11
Os08g0474000	UP	DERF3	Similar to AP2 domain containing protein RAP2.6 (Fragment)
Os08g0489300	UP	TagI	Methyladenine glycosylase domain containing protein
Os08g0490000	UP	OsbHLH031	Helix-loop-helix DNA-binding domain containing protein
Os08g0498400	UP	ROMT-17	Similar to Caffeoyl-CoA 3-O-methyltransferase (Fragment)
Os08g0514033	UP	-	Hypothetical protein
Os08g0520000	UP	BTI-1	Mitochondrial substrate carrier family protein
Os08g0526200	UP	-	Conserved hypothetical protein
Os08g0531100	UP	-	MAP65/ASE1 family protein
Os08g0532200	UP	GSA	Similar to Glutamate-1-semialdehyde 2,1-aminomutase, chloroplast precursor (EC 5.4.3.8) (GSA) (Glutamate-1-semialdehyde aminotransferase) (GSA- AT)
Os08g0542700	UP	-	Ankyrin repeat containing protein
Os08g0549000	DOWN	OsGL1A	R2R3 MYB transcription factor, Homologue of Arabidopsis transcription factor GL
Os08g0560300	UP	-	Protein of unknown function DUF597 family protein
Os09g0106700	UP	R2R3-MYB	Similar to Myb proto-oncogene protein (C-myb)
Os09g0249400	UP	-	Uncharacterised protein family UPF0497, trans-membrane plant domain containing protein

Os09g0255400	UP	IGPS	Similar to Indole-3-glycerol phosphate synthase, chloroplast precursor (EC 4.1.1.48) (IGPS)
Os09g0268100	DOWN	-	Similar to lectin-like receptor kinase 7
Os09g0322400	DOWN	-	Proteolipid membrane potential modulator domain containing protein
Os09g0322500	DOWN	-	Hypothetical conserved gene
Os09g0343600	DOWN	-	Non-protein coding transcript
Os09g0380600	UP	-	Protein of unknown function DUF295 domain containing protein
Os09g0419200	UP	OsCCR19	NAD(P)-binding domain containing protein
Os09g0420300	UP	-	Methyladenine glycosylase domain containing protein
Os09g0426800	UP	WSL2	Homologue of WAX2/GL1, Synthesis of leaf cuticular wax
Os09g0441400	UP	-	Similar to Elicitor-inducible cytochrome P450
Os09g0442100	UP	OsRLCK272	Protein kinase, core domain containing protein
Os09g0450300	UP	-	MAP65/ASE1 family protein
Os09g0456800	UP	HSFB1	Similar to Heat stress transcription factor Spl7 (Heat shock transcription factor) (Heat shock factor RHSF10)
Os09g0471200	UP	-	Similar to WAK80 - OsWAK receptor-like protein kinase
Os09g0494200	UP	BC15	Membrane-associated chitinase-like protein, Cellulose biosynthesi
Os09g0502500	UP	-	Alcohol dehydrogenase superfamily, zinc-containing protein
Os09g0511900	UP	Os9bglu33	Similar to Beta-glucosidase 32
Os09g0524800	UP	OsBAG1	Apoptosis regulator Bcl-2 protein, BAG domain containing protein
Os09g0533900	UP	OsGH9B5	Similar to CEL5=CELLULASE 5 (Fragment)
Os09g0545300	DOWN	SAUR39	SAUR family protein, Negative regulator of auxin synthesis and transpor
Os09g0556700	UP	OsSET37	SET domain domain containing protein
Os09g0562600	UP	WAK92	Wall-associated kinase, Positive regulation of rice blast resistanc
Os10g0127900	UP	OsFbx352	Similar to F-box domain containing protein, expressed
Os10g0144700	UP	-	Cytochrome P450 domain containing protein
Os10g0170000	UP	-	Nucleotide-sensitive chloride conductance regulator family protein
Os10g0181100	DOWN	-	Conserved hypothetical protein
Os10g0195250	DOWN	-	Conserved hypothetical protein
Os10g0392400	UP	Os-TIFY11d	Tify domain containing protein
Os10g0461100	UP	-	Similar to Keratin, type II cytoskeletal 1 (Cytokeratin 1) (K1) (CK 1) (67 kDa cytokeratin) (Hair alpha protein)
Os10g0468500	UP	-	Serine/threonine protein kinase-related domain containing protein
Os10g0469600	UP	-	Similar to Leucine Rich Repeat family protein, expressed
Os10g0478100	UP	-	Heat shock protein DnaJ, N-terminal domain containing protein
Os10g0503250	UP	-	Hypothetical protein
Os10g0512800	UP	OsRF-PHC-10	Similar to predicted protein

Os10g0516200	UP	OsRLCK3 03	Serine/threonine protein kinase domain containing protein
Os10g0517500	UP	RRJ1	Cys/Met metabolism, pyridoxal phosphate-dependent enzyme domain containing protein
Os10g0532600	UP	OsOFP17	Protein of unknown function DUF623, plant domain containing protein
Os10g0533150	UP	-	Similar to BRASSINOSTEROID INSENSITIVE 1-associated receptor kinase 1
Os10g0534700	UP	-	Protein of unknown function DUF604 family protein
Os10g0536400	UP	OsF3H-2	Similar to Oxidoreductase, 2OG-Fe oxygenase family protein, expressed
Os10g0537800	UP	-	Peptidase aspartic, catalytic domain containing protein
Os10g0575000	UP	OsbHLH0 09	JAZ-interacting transcription factor, Positive regulator of jasmonate (JA) signaling pathway, Bacterial blight resistance, Spikelet developmen
Os10g0577600	UP	JMJ706	H3K9 demethylase, Floral organ developmen
Os10g0580900	UP	-	Conserved hypothetical protein
Os11g0100600	UP	-	Alpha/beta hydrolase family protein
Os11g0108800	UP	-	Similar to Protein kinase domain containing protein, expressed
Os11g0109700	UP	-	Protein of unknown function Cys-rich family protein
Os11g0116400	UP	-	Similar to Elongation factor P (EF-P)
Os11g0155100	UP	OsFbox56 8	Similar to F-box domain containing protein
Os11g0178800	UP	-	Plant disease resistance response protein family protein
Os11g0180000	UP	-	Plant disease resistance response protein family protein
Os11g0246166	DOWN	-	Hypothetical protein
Os11g0249300	UP	-	Conserved hypothetical protein
Os11g0257800	DOWN	-	Hypothetical protein
Os11g0275200	UP	-	Similar to Endo-1,3;1,4-beta-D-glucanase precursor (EC 3.2.1.-)
Os11g0275500	UP	-	Similar to Endo-1,3;1,4-beta-D-glucanase precursor (EC 3.2.1.-)
Os11g0444700	DOWN	OsPB1	Octicosapeptide/Phox/Bem1p domain containing protein
Os11g0444900	DOWN	-	Octicosapeptide/Phox/Bem1p domain containing protein
Os11g0448700	UP	OsRALF-31	Conserved hypothetical protein
Os11g0455800	UP	OsSHM2	Similar to Serine hydroxymethyltransferase
Os11g0482901	UP	-	Hypothetical gene
Os11g0496500	UP	-	Similar to AT.I.24-5 protein (Fragment)
Os11g0531700	UP	-	NUDIX hydrolase domain containing protein
Os11g0536000	UP	-	Non-protein coding transcript
Os11g0566800	UP	OsPKS27	Similar to Bibenzyl synthase (EC 2.3.1.-)
Os11g0569300	DOWN	-	Serine/threonine protein kinase domain containing protein
Os11g0592600	UP	-	Targeting for Xklp2 family protein
Os11g0619800	UP	-	Kelch related domain containing protein
Os11g0629400	UP	-	Similar to Nitrilase associated protein-like
Os11g0644700	UP	-	Plant disease resistance response protein family protein

Os11g0645400	UP	-	Plant disease resistance response protein family protein
Os11g0671100	DOWN	-	Hypothetical protein
Os11g0674400	UP	-	Similar to NB-ARC domain containing protein, expressed
Os11g0701700	UP	-	Hypothetical gene
Os12g0106300	UP	OsTBL1	Xylan acetyltransferase, Resistance to bacterial leaf blight diseas
Os12g0108100	UP	-	Similar to Ser Thr specific protein kinase-like protein
Os12g0115900	UP	-	Translation elongation factor P domain containing protein
Os12g0149900	UP	-	IQ calmodulin-binding region domain containing protein
Os12g0152100	UP	-	Alpha/beta hydrolase fold-1 domain containing protein
Os12g0174200	UP	-	Conserved hypothetical protein
Os12g0182500	UP	-	Conserved hypothetical protein
Os12g0190400	UP	-	Similar to NifS-like protein
Os12g0225900	UP	-	Allyl alcohol dehydrogenase
Os12g0443000	UP	-	Similar to Cytochrome P450 family protein, expressed
Os12g0443500	UP	-	Similar to UDP-glucose 6-dehydrogenase (EC 1.1.1.22) (UDP-Glc dehydrogen-ase) (UDP-GlcDH) (UDPGDH)
Os12g0485150	UP	-	Conserved hypothetical protein
Os12g0530100	UP	PRX138	Similar to Peroxidase 24 precursor (EC 1.11.1.7) (Atperox P24) (ATP47)
Os12g0548300	UP	OsNDPK2	Nucleoside diphosphate kinase, Regulation of chloroplast development and chlorophyll biosynthesis, Abiotic stress response (ABA and salinity
Os12g0573600	UP	-	Similar to predicted protein
Os12g0604300	UP	-	Hypothetical gene
Os12g0616400	UP	OsPCF8	Similar to Isoform 2 of Transcription factor PCF8
Os12g0624800	DOWN	-	Conserved hypothetical protein
Os12g0629300	UP	-	Similar to Thaumatin-like protein
Os12g0635400	UP	-	VQ domain containing protein
**Specific DEGs in B3_vs_A3 comparing B1_vs_A1, B2_vs_A2 and B3_vs_A3**			
**Gene ID**	**up/down**	**Gene name**	**Description**
ENSRNA049471366	UP	MIR159	microRNA MIR159
ENSRNA049475944	UP	LSU_rRNA_eu-karya	Eukaryotic large subunit ribosomal RNA
ENSRNA049476502	UP	5_8S_rRNA	5.8S ribosomal RNA
Os01g0110200	UP	OsAGP12	Conserved hypothetical protein
Os01g0110400	UP	-	Similar to Acetyl-CoA C-acetyltransferase
Os01g0112600	DOWN	-	Conserved hypothetical protein
Os01g0113200	DOWN	OsRLCK2	Similar to Receptor serine/threonine kinase
Os01g0114550	UP	-	Non-protein coding transcript

Os01g0115600	DOWN	OsRLCK11	Similar to LRK14
Os01g0117100	DOWN	-	Similar to Receptor kinase LRK10
Os01g0117300	DOWN	OsRLCK17	Similar to Receptor kinase LRK10
Os01g0117700	DOWN	-	Similar to LRK14
Os01g0118066	DOWN	-	Non-protein coding transcript
Os01g0123700	UP	-	Zinc finger, RING-type domain containing protein
Os01g0124900	DOWN	-	Seven-in-absentia protein, sina domain containing protein
Os01g0127400	DOWN	-	Protein of unknown function DUF3326 domain containing protein
Os01g0127600	UP	-	Similar to Bowman-Birk type proteinase inhibitor D-II precursor (IV)
Os01g0132000	UP	IAI2	Similar to Wound-induced protease inhibitor (WIP1)
Os01g0134500	DOWN	OsSTE1	Similar to Delta-7-sterol-C5(6)-desaturase (EC 1.3.3.-) (Delta-7-C-5 sterol desaturase) (Delta7-sterol-C5-desaturase)
Os01g0134700	DOWN	-	Calmodulin binding protein-like family protein
Os01g0136450	DOWN	-	Conserved hypothetical protein
Os01g0137700	DOWN	-	Conserved hypothetical protein
Os01g0137800	DOWN	-	Non-protein coding transcript
Os01g0138900	UP	-	Mandelate racemase/muconate lactonizing enzyme family protein
Os01g0141700	DOWN	OsWD40-2	Similar to predicted protein
Os01g0142100	DOWN	-	Peptidase M50 family protein
Os01g0143300	UP	-	Similar to Glycine-rich protein (Fragment)
Os01g0144000	UP	-	Conserved hypothetical protein
Os01g0147900	UP	OsTPI1	Triosephosphate isomerase, Glycolytic enzyme, Key component in XA3/XA26-mediated resistance to Xo
Os01g0148000	DOWN	-	Similar to predicted protein
Os01g0150700	DOWN	-	Hypothetical conserved gene
Os01g0152951	DOWN	-	Hypothetical conserved gene
Os01g0154000	DOWN	-	Conserved hypothetical protein
Os01g0160100	UP	-	Similar to Pyruvate decarboxylase isozyme 2 (EC 4.1.1.1) (PDC) (Fragment)
Os01g0161300	DOWN	-	Hypothetical conserved gene
Os01g0162001	DOWN	-	Hypothetical gene
Os01g0162200	DOWN	-	Leucine-rich repeat domain containing protein
Os01g0166800	DOWN	-	Similar to ETG1 (E2F TARGET GENE 1)
Os01g0168800	UP	-	Alpha/beta hydrolase fold-1 domain containing protein
Os01g0173000	DOWN	-	Putative thiol-disulphide oxidoreductase DCC domain containing protein
Os01g0178400	DOWN	-	Similar to Protein phosphatase 2A B'kappa subunit
Os01g0179800	DOWN	-	TMS membrane protein/tumour differentially expressed protein family protein
Os01g0180300	DOWN	-	Lipoprotein, type 6 family protein

Os01g0180800	UP	-	Heat shock protein Hsp70 family protein
Os01g0188200	DOWN	-	Endonuclease/exonuclease/phosphatase domain containing protein
Os01g0188900	DOWN	-	Conserved hypothetical protein
Os01g0192000	DOWN	-	Similar to Ankyrin-like protein-like protein
Os01g0192550	UP	DOS	CCCH-type zinc finger protein, Negative regulator for leaf senescenc
Os01g0195300	DOWN	-	Conserved hypothetical protein
Os01g0197350	DOWN	-	Hypothetical conserved gene
Os01g0198500	UP	-	Hypothetical genes
Os01g0205200	DOWN	-	Conserved hypothetical protein
Os01g0205500	UP	-	Pentatricopeptide repeat domain containing protein
Os01g0208000	UP	-	Similar to 60S ribosomal protein L11-2 (L16). Splice isoform 2
Os01g0210900	UP	OsUMA-MIT4	Protein of unknown function DUF6, transmembrane domain containing protein
Os01g0215750	UP	-	Cytochrome P450 family protein
Os01g0216000	UP	-	Hypothetical gene
Os01g0217500	DOWN	OsGELP7	Lipase, GDSL domain containing protein
Os01g0218100	UP	OsDJ-1A	ThiJ/PfpI domain containing protein
Os01g0223200	UP	OsbHLH037	Helix-loop-helix DNA-binding domain containing protein
Os01g0225550	UP	OsGELP12	Lipase, GDSL domain containing protein
Os01g0226300	UP	-	Hypothetical gene
Os01g0226600	UP	-	Reticulon family protein
Os01g0227800	DOWN	SLAC	Similar to C4-dicarboxylate transporter/malic acid transport protein
Os01g0229900	DOWN	-	Similar to cDNA, clone: J075083L18, full insert sequence
Os01g0231700	UP	-	Similar to Ubiquitin-conjugating enzyme family protein, expressed
Os01g0231800	UP	-	Similar to 60S acidic ribosomal protein P2-B (CaRP2B)
Os01g0234001	DOWN	-	Similar to cDNA clone:J033025F17, full insert sequence
Os01g0236900	DOWN	-	Hypothetical conserved gene
Os01g0239200	DOWN	-	Hypothetical protein
Os01g0242300	UP	OsTPT1	Similar to Phophate translocator (Fragment)
Os01g0257100	UP	-	Optic atrophy 3 family protein
Os01g0260500	DOWN	OsRALF-5	Rapid ALkalinization Factor family protein
Os01g0262700	DOWN	-	Hypothetical conserved gene
Os01g0268300	DOWN	-	Similar to predicted protein
Os01g0272600	UP	-	Similar to DNA gyrase B subunit
Os01g0274100	UP	OsRDN	Similar to Root determined nodulation 1
Os01g0274800	UP	OsYUCCA10	Similar to disulfide oxidoreductase/ monooxygenase/ oxidoreductase
Os01g0276700	UP	CSA	R2R3-type MYB transcription factor, Sugar partitioning into anthe
Os01g0277500	DOWN	-	Similar to Pyruvate kinase, cytosolic isozyme (EC 2.7.1.40) (PK)
Os01g0278000	DOWN	-	Conserved hypothetical protein

Os01g0278050	DOWN	OsDof4	Dof (DNA binding with one finger) transcription factor, C2-C2 zinc finger transcription factor, Regulation of flowering tim
Os01g0279400	DOWN	OsVQ1	VQ domain containing protein
Os01g0281100	UP	-	Hypothetical conserved gene
Os01g0283600	UP	OsZIFL2	Major facilitator superfamily antiporter
Os01g0283700	UP	-	Conserved hypothetical protein
Os01g0286600	DOWN	OsCCR4	Similar to Cinnamoyl CoA reductase
Os01g0287400	UP	OsCCR5	Similar to Cinnamoyl-CoA reductase (EC 1.2.1.44)
Os01g0290650	UP	PPX	Similar to Plastidal protoporphyrinogen oxidase
Os01g0290700	UP	-	Similar to Hydrophobic protein LTI6A (Low temperature-induced protein 6A)
Os01g0290901	DOWN	-	Non-protein coding transcript
Os01g0292700	DOWN	OsABCB1	Similar to CjMDR1
Os01g0293000	DOWN	-	Non-protein coding transcript
Os01g0296166	UP	OsIF	Intermediate filament, Cytoskeleton protein, Tolerance to salinity and heat stres
Os01g0297200	UP	OsSAMS3	S-adenosyl-l-methionine synthetase, Histone H3K4me3 and DNA methylation, Regulation of gene expression related to flowerin
Os01g0301500	DOWN	-	Hypothetical protein
Os01g0302100	UP	-	ATPase, AAA-type, core domain containing protein
Os01g0306800	DOWN	-	Pentatricopeptide repeat domain containing protein
Os01g0309800	UP	-	Conserved hypothetical protein
Os01g0311100	DOWN	-	Conserved hypothetical protein
Os01g0314100	UP	-	Similar to Hydrogenase expression/formation protein hypB
Os01g0320700	DOWN	OsRFPH2-4	Similar to RING-H2 finger protein ATL2L
Os01g0321900	DOWN	-	Catalytic domain of components of various dehydrogenase complexes containing protein
Os01g0323300	DOWN	-	Hypothetical protein
Os01g0327100	UP	-	Hypothetical conserved gene
Os01g0337100	UP	-	Similar to Ser Thr specific protein kinase-like protein
Os01g0346250	DOWN	-	RNA-binding, CRM domain domain containing protein
Os01g0348900	UP	prx15	Haem peroxidase family protein
Os01g0349600	DOWN	OsTPS1	Similar to Sesquiterpene synthase
Os01g0351200	DOWN	-	Conserved hypothetical protein
Os01g0354700	DOWN	OsJRL	Jacalin-related mannose-binding lectin, Salinity toleranc
Os01g0358100	UP	-	Hypothetical protein
Os01g0362100	DOWN	PARP2A	Similar to Poly
Os01g0364400	DOWN	-	Pre-mRNA cleavage complex II Clp1 domain containing protein
Os01g0369700	UP	OsRALF-6	Rapid ALkalinization Factor family protein
Os01g0371200	UP	-	Esterase/lipase/thioesterase domain containing protein

Os01g0382450	UP	OsRLCK35	Protein kinase, catalytic domain domain containing protein
Os01g0384800	DOWN	OsGSTF5	Similar to Glutathione S-transferase GST 8 (EC 2.5.1.18)
Os01g0389700	DOWN	OsOPR10	Similar to 12-oxo-phytodienoic acid reductase6
Os01g0505700	UP	OsGSTF1	Similar to Glutathione-S-transferase 19E50
Os01g0510600	DOWN	DHQDT/SDH	Similar to shikimate biosynthesis protein aroDE
Os01g0511100	UP	-	Similar to Kinesin heavy chain (Fragment)
Os01g0520180	DOWN	-	Conserved hypothetical protein
Os01g0520600	UP	-	Protein of unknown function DUF679 family protein
Os01g0521250	UP	-	Conserved hypothetical protein
Os01g0533900	UP	OsFLU1	Tetratricopeptide-like helical domain containing protein
Os01g0536000	DOWN	-	UspA domain containing protein
Os01g0550600	DOWN	-	Hypothetical conserved gene
Os01g0552000	DOWN	-	NB-ARC domain containing protein
Os01g0557500	DOWN	-	Non-protein coding transcript
Os01g0558850	DOWN	OsABCB2	Similar to Multidrug resistance protein 1 homolog
Os01g0559600	DOWN	OsCIPK08	Similar to CBL-interacting serine/threonine-protein kinase 24 (EC 2.7.1.37) (SNF1-related kinase 3.11) (SALT OVERLY SENSITIVE 2 protein)
Os01g0559900	UP	-	Conserved hypothetical protein
Os01g0565900	UP	-	Hypothetical conserved gene
Os01g0570800	DOWN	SRZ3	Similar to TATA-binding protein associated factor 2N (RNA-binding protein 56) (TAFII68) (TAF(II)68)
Os01g0575200	DOWN	OsCAX1a	Cation/proton exchanger 1a
Os01g0579600	DOWN	-	Similar to peptidase M16 family protein / insulinase family protein
Os01g0582600	DOWN	OsVPE2	Similar to C13 endopeptidase NP1 precursor
Os01g0588200	UP	OsAGP14	Non-protein coding transcript
Os01g0589000	DOWN	-	Similar to Protein transport protein Sec61 beta subunit
Os01g0594800	UP	-	IQ calmodulin-binding region domain containing protein
Os01g0595600	UP	OsbHLH024	Helix-loop-helix DNA-binding domain containing protein
Os01g0600000	UP	OsCBL9	Similar to Calcineurin B-like protein 9
Os01g0600900	DOWN	-	Phospholipase A2, active site domain containing protein
Os01g0607800	DOWN	OSVDAC3	Voltage-dependent anion channel
Os01g0611100	UP	-	Nucleic acid-binding, OB-fold domain containing protein
Os01g0615100	UP	-	Similar to D-amino acid oxidase
Os01g0615200	UP	-	Alpha/beta hydrolase fold-1 domain containing protein
Os01g0618400	DOWN	OsATL15	Amino acid transporter, transmembrane domain containing protein
Os01g0626400	UP	-	Similar to Copia-like retroelement pol polyprotein

Os01g0627500	UP	CAB2R	Chlorophyll a-b binding protein 2, chloroplast precursor (LHCII type I CAB-2) (LHCP)
Os01g0647000	UP	-	Pentatricopeptide repeat domain containing protein
Os01g0647200	DOWN	OsRAN1	Similar to GTP-binding nuclear protein Ran-2
Os01g0650800	DOWN	OCPI2	Chymotrypsin protease inhibitor, Salt and osmotic stress toleranc
Os01g0652375	DOWN	OsAT2	Transferase family protein
Os01g0654500	UP	OsRH25	Similar to RNA helicase (Fragment)
Os01g0661500	DOWN	WRKY11	WRKY transcription factor, Control of flowering time and plant heigh
Os01g0675800	DOWN	Os-CYP72A18	Cytochrome P450 family protein
Os01g0676200	DOWN	OsFbox031	Cyclin-like F-box domain containing protein
Os01g0678000	DOWN	-	Hypothetical conserved gene
Os01g0678100	DOWN	-	Transposon, En/Spm-like domain containing protein
Os01g0681600	DOWN	-	Hypothetical protein
Os01g0681800	DOWN	-	Similar to NADP-isocitrate dehydrogenase
Os01g0686200	DOWN	-	Mov34/MPN/PAD-1 family protein
Os01g0688200	UP	ONAC014	No apical meristem (NAM) protein domain containing protein
Os01g0696800	DOWN	-	Conserved hypothetical protein
Os01g0702000	DOWN	-	Protein of unknown function DUF2921 domain containing protein
Os01g0702450	UP	-	Protein of unknown function DUF2921 domain containing protein
Os01g0704100	DOWN	-	Hypothetical conserved gene
Os01g0705000	DOWN	-	Conserved hypothetical protein
Os01g0706400	DOWN	-	UDP-glucuronosyl/UDP-glucosyltransferase family protein
Os01g0706600	UP	-	Similar to hydrolase, alpha/beta fold family protein
Os01g0708500	UP	-	Peptidase A1 domain containing protein
Os01g0711000	UP	OsBBD1	Protein of unknown function DUF151 domain containing protein
Os01g0714600	DOWN	-	Similar to Chaperone protein dnaJ 10
Os01g0716500	UP	NRT2.3	Nitrate transporter, Nitrate transporter
Os01g0718300	DOWN	-	Hypothetical conserved gene
Os01g0719800	DOWN	-	Protein of unknown function DUF292, eukaryotic domain containing protein
Os01g0720200	UP	-	Conserved hypothetical protein
Os01g0720400	UP	LOGL1	Similar to lysine decarboxylase-like protein
Os01g0725000	DOWN	-	Similar to Vacuolar ATP synthase subunit B isoform 2 (EC 3.6.3.14) (V-ATPase B subunit 2) (Vacuolar proton pump B subunit 2)
Os01g0725800	DOWN	-	Similar to cDNA clone:J023088C01, full insert sequence
Os01g0726400	DOWN	-	Methyltransferase type 12 domain containing protein
Os01g0727800	DOWN	D61	Receptor serine/threonine kinase, Organ development through controlling cell division and elongatio
Os01g0727820	DOWN	-	Hypothetical protein
Os01g0727840	DOWN	-	Non-protein coding transcript

Os01g0732801	DOWN	OsACP1	HAD-superfamily subfamily IB hydrolase, hypothetical 1 protein
Os01g0733001	DOWN	-	Similar to H0402C08.3 protein
Os01g0733200	DOWN	OsWD40-24	WD40/YVTN repeat-like domain containing protein
Os01g0735900	UP	MADS32	MADS box transcription factor, Regulation of floral organ identit
Os01g0736100	UP	OsSub3	Peptidase S8, subtilisin-related domain containing protein
Os01g0738800	UP	-	Hypothetical protein
Os01g0740600	UP	OsSub3	Similar to subtilase family protein
Os01g0740650	UP	-	Conserved hypothetical protein
Os01g0740700	UP	-	Similar to NRAMP3 (NRAMP metal ion transporter 3); manganese ion transmembrane transporter/ metal ion transmembrane transporter
Os01g0749200	DOWN	OsHsfC1b	Similar to Heat shock transcription factor 29 (Fragment)
Os01g0752200	UP	OsUGT706B1	UDP-glucuronosyl/UDP-glucosyltransferase family protein
Os01g0764400	UP	OsUGT706C1	UDP-glucuronosyl/UDP-glucosyltransferase family protein
Os01g0767600	UP	OsPFK02	Conserved hypothetical protein
Os01g0768100	DOWN	-	Transferrin receptor-like, dimerisation domain containing protein
Os01g0769700	DOWN	-	Similar to glutamate carboxypeptidase 2
Os01g0770000	DOWN	-	Hypothetical protein
Os01g0770200	DOWN	-	Hypothetical conserved gene
Os01g0777700	DOWN	WLP1	Chloroplast ribosome L13 protein, Chloroplast development under low temperature condition
Os01g0781200	DOWN	OsCesA4	Similar to Cellulose synthase (Fragment)
Os01g0782200	DOWN	NOG1	Enoyl-CoA hydratase/isomerase, Regulation of grain number and the yiel
Os01g0787600	UP	-	Similar to Dynein light chain
Os01g0788400	UP	CM	Similar to Chorismate mutase, chloroplast precursor (EC 5.4.99.5) (CM-1)
Os01g0788451	UP	-	Conserved hypothetical protein
Os01g0793800	UP	-	Conserved hypothetical protein
Os01g0795600	DOWN	DRUS2	Receptor-like kinase, Control of reproductive growth and development, Repression of cell deat
Os01g0798500	DOWN	-	Uncharacterised protein family UPF0089 domain containing protein
Os01g0808100	DOWN	TYDC	Aromatic L-amino acid decarboxylase (AADC), Conversion of tyrosine into tyramin
Os01g0812900	UP	OsCKX5	Similar to Cytokinin dehydrogenase 5 precursor (EC 1.5.99.12) (Cytokinin oxidase 5) (CKO5) (AtCKX5) (AtCKX6)
Os01g0813000	DOWN	-	Similar to acid phosphatase/ oxidoreductase/ transition metal ion binding
Os01g0813800	DOWN	Pi64	Similar to Rust resistance protein
Os01g0814800	DOWN	-	Diacylglycerol kinase, catalytic region domain containing protein
Os01g0814900	UP	OsPOP4	Similar to Salicylic acid-binding protein 2
Os01g0821600	DOWN	OsPME6	Similar to Pectinesterase (EC 3.1.1.11) (Fragment)

Os01g0824700	DOWN	-	Hypothetical protein
Os01g0825800	UP	-	Di-copper centre-containing domain containing protein
Os01g0826400	DOWN	-	Conserved hypothetical protein
Os01g0829000	DOWN	Hwi2	Similar to Subtilase
Os01g0829100	DOWN	-	Conserved hypothetical protein
Os01g0832600	UP	-	Similar to predicted protein
Os01g0833150	DOWN	OsbZIP08	Similar to BZIP transcription factor
Os01g0833600	DOWN	-	Similar to alphavirus core protein family
Os01g0834100	UP	-	Similar to 14 kDa zinc-binding protein
Os01g0837000	DOWN	Os1bglu3	Similar to Beta-glucosidase 3
Os01g0838350	UP	-	Similar to Cysteine synthase, chloroplast precursor (EC 2.5.1.47
Os01g0838600	UP	NFR	Similar to Cytochrome b5 reductase
Os01g0839300	UP	OsWRKY21	WRKY transcription factor 48-like protein (WRKY transcription factor 21)
Os01g0841600	UP	OsFbox048	Cyclin-like F-box domain containing protein
Os01g0841700	DOWN	OsATL7	Amino acid transporter, transmembrane domain containing protein
Os01g0841800	DOWN	OsWRKY24	WRKY transcription factor 24 (WRKY24)
Os01g0842200	DOWN	-	Thioredoxin-like fold domain containing protein
Os01g0845000	DOWN	-	Ankyrin domain containing protein
Os01g0846000	DOWN	-	Similar to Leucoanthocyanidin dioxygenase-like protein
Os01g0847700	UP	-	Similar to predicted protein
Os01g0847800	UP	-	Conserved hypothetical protein
Os01g0848900	UP	SIP1	Aldehyde dehydrogenase, conserved site domain containing protein
Os01g0855200	UP	OsNPR4	Ankyrin repeat containing protein
Os01g0857500	DOWN	OsIPS2	Conserved hypothetical protein
Os01g0864500	DOWN	-	Zinc finger, C2H2-like domain containing protein
Os01g0866400	DOWN	-	Similar to 50S ribosomal protein L17
Os01g0871300	UP	OscTPI	Triose phosphate isomerase (EC 5.3.1.1), Abiotic stress response, Response to methylglyoxal (cytotoxin
Os01g0871800	DOWN	Rpp17	Similar to Isoform ERG1b of Elicitor-responsive protein 1
Os01g0871900	DOWN	-	Ribonuclease II and R domain containing protein
Os01g0873200	DOWN	-	Similar to Scarecrow-like 9 (Fragment)
Os01g0874800	DOWN	-	Hypothetical conserved gene
Os01g0881300	DOWN	-	Nucleic acid-binding, OB-fold domain containing protein
Os01g0881800	DOWN	AKR2	Aldo-keto reductase, Detoxification of reactive aldehydes, Abiotic stress toler-anc
Os01g0882200	DOWN	AKR3	Similar to Aldose reductase (EC 1.1.1.21) (AR) (Aldehyde reductase) (20-al-pha- hydroxysteroid dehydrogenase) (EC 1.1.1.149) (20-alpha-HSD)
Os01g0883100	DOWN	-	Conserved hypothetical protein

Os01g0885500	DOWN	-	Tetratricopeptide-like helical domain containing protein
Os01g0890600	DOWN	-	Xanthine/uracil/vitamin C permease family protein
Os01g0891400	DOWN	ENAC1	NAC (NAM ATAF1/2 CUC2) transcription factor, Early and transient regulator of abiotic stress response
Os01g0891601	DOWN	OsSDS1	Harpin-induced 1 domain containing protein
Os01g0891700	UP	FR	Similar to Fructose-1,6-bisphosphatase (EC 3.1.3.11) (Fragment)
Os01g0893700	UP	-	Pyridoxal phosphate-dependent transferase, major region domain containing protein
Os01g0901900	DOWN	-	TGF-beta receptor, type I/II extracellular region family protein
Os01g0902100	DOWN	-	Similar to POT family protein
Os01g0905200	DOWN	-	Similar to Amidophosphoribosyltransferase, chloroplast precursor (EC 2.4.2.14) (Glutamine phosphoribosylpyrophosphate amidotransferase) (ATASE) (GPAT) (Fragment)
Os01g0905300	DOWN	-	Similar to DNA polymerase I
Os01g0905700	UP	-	Conserved hypothetical protein
Os01g0908700	DOWN	OsSWEET1a	Non-protein coding transcript
Os01g0909400	DOWN	-	Conserved hypothetical protein
Os01g0912850	DOWN	-	Similar to octopine synthase binding factor3
Os01g0915900	DOWN	OsMADS2	Similar to PISTILLATA-like MADS box protein
Os01g0916100	DOWN	-	Hypothetical conserved gene
Os01g0919400	DOWN	-	Protein kinase, catalytic domain domain containing protein
Os01g0920300	DOWN	-	S1, RNA binding domain containing protein
Os01g0922700	UP	-	Leucine-rich repeat, N-terminal domain containing protein
Os01g0924200	UP	-	Leucine-rich repeat, N-terminal domain containing protein
Os01g0926300	UP	-	DOMON domain domain containing protein
Os01g0930000	DOWN	-	S1, RNA binding domain containing protein
Os01g0930400	UP	OsABCC2	Similar to MRP-like ABC transporter
Os01g0931100	DOWN	OsEXO70FX15	Exo70 exocyst complex subunit family protein
Os01g0937100	UP	OsEXO70FX14	Similar to Leucine zipper protein-like
Os01g0940000	UP	-	Zinc finger, RING/FYVE/PHD-type domain containing protein
Os01g0940800	UP	OsRFP	Similar to Hnrpa2b1-prov protein
Os01g0945300	DOWN	-	Protein of unknown function DUF868, plant family protein
Os01g0948500	DOWN	-	Conserved hypothetical protein
Os01g0948600	UP	-	Conserved hypothetical protein
Os01g0949300	DOWN	-	Similar to loricrin
Os01g0949700	UP	SPS1	Sucrose phosphate synthase, Sucrose synthesis in pollen germinatio
Os01g0952500	UP	-	Methyltransferase small domain containing protein
Os01g0952900	DOWN	-	Conserved hypothetical protein

Os01g0959750	DOWN	-	Conserved hypothetical protein
Os01g0960800	UP	-	Similar to Transaldolase (EC 2.2.1.2)
Os01g0962600	UP	-	Similar to CM0545.330.nc protein
Os01g0971600	DOWN	HAK5	Potassium transporter, High-affinity K acquisition, Root-to-shoot K transport, K-regulated salt toleranc
Os01g0975000	DOWN	-	Conserved hypothetical protein
Os01g0975900	UP	-	Similar to Xylanase inhibitor precursor (Xylanase inhibitor TAXI-I)
Os01g0977250	UP	CKX4	Cytokinin oxidase/dehydrogenase, Crown root formatio
Os01g0978500	DOWN	Gns6	Similar to Beta-1,3-glucanase precursor
Os02g0101000	UP	OsBAT4	Amino acid/polyamine transporter I family protein
Os02g0101750	DOWN	-	Conserved hypothetical protein
Os02g0107900	DOWN	-	Armadillo-like helical domain containing protein
Os02g0109200	DOWN	-	Conserved hypothetical protein
Os02g0109900	DOWN	-	EF-Hand type domain containing protein
Os02g0114033	DOWN	OsGSTU7	Glutathione S-transferase, C-terminal domain containing protein
Os02g0114200	UP	RR4	A-type response regulator, Cytokinin signalin
Os02g0115900	UP	-	Conserved hypothetical protein
Os02g0120000	DOWN	-	Conserved hypothetical protein
Os02g0122600	DOWN	-	Protein Transporter, Pam16 family protein
Os02g0122900	DOWN	RPS10	Similar to 40S ribosomal protein S10-1
Os02g0134200	DOWN	OsSTA45	Similar to Sn-glycerol-3-phosphate dehydrogenase (Fragment)
Os02g0137450	DOWN	OsDSR3	Protein of unknown function DUF966 family protein
Os02g0138100	DOWN	OsMYB48	MYB-related transcription factor, Drought and salinity toleranc
Os02g0139400	DOWN	OsTIP1	Similar to Tonoplast membrane integral protein ZmTIP1-2
Os02g0141100	DOWN	-	Heavy metal transport/detoxification protein domain containing protein
Os02g0145200	UP	-	Hypothetical gene
Os02g0150600	DOWN	-	Conserved hypothetical protein
Os02g0152900	DOWN	OsATL11	Amino acid transporter, transmembrane domain containing protein
Os02g0153200	UP	-	Hypothetical gene
Os02g0154000	DOWN	-	Similar to ABC transporter family, cholesterol/phospholipid flippase
Os02g0154200	DOWN	-	Protein of unknown function DUF793 family protein
Os02g0161100	DOWN	-	Hypothetical conserved gene
Os02g0161200	DOWN	-	Glutamine-Leucine-Glutamine, QLQ domain containing protein
Os02g0161300	DOWN	CBP3	Serine carboxypeptidase III precursor (EC 3.4.16.5)
Os02g0162600	DOWN	OsBiP1	Endosperm lumenal binding protein
Os02g0164800	UP	-	Pentatricopeptide repeat domain containing protein
Os02g0165100	DOWN	-	EF-Hand type domain containing protein
Os02g0165200	DOWN	-	Similar to Dimethyladenosine transferase
Os02g0168200	UP	CDPK4	Calcium-dependent protein kinase, Positive regulator of the salt and drought stress response
Os02g0173100	DOWN	OsSGL	DUF1645 family protein, Regulation of stress-tolerance and grain lengt

Os02g0174300	DOWN	OsHAC70 3	Conserved hypothetical protein
Os02g0181300	DOWN	-	Conserved hypothetical protein
Os02g0184300	DOWN	OsSPL3	Similar to Squamosa promoter-binding-like protein 3
Os02g0184900	UP	OsARF5	Similar to Auxin response factor 5
Os02g0190300	UP	-	Conserved hypothetical protein
Os02g0193732	UP	-	Similar to Pyridine nucleotide-disulphide oxidoreductase (Fragment)
Os02g0198500	UP	YL1	Nucleus-encoded chloroplast protein, Chloroplast development, Biogenesis of chloroplast ATP synthas
Os02g0198700	DOWN	LRK7	Leucine-rich repeat receptor-like kinas
Os02g0199200	DOWN	LRK2	Leucine-rich repeat receptor-like kinase, Positive regulation of the drought stress response and tiller developmen
Os02g0199300	DOWN	LRK1	Leucine-rich repeat receptor kinase, Regulation of grain yiel
Os02g0203300	UP	OsFH10	Actin-binding FH2 domain containing protein
Os02g0205100	UP	OsC3H13	Zinc finger, CCCH-type domain containing protein
Os02g0206700	UP	-	Similar to Mot1 (Fragment)
Os02g0209300	DOWN	-	Conserved hypothetical protein
Os02g0209900	UP	-	Zinc finger, C2H2-type domain containing protein
Os02g0211000	DOWN	OsRLCK6 3	Protein kinase, core domain containing protein
Os02g0211350	UP	-	Hypothetical protein
Os02g0211500	DOWN	-	Similar to Transfactor-like protein
Os02g0212400	UP	CYP97B4	Similar to cytochrome P450
Os02g0212900	DOWN	-	Hypothetical conserved gene
Os02g0215200	UP	4CL3	4-coumarate:coenzyme A ligase, Lignin biosynthesis, Defense against woundin
Os02g0215700	DOWN	WRKY71	WRKY transcription factor, Defense respons
Os02g0216400	DOWN	-	Hypothetical protein
Os02g0221900	UP	-	Cytochrome P450 family protein
Os02g0224800	UP	GH2	Cinnamyl-alcohol dehydrogenase, Lignin biosynthesis, Monolignol biosyn-thesi
Os02g0227200	UP	OsABCB9	P-glycoprotein homologue
Os02g0227900	UP	-	Hypothetical conserved gene
Os02g0234500	DOWN	-	Conserved hypothetical protein
Os02g0240100	UP	OsSub12	Similar to Subtilisin-like protease
Os02g0241200	DOWN	-	Disease resistance protein domain containing protein
Os02g0241300	UP	OsFKBP16 -1	Peptidyl-prolyl cis-trans isomerase, FKBP-type domain containing protein
Os02g0244700	UP	-	Similar to UDP-glycosyltransferase UGT75E3
Os02g0247800	DOWN	-	Conserved hypothetical protein
Os02g0251800	DOWN	-	UDP-glucuronosyl/UDP-glucosyltransferase family protein

Os02g0251900	DOWN	-	Hypothetical conserved gene
Os02g0255700	DOWN	-	Syntaxin, N-terminal domain containing protein
Os02g0255801	DOWN	OsABCA1	Conserved hypothetical protein
Os02g0259600	DOWN	-	Hypothetical gene
Os02g0260500	DOWN	-	Non-protein coding transcript
Os02g0261100	UP	-	Conserved hypothetical protein
Os02g0269200	DOWN	OsRLCK66	Similar to Protein kinase domain containing protein, expressed
Os02g0271000	DOWN	HDAC2	Class-I type histone deacetylase, Vegetative growt
Os02g0273000	UP	-	Protein kinase, catalytic domain domain containing protein
Os02g0273800	DOWN	-	Conserved hypothetical protein
Os02g0273900	DOWN	Os1900	Cytochrome P450 family member, Homolog of Arabidopsis MORE AXILLARY GROWTH 1 (MAX1
Os02g0277700	DOWN	-	Ndr family protein
Os02g0281000	DOWN	OSENOD93B	Early nodulin
Os02g0282500	DOWN	OsRLCK70	Leucine-rich repeat domain containing protein
Os02g0286933	DOWN	-	NAD(P)-binding domain containing protein
Os02g0287900	UP	prx30	Similar to Peroxidase 2 (Fragment)
Os02g0288400	UP	-	Myb-like DNA-binding domain, SHAQKYF class domain containing protein
Os02g0289700	UP	-	Conserved hypothetical protein
Os02g0306401	DOWN	PEPC-1	Similar to Phosphoenolpyruvate carboxylase 1 (EC 4.1.1.31) (PEPCase 1) (CP21)
Os02g0318100	UP	OsEFCAX2	Similar to calcium ion binding protein
Os02g0320300	DOWN	OsVQ6	VQ domain containing protein
Os02g0323600	UP	OsVQ7	Similar to Tobacco rattle virus-induced protein variant 2
Os02g0326700	DOWN	FLL1	Conserved hypothetical protein
Os02g0332450	UP	-	Non-protein coding transcript
Os02g0332700	DOWN	OsRPL21	Similar to 50S ribosomal protein L21, chloroplast precursor (CL21) (CS-L7)
Os02g0433900	UP	SIP7	Hypothetical conserved gene
Os02g0453500	DOWN	UBC5B	Ubiquitin-conjugating enzyme E2, Elicitor-induced defense respons
Os02g0460200	DOWN	-	Dynein light chain, type 1 family protein
Os02g0462401	DOWN	OsSub17	Similar to Subtilase
Os02g0467200	UP	-	Similar to Uridine kinase-like protein
Os02g0467300	UP	-	Multi antimicrobial extrusion protein MatE domain containing protein
Os02g0467400	UP	-	Conserved hypothetical protein
Os02g0470000	DOWN	-	Leucine-rich repeat domain containing protein
Os02g0471100	UP	OsPP23	RmlC-like jelly roll fold domain containing protein
Os02g0474000	DOWN	-	Disease resistance protein domain containing protein

Os02g0486500	DOWN	-	Conserved hypothetical protein
Os02g0493300	DOWN	OsFbox080	Cyclin-like F-box domain containing protein
Os02g0494400	DOWN	OsABCC5	Similar to MRP-like ABC transporter
Os02g0496900	UP	-	Hypothetical protein
Os02g0498666	DOWN	OsNAAT1	Similar to Nicotianamine aminotransferase A
Os02g0503800	UP	-	Similar to Alternative oxidase
Os02g0503850	UP	-	Similar to Chalcone isomerase 3
Os02g0505700	DOWN	Os-CYP51H4	Similar to Cytochrome P450-like protein
Os02g0508000	UP	OsRhmbd6	Peptidase S54, rhomboid domain containing protein
Os02g0508650	UP	-	Hypothetical conserved gene
Os02g0510600	DOWN	-	Conserved hypothetical protein
Os02g0513800	DOWN	-	Conserved hypothetical protein
Os02g0527900	UP	-	Similar to mitochondrial glycoprotein
Os02g0528900	UP	-	Similar to OSIGBa0131F24.5 protein
Os02g0530300	UP	-	Conserved hypothetical protein
Os02g0532300	UP	-	Hypothetical protein
Os02g0534400	UP	-	Conserved hypothetical protein
Os02g0536300	DOWN	-	Hypothetical protein
Os02g0536500	UP	-	Pentatricopeptide repeat domain containing protein
Os02g0539200	DOWN	-	Conserved hypothetical protein
Os02g0540700	DOWN	OsFbox088	F-box associated interaction domain domain containing protein
Os02g0557800	UP	-	Hypothetical conserved gene
Os02g0566300	DOWN	-	Tetratricopeptide-like helical domain containing protein
Os02g0567800	UP	-	Similar to TPR Domain containing protein, expressed
Os02g0568700	UP	-	Mitochondrial import receptor, TOM9-2 subunit, plant domain containing protein
Os02g0570400	DOWN	-	Non-protein coding transcript
Os02g0571800	UP	-	Cytochrome P450 domain containing protein
Os02g0571900	UP	-	Similar to Cytochrome P450 CYP81L6
Os02g0572050	UP	-	Hypothetical protein
Os02g0573500	DOWN	-	Trimeric LpxA-like domain containing protein
Os02g0579600	UP	-	Non-protein coding transcript
Os02g0579800	DOWN	-	Heavy metal transport/detoxification protein domain containing protein
Os02g0581400	DOWN	-	Ovarian tumour, otubain domain containing protein
Os02g0581500	DOWN	-	Hypothetical conserved gene
Os02g0584700	DOWN	ABCG41	Similar to PDR-like ABC transporter
Os02g0584800	DOWN	OsSAP5	Zinc finger, AN1-type domain containing protein

Os02g0585200	DOWN	-	Hypothetical conserved gene
Os02g0588500	DOWN	OSCIN1	Cell wall invertase (EC 3.2.1.26)
Os02g0594900	DOWN	OsFbox09 3	Leucine-rich repeat 2 domain containing protein
Os02g0596700	UP	-	Similar to H0622F05.5 protein
Os02g0597200	DOWN	OsPUB33	Zinc finger, RING/FYVE/PHD-type domain containing protein
Os02g0601300	UP	OsPUB45	Similar to Arm repeat-containing protein
Os02g0602400	DOWN	OsZFP6	Conserved hypothetical protein
Os02g0606600	UP	-	Protein kinase, catalytic domain domain containing protein
Os02g0608700	UP	OSRR2	A-type response regulator, Cytokinin signalin
Os02g0609900	DOWN	-	Conserved hypothetical protein
Os02g0611800	UP	-	Alpha/beta hydrolase fold-3 domain containing protein
Os02g0613900	UP	OsTPS5	Terpene synthase-like domain containing protein
Os02g0614200	UP	OsKS7	Similar to Ent-kaurene synthase 1A
Os02g0615400	UP	OsKS6	Terpene synthase-like domain containing protein
Os02g0616600	UP	CYP76M6	Cytochrome P450 family protein
Os02g0617600	DOWN	-	Similar to Ent-cassa-12,15-diene synthase
Os02g0618000	UP	-	Similar to Monosaccharide transporter 1
Os02g0620200	UP	Os-MADS27	Transcription factor MADS27
Os02g0621300	UP	OsFWL2	Similar to Fw2.2
Os02g0621800	UP	-	Conserved hypothetical protein
Os02g0624300	DOWN	-	Alpha/beta hydrolase fold-1 domain containing protein
Os02g0631601	DOWN	-	Heavy metal transport/detoxification protein domain containing protein
Os02g0632900	DOWN	-	Heavy metal transport/detoxification protein domain containing protein
Os02g0633066	DOWN	-	Heavy metal transport/detoxification protein domain containing protein
Os02g0635200	UP	-	Hypothetical conserved gene
Os02g0642200	UP	-	Glycosyl transferase, family 17 protein
Os02g0649800	UP	-	Similar to protein binding protein
Os02g0650900	UP	-	Conserved hypothetical protein
Os02g0654700	DOWN	OsGapC1	Similar to Glyceraldehyde-3-phosphate dehydrogenase, cytosolic 3 (EC 1.2.1.12)
Os02g0661100	DOWN	-	Mitochodrial transcription termination factor-related family protein
Os02g0664100	DOWN	OsCML17	Hypothetical conserved gene
Os02g0666250	UP	-	Similar to predicted protein
Os02g0669700	UP	-	Leucine-rich repeat, N-terminal domain containing protein
Os02g0678200	DOWN	OsHCT2	Similar to Hydroxyanthranilate hydroxycinnamoyltransferase 3
Os02g0681632	DOWN	-	Similar to Adenine phosphoribosyltransferase 2 (EC 2.4.2.7) (APRT)
Os02g0682600	UP	-	Conserved hypothetical protein
Os02g0684000	UP	-	Leucine-rich repeat domain containing protein
Os02g0687200	DOWN	-	Hypothetical protein

Os02g0691500	UP	-	Protein phosphatase 2A, regulatory subunit PR55 domain containing protein
Os02g0694800	DOWN	-	Non-protein coding transcript
Os02g0696700	UP	-	Conserved hypothetical protein
Os02g0698000	DOWN	OsCER1	Homologous protein of CER1, Very-long-chain (VLC) alkane biosynthesis, Regulation of anther development and plastids differentiatio
Os02g0703600	DOWN	HWH1	Glucose-methanol-choline (GMC) oxidoreductase family protein, Hybrid breakdown (HB
Os02g0710102	DOWN	OsMYB30	R2R3-type MYB transcription factor, Negative regulation of cold tolerance, Maintenance of Pi homeostasi
Os02g0710900	UP	-	Similar to predicted protein
Os02g0724500	DOWN	OsWAK15	Conserved hypothetical protein
Os02g0725200	DOWN	OsWAK16	Protein kinase, catalytic domain domain containing protein
Os02g0727100	DOWN	NIT1	Similar to Nitrilase 2 (EC 3.5.5.1)
Os02g0727300	DOWN	OsREM1.4	Similar to Remorin
Os02g0728500	DOWN	OsALDH3 E2	Similar to Fatty aldehyde dehydrogenase 1
Os02g0730000	UP	-	Similar to Cytochrome B5 homolog (Fragment)
Os02g0731400	DOWN	OsGDH3	Similar to Glutamate dehydrogenase 2 (EC 1.4.1.3) (GDH 2)
Os02g0731500	DOWN	ERF91	AP2/ERF family protein, Abiotic stress respons
Os02g0734300	DOWN	OsTPP1	Similar to Trehalose-6-phosphate phosphatase
Os02g0735800	DOWN	-	Hypothetical conserved gene
Os02g0736400	UP	-	Hypothetical gene
Os02g0736900	DOWN	-	Nucleotide-binding, alpha-beta plait domain containing protein
Os02g0737200	DOWN	OsSPX-MFS2	SPX-MFS protein, Pi homeostasi
Os02g0738100	DOWN	OsRLCK8 3	Peptidoglycan-binding Lysin subgroup domain containing protein
Os02g0739000	DOWN	-	Zinc finger, Tim10/DDP-type family protein
Os02g0739100	DOWN	-	Tetratricopeptide-like helical domain containing protein
Os02g0740500	UP	-	Protein of unknown function DUF581 family protein
Os02g0741500	DOWN	OsbHLH0 49	Similar to OSIGBa0142I02-OSIGBa0101B20.1 protein
Os02g0741700	UP	-	Similar to ATP binding / protein kinase
Os02g0744000	DOWN	JMJ707	Transcription factor jumonji/aspartyl beta-hydroxylase domain containing protein
Os02g0748800	DOWN	OsPrk	Similar to Phosphoribulokinase, chloroplast precursor (EC 2.7.1.19) (Phospho-pentokinase) (PRKase) (PRK)
Os02g0753300	UP	OsABA8O X1	Similar to Abscisic acid 8'-hydroxylase 1
Os02g0754300	DOWN	-	Hypothetical conserved gene
Os02g0755900	UP	-	Heat shock protein Hsp70 family protein

Os02g0757100	DOWN	-	Concanavalin A-like lectin/glucanase domain containing protein
Os02g0759400	DOWN	OsSTA72	Similar to transporter-related
Os02g0759900	DOWN	OsSET12	SET domain containing protein
Os02g0761200	UP	OsATL3	Amino acid transporter, transmembrane domain containing protein
Os02g0769800	DOWN	OsPUB21	Similar to Beta-catenin repeat family protein
Os02g0773800	UP	OsSERL3	Similar to BRASSINOSTEROID INSENSITIVE 1-associated receptor kinase 1
Os02g0774300	UP	OsALDH2a	Similar to Mitochondrial aldehyde dehydrogenase
Os02g0777400	DOWN	OsUCL5	Similar to Chemocyanin precursor (Basic blue protein) (Plantacyanin)
Os02g0781900	UP	-	Similar to cDNA, clone: J065098J18, full insert sequence
Os02g0783625	UP	-	Similar to Nudix hydrolase 18, mitochondrial precursor (EC 3.6.1.-) (AtNUDT18)
Os02g0790500	DOWN	-	Pentatricopeptide repeat domain containing protein
Os02g0791500	DOWN	OsDHODH1	Aldolase-type TIM barrel domain containing protein
Os02g0794400	DOWN	-	Conserved hypothetical protein
Os02g0794600	UP	-	Conserved hypothetical protein
Os02g0798200	UP	-	Conserved hypothetical protein
Os02g0802500	UP	-	DEAD-like helicase, N-terminal domain containing protein
Os02g0805800	DOWN	OsFH16	Similar to Formin-like protein 16
Os02g0806350	DOWN	-	Conserved hypothetical protein
Os02g0807000	DOWN	-	Ribbon-helix-helix domain containing protein
Os02g0807100	DOWN	-	Conserved hypothetical protein
Os02g0807900	DOWN	-	Similar to NDF6 (NDH DEPENDENT FLOW 6)
Os02g0811800	UP	OsFbox109	Similar to VMP3 protein
Os02g0813083	UP	OsGRL4	Thioredoxin fold domain containing protein
Os02g0813166	UP	-	Lipoxygenase, LH2 domain containing protein
Os02g0817900	DOWN	-	Ribosomal protein L29 family protein
Os02g0821400	DOWN	-	UDP-glucuronosyl/UDP-glucosyltransferase domain containing protein
Os02g0824000	DOWN	OsPHI-1	Similar to Phi-1 protein
Os02g0824700	UP	OsRING-1	Zinc finger, RING/FYVE/PHD-type domain containing protein
Os02g0827600	DOWN	-	Similar to threonine endopeptidase
Os02g0828500	UP	-	Armadillo-like helical domain containing protein
Os02g0828900	DOWN	-	Similar to Mitogen-activated protein kinase kinase kinase 1 (EC 2.7.1.-) (Arabidospsis NPK1-related protein kinase 1). Splice isoform 1S
Os02g0830801	DOWN	-	Conserved hypothetical protein
Os02g0832400	DOWN	OsISC23	Similar to Heat shock 70 kDa protein, mitochondrial precursor
Os02g0833100	DOWN	OsRLCK85	Similar to ER (ERECTA); transmembrane receptor protein kinase
Os03g0102000	UP	OsSTA81	Conserved hypothetical protein

Os03g0105600	UP	-	Similar to Lysine ketoglutarate reductase/saccharopine dehydrogenase
Os03g0106000	DOWN	OsTPS5	Hypothetical protein
Os03g0108600	DOWN	OsUGlcAE3	Similar to Nucleotide sugar epimerase-like protein (UDP-D-glucuronate 4-epimerase) (EC 5.1.3.6)
Os03g0109300	UP	-	Similar to Translation initiation factor IF-3 (Fragment)
Os03g0111200	DOWN	-	Similar to Copper chaperone COX17-1
Os03g0111400	UP	-	Zinc finger, RING/FYVE/PHD-type domain containing protein
Os03g0112600	DOWN	-	Similar to H(+)-translocating (Pyrophosphate-ENERGIZED) inorganic pyrophosphatase beta-1 polypeptide (EC 3.6.1.1) (Fragment)
Os03g0113500	DOWN	-	Aminoglycoside phosphotransferase domain containing protein
Os03g0115300	DOWN	-	Hypothetical protein
Os03g0115800	DOWN	OsPPCK1	Similar to Phosphoenolpyruvate carboxylase kinase 1
Os03g0118450	UP	-	Similar to Starch synthase V
Os03g0118600	DOWN	OsWAK21	Conserved hypothetical protein
Os03g0121700	DOWN	OsCCR10	Similar to Cinnamoyl-CoA reductase (EC 1.2.1.44)
Os03g0122200	DOWN	OsFbox118	Conserved hypothetical protein
Os03g0123500	UP	OsFbox119	Cyclin-like F-box domain containing protein
Os03g0126000	UP	-	Armadillo-type fold domain containing protein
Os03g0126700	UP	CYP97A4	Cytochrome P450 family protein
Os03g0128200	DOWN	OsRLCK90	Protein kinase, core domain containing protein
Os03g0130300	UP	-	Hypothetical conserved gene
Os03g0130900	DOWN	-	Similar to Vacuolar ATP synthase subunit F (EC 3.6.3.14) (V-ATPase F subunit) (Vacuolar proton pump F subunit) (V-ATPase 14 kDa subunit)
Os03g0132900	DOWN	-	Protein of unknown function DUF3531 domain containing protein
Os03g0133000	DOWN	-	Tetratricopeptide-like helical domain containing protein
Os03g0133300	DOWN	JMJ720	Transcription factor jumonji domain containing protein
Os03g0135300	DOWN	-	Similar to binding
Os03g0136900	UP	OsGSL7	Similar to Callose synthase 1 catalytic subunit
Os03g0138900	DOWN	OsFRDL3	Similar to antiporter/ drug transporter
Os03g0140700	DOWN	-	Conserved hypothetical protein
Os03g0141200	DOWN	OsTUB2	Tubulin beta-1 chain (Beta-1 tubulin)
Os03g0146200	UP	-	Uncharacterised protein family UPF0187 domain containing protein
Os03g0148400	DOWN	OsRH50	Similar to DEAD-box ATP-dependent RNA helicase 50
Os03g0150600	DOWN	LOGL3	Similar to Lysine decarboxylase-like protein
Os03g0152700	DOWN	OsREM1.3	Similar to Remorin
Os03g0153100	UP	-	Heavy metal transport/detoxification protein domain containing protein
Os03g0159100	UP	-	Similar to Plus-3 domain containing protein, expressed
Os03g0169600	UP	-	Conserved hypothetical protein

Os03g0170100	DOWN	-	Pentatricopeptide repeat domain containing protein
Os03g0178200	DOWN	-	Conserved hypothetical protein
Os03g0179950	UP	-	Non-protein coding transcript
Os03g0181800	DOWN	-	Similar to Dihydrodipicolinate reductase-like protein
Os03g0184300	UP	-	Similar to Nascent polypeptide-associated complex alpha subunit-like protein 3 (NAC-alpha-like protein 3) (Alpha-NAC-like protein 3)
Os03g0187550	DOWN	-	Similar to 50S ribosomal protein L11
Os03g0188100	UP	HB365	Similar to HOS66 protein
Os03g0188500	DOWN	-	Similar to Phosphorybosyl anthranilate transferase 1
Os03g0189100	DOWN	-	Similar to Barley stem rust resistance protein
Os03g0190400	DOWN	OsGSL10	Similar to predicted protein
Os03g0198300	DOWN	DEFL8	Similar to Cp-thionin
Os03g0207400	DOWN	OsRLCK96	Serine/threonine protein kinase domain containing protein
Os03g0213100	UP	Cht11	Similar to Chitinase 11
Os03g0218300	DOWN	NAC22	NAC transcription factor, Drought and salt stress tolerance, Modulation of ABA-mediated pathwa
Os03g0221200	UP	-	Similar to F7F22.5
Os03g0221800	DOWN	OsGSTF3	Similar to Glutathione S-transferase GST 10 (EC 2.5.1.18)
Os03g0225900	DOWN	-	Similar to Aconitate hydratase, cytoplasmic (EC 4.2.1.3) (Citrate hydro-lyase) (Aconitase)
Os03g0226200	UP	-	Conserved hypothetical protein
Os03g0231800	UP	-	Tetratricopeptide-like helical domain containing protein
Os03g0232400	DOWN	OsBamy2	Similar to Beta-amylase PCT-BMYI (EC 3.2.1.2)
Os03g0233900	UP	-	Conserved hypothetical protein
Os03g0234500	DOWN	-	Protein of unknown function DUF740 family protein
Os03g0235700	UP	PT1	Pi transporter, Pi homeostasi
Os03g0242300	UP	-	Pseudouridine synthase domain containing protein
Os03g0243100	UP	-	Similar to FAD binding domain containing protein, expressed
Os03g0249100	DOWN	-	Phosphoserine aminotransferase, chloroplast precursor (EC 2.6.1.52) (PSAT)
Os03g0254000	DOWN	OsRLCK101	Similar to Protein kinase APK1B, chloroplast precursor (EC 2.7.1.-)
Os03g0254200	DOWN	RDD4	Dof (DNA binding with one finger) transcription factor, Regulation of Flowering time, Brassinosteroid (BR) signaling to modulate plant architectur
Os03g0254800	UP	-	Conserved hypothetical protein
Os03g0255100	UP	-	Protein of unknown function YGGT family protein
Os03g0257600	UP	-	Non-protein coding transcript
Os03g0259600	DOWN	-	Protein of unknown function DUF936, plant family protein
Os03g0261100	UP	-	Conserved hypothetical protein
Os03g0265400	DOWN	-	Hypothetical gene
Os03g0265800	UP	OsMATE1	Similar to transparent testa 12 protein

Os03g0271100	DOWN	OsAGP3	Glutelin family protein
Os03g0271400	DOWN	-	Uncharacterised protein family UPF0503 domain containing protein
Os03g0271500	DOWN	-	Hypothetical gene
Os03g0272400	DOWN	OsDjC25	Heat shock protein DnaJ, N-terminal domain containing protein
Os03g0272900	DOWN	OsPP2C29	Similar to Protein phosphatase 2C-like
Os03g0275300	DOWN	-	Similar to Sec61p
Os03g0275450	DOWN	-	Conserved hypothetical protein
Os03g0277700	UP	-	Similar to Homocysteine S-methyltransferase 1 (EC 2.1.1.10) (S- methylmethionine:homocysteine methyltransferase 1) (SMM:Hcy S- methyltransferase 1) (ZmHMT-1)
Os03g0279200	DOWN	-	Similar to Leucine Rich Repeat family protein, expressed
Os03g0284500	DOWN	-	Cytochrome P450 family protein
Os03g0285800	DOWN	AOS2	Allene oxide synthase (CYP74A2), Biosynthesis of jasmonic acid (JA
Os03g0289400	UP	HB2	Non-symbiotic hemoglobin 2 (rHb2
Os03g0294100	UP	-	Similar to Squalene monooxygenase 1 (EC 1.14.99.7)
Os03g0298300	DOWN	-	Phytosulfokine family protein
Os03g0301200	DOWN	HB1	Non-symbiotic hemoglobin 1 (rHb1
Os03g0302200	DOWN	prx37	Haem peroxidase, plant/fungal/bacterial family protein
Os03g0309800	DOWN	poxN	Similar to Peroxidase
Os03g0310800	DOWN	-	Similar to Peptide transporter 1
Os03g0319400	DOWN	-	Hypothetical protein
Os03g0321700	UP	OsADF5	Similar to Actin-depolymerizing factor 5 (ADF-5) (AtADF5)
Os03g0324600	DOWN	-	Conserved hypothetical protein
Os03g0327800	DOWN	-	NB-ARC domain containing protein
Os03g0341100	UP	-	Similar to predicted protein
Os03g0341300	UP	CS	Similar to Chorismate synthase 1, chloroplast precursor (EC 4.2.3.5) (5-enolpyruvylshikimate-3-phosphate phospholyase 1)
Os03g0348200	UP	OsBgal8	Similar to Relative to SR12 protein (Fragment)
Os03g0351300	DOWN	-	Hypothetical conserved gene
Os03g0352450	DOWN	-	Ubiquitin domain containing protein
Os03g0353400	DOWN	PLA2-II	Phospholipase A2 family protein
Os03g0356300	DOWN	-	Similar to 50S ribosomal protein L4, chloroplast precursor (R-protein L4)
Os03g0364400	DOWN	-	Protein of unknown function DUF2253, membrane domain containing protein
Os03g0364700	DOWN	OsSIG2B	Similar to Sigma factor SIG2B
Os03g0367101	DOWN	-	Armadillo-like helical domain containing protein
Os03g0377700	DOWN	-	Conserved hypothetical protein
Os03g0379500	UP	-	Conserved hypothetical protein
Os03g0386000	UP	-	DVL family protein
Os03g0387300	UP	-	Hypothetical conserved gene
Os03g0390400	UP	-	Hypothetical protein
Os03g0400700	UP	-	Protein of unknown function DUF26 domain containing protein

Os03g0406100	UP	-	Similar to Histone H2A
Os03g0412800	DOWN	-	HAD-superfamily subfamily IIA hydrolase, CECR5 protein
Os03g0418700	DOWN	OsMSRMK2	MAP Kinase
Os03g0422800	DOWN	OsStr2	Rhodanese-like domain containing protein
Os03g0431200	UP	SIP32	Similar to pherophorin like protein
Os03g0438400	UP	-	Uncharacterised protein family UPF0497, trans-membrane plant domain containing protein
Os03g0448600	UP	-	COBRA-like protein 7 precursor
Os03g0563300	DOWN	OsRFP	Zinc finger, RING/FYVE/PHD-type domain containing protein
Os03g0570100	UP	-	Similar to PPR2
Os03g0571700	UP	-	Similar to Hypersensitive reaction associated Ca2+-binding protein
Os03g0571900	UP	CK1	Serine/threonine protein kinase, Cold stress tolerance, Abiotic stresse
Os03g0573001	DOWN	OsWRKY55	Similar to WRKY transcription factor 55
Os03g0575200	DOWN	-	NB-ARC domain containing protein
Os03g0593600	UP	NAC58	NAC Family transcriptional activator, Abiotic stress response, Positive regulator of leaf senescenc
Os03g0597200	DOWN	-	Similar to 60S ribosomal protein L18
Os03g0599000	UP	OsSWEET16	Similar to Cytochrome c oxidoreductase
Os03g0602600	DOWN	OsTPS10	Terpene synthase-like domain containing protein
Os03g0606100	DOWN	-	Similar to beta-amylase
Os03g0606200	UP	-	Conserved hypothetical protein
Os03g0607700	UP	OsINO80	Hypothetical conserved gene
Os03g0617500	DOWN	-	Similar to Poly(A)-binding protein C-terminal interacting protein 6
Os03g0622600	UP	-	Ribosomal protein L6 family protein
Os03g0626500	UP	OsBBS1	Receptor-like cytoplasmic kinase, Leaf senescence, Salt stress respons
Os03g0634400	UP	-	Transcription elongation factor, TFIIS/CRSP70, N-terminal domain containing protein
Os03g0637600	UP	Os-CYP75A11	Similar to cytochrome P450 flavonoid 3',5'-hydroxylase
Os03g0638400	UP	OsCslA5	Cellulose synthase-like A5
Os03g0648600	DOWN	-	Similar to 40S ribosomal protein S9
Os03g0650200	UP	SRWD5	Similar to Salt-responsive WD40 protein 5
Os03g0655100	UP	OsVQ12	Similar to VQ motif family protein, expressed
Os03g0656900	DOWN	Os-COX6b1	Similar to Cytochrome c oxidase subunit 6b
Os03g0660300	DOWN	OsLEA11	Similar to retrotransposon protein
Os03g0660400	DOWN	-	Similar to Protein kinase domain containing protein, expressed
Os03g0660500	DOWN	SUS1	Sucrose synthase 2 (EC 2.4.1.13) (Sucrose-UDP glucosyltransferase 2)

Os03g0661600	UP	-	Hypothetical conserved gene
Os03g0663400	UP	OsSPX5	SPX domain-containing protein, Negative regulation of phosphate signaling, Pi homeostasi
Os03g0663500	UP	OsG6PDH4	Similar to Glucose-6-phosphate dehydrogenase precursor
Os03g0667100	DOWN	-	Conserved hypothetical protein
Os03g0679700	DOWN	-	Similar to D-mannose binding lectin family protein, expressed
Os03g0682822	UP	-	Similar to OSIGBa0135L04.1 protein
Os03g0684500	DOWN	-	Similar to N-acetyl-glutamate synthase
Os03g0684800	DOWN	-	Hypothetical protein
Os03g0685500	UP	OsWD40-76	WD40 repeat-like domain containing protein
Os03g0685700	DOWN	ELL	Magnesium-chelatase subunit ChlI, Mg-chelatase I subunit, Chlorophyll biosynthesis, Chloroplast developmen
Os03g0685900	UP	-	Conserved hypothetical protein
Os03g0686300	DOWN	-	Similar to Cytochrome P450 79A1 (EC 1.14.13.41) (Tyrosine N-monooxygenase) (Cytochrome P450Tyr)
Os03g0693900	UP	-	Similar to TRANSPARENT TESTA 12 protein
Os03g0694000	UP	PEZ1	Similar to transparent testa 12 protein
Os03g0701300	DOWN	-	Conserved hypothetical protein
Os03g0704000	DOWN	OsHAK16	Similar to Potassium transporter
Os03g0710000	DOWN	CYP71W1	Similar to Cytochrome P450 family protein, expressed
Os03g0710600	DOWN	YSA	Pentatricopeptide repeat protein, Chloroplast developmen
Os03g0712800	UP	-	Hypothetical conserved gene
Os03g0718000	UP	-	NB-ARC domain containing protein
Os03g0718100	UP	HSA1	PfkB family fructokinase, WLP2 paralog, Chloroplast biogenesis and plant growth, Protection of chloroplasts under heat stres
Os03g0718800	UP	-	Similar to predicted protein
Os03g0719400	UP	RMtATP6	Mitochondrial ATP synthase 6 KD subunit
Os03g0722400	DOWN	-	Zinc finger, C2H2-like domain containing protein
Os03g0726200	UP	CycD5	Cyclin, C-terminal domain containing protein
Os03g0727600	DOWN	-	Conserved hypothetical protein
Os03g0728900	DOWN	-	Similar to predicted protein
Os03g0734200	UP	OsCIPK07	Serine/threonine protein kinase domain containing protein
Os03g0734300	UP	-	Leucine-rich repeat, plant specific containing protein
Os03g0736300	UP	-	Similar to OSIGBa0153E02-OSIGBa0093I20.19 protein
Os03g0736400	DOWN	-	Similar to Zinc finger, C3HC4 type family protein, expressed
Os03g0739700	DOWN	-	Similar to Cytochrome P450 CYP92C5
Os03g0744600	UP	-	Similar to Cupin, RmlC-type
Os03g0744700	DOWN	NUS1	Plastid RNA-binding protein, Regulation of chloroplast RNA metabolism, Early chloroplast development under cold stres

Os03g0747700	DOWN	Os-SAUR15	Auxin responsive SAUR protein domain containing protein
Os03g0748300	DOWN	Os-SAUR16	Hypothetical conserved gene
Os03g0749300	UP	Os-SAUR17	Auxin responsive SAUR protein domain containing protein
Os03g0752300	DOWN	OsPR5	Similar to Alpha-amylase/trypsin inhibitor (Antifungal protein)
Os03g0752400	DOWN	OsLP	Similar to Thaumatin-like protein
Os03g0757100	UP	TLP	Similar to Osmotin precursor
Os03g0759700	UP	OsNPR3	Similar to NPR1-like protein
Os03g0760200	UP	OsTHIC	Similar to Thiamine biosynthesis protein thiC
Os03g0764000	UP	-	Hypothetical protein
Os03g0764600	UP	-	Similar to glycosyltransferase family protein 47
Os03g0769100	DOWN	-	Conserved hypothetical protein
Os03g0773300	DOWN	-	CHCH domain containing protein
Os03g0776150	DOWN	-	Conserved hypothetical protein
Os03g0777700	DOWN	-	Similar to F1N21.17
Os03g0781000	DOWN	-	Similar to unconventional myosin heavy chain
Os03g0783700	UP	OsOxO3	Similar to Oxalate oxidase 1 (EC 1.2.3.4) (Germin)
Os03g0784600	DOWN	Osoxo4	Similar to Oxalate oxidase 1 (EC 1.2.3.4) (Germin)
Os03g0789466	DOWN	-	Similar to predicted protein
Os03g0794200	UP	-	Similar to 30S ribosomal protein S13
Os03g0795200	DOWN	-	Protein of unknown function DUF292, eukaryotic domain containing protein
Os03g0799300	UP	-	Conserved hypothetical protein
Os03g0799700	DOWN	OsGS1	Cytosolic glutamine synthetas
Os03g0807400	DOWN	OASB2	Similar to Anthranilate synthase beta chain
Os03g0811100	DOWN	ACT1	Actin 1
Os03g0811400	UP	-	Similar to Physical impedance induced protein
Os03g0811800	UP	-	Alpha/beta hydrolase fold-1 domain containing protein
Os03g0815100	DOWN	OsDDM1b	Chromatin remodeling factor, Genome-wide DNA methylatio
Os03g0816200	UP	OsITPK3	Similar to Hydroxyproline-rich glycoprotein DZ-HRGP precursor
Os03g0816950	DOWN	ACS1	ACC synthase, Ethylene biosynthesi
Os03g0817200	UP	OsbHLH084	Helix-loop-helix DNA-binding domain containing protein
Os03g0821900	DOWN	-	Conserved hypothetical protein
Os03g0824100	DOWN	-	Similar to Proteinase inhibitor type II CEVI57 precursor
Os03g0825500	DOWN	OsGH9A3	Similar to CEL6=CELLULASE 6 (Fragment)
Os03g0826800	DOWN	-	Protein of unknown function methylase putative domain containing protein
Os03g0827700	DOWN	-	Uncharacterised protein family UPF0114 domain containing protein
Os03g0828100	DOWN	-	Similar to Ripening-associated protein (Fragment)
Os03g0828800	DOWN	-	Conserved hypothetical protein

Os03g0830800	UP	-	Similar to photosystem II 11 kDa protein-related
Os03g0835100	DOWN	-	Aldose 1-epimerase family protein
Os03g0835150	UP	OsBGLU1	Similar to Exoglucanase precursor
Os03g0835400	UP	-	Similar to 50S ribosomal protein l6
Os03g0838800	UP	TPKA	Two-pore K+ channel family protein, K+ homeostasi
Os03g0841100	DOWN	-	Hypothetical conserved gene
Os03g0843400	DOWN	-	UDP-glucuronosyl/UDP-glucosyltransferase family protein
Os03g0844000	DOWN	OsbHLH066	Similar to ER33 protein (Fragment)
Os03g0848100	DOWN	bel	Cytochrome P450 family protein
Os03g0851500	UP	-	Acyl-CoA N-acyltransferase domain containing protein
Os03g0852700	DOWN	-	Homeodomain-like containing protein
Os03g0861700	DOWN	WGL2	Chloroplast ribosomal protein S9, Early chloroplast developmen
Os04g0102500	UP	-	Protein kinase, core domain containing protein
Os04g0103601	UP	-	Conserved hypothetical protein
Os04g0103800	DOWN	-	Hypothetical protein
Os04g0105400	UP	-	FAR1 DNA binding domain domain containing protein
Os04g0111200	UP	-	Conserved hypothetical protein
Os04g0111250	UP	-	Similar to AP-1 complex subunit sigma-2
Os04g0115700	UP	-	Conserved hypothetical protein
Os04g0118700	DOWN	OsFLA24	FAS1 domain domain containing protein
Os04g0118800	DOWN	-	Similar to Protein argonaute MEL1
Os04g0119000	DOWN	-	Similar to N-acetyltransferase
Os04g0120100	DOWN	OsGDH1	Similar to Glutamate dehydrogenase (EC 1.4.1.3) (GDH)
Os04g0121900	UP	-	Pentatricopeptide repeat domain containing protein
Os04g0126500	UP	-	Conserved hypothetical protein
Os04g0126900	UP	TSV3	Obg subfamily of small GTP-binding protein, Chloroplast development at the early leaf stage under cold stres
Os04g0127300	UP	-	Pentatricopeptide repeat domain containing protein
Os04g0131850	DOWN	OsChlD	Magnesium-chelatase subunit ChlD, Chlorophyll synthesis, Chloroplast developmen
Os04g0165700	UP	OsbHLH041	Similar to DNA binding protein
Os04g0169500	UP	-	Ribosomal protein L36 family protein
Os04g0169666	UP	SNAC1	Similar to OsNAC6 protein
Os04g0179700	UP	-	Ribosomal protein S26e domain containing protein
Os04g0181901	UP	-	Hypothetical protein
Os04g0194433	UP	OsANT2	Amino acid transporter, transmembrane domain containing protein
Os04g0194500	DOWN	OsRLCK118	Similar to Protein kinase APK1A, chloroplast precursor (EC 2.7.1.-)
Os04g0202500	DOWN	OspTAC2	Pentatricopeptide repeat protein, Regulation of chloroplast developmen

Os04g0204400	DOWN	-	Transcription antitermination protein, NusG, N-terminal domain containing protein
Os04g0206700	UP	-	Conserved hypothetical protein
Os04g0209200	UP	OsRH3	Similar to ATP-dependent RNA helicase
Os04g0218600	DOWN	-	Similar to 50S ribosomal protein L18, chloroplastic
Os04g0219600	DOWN	-	Serine/threonine protein kinase-related domain containing protein
Os04g0233400	DOWN	-	Pectinesterase inhibitor domain containing protein
Os04g0247700	DOWN	-	Similar to Chloroplast protein import component Toc159
Os04g0253000	DOWN	-	Conserved hypothetical protein
Os04g0284900	UP	-	Similar to Uvs101
Os04g0293100	DOWN	-	Zinc finger, C2H2 domain containing protein
Os04g0293300	UP	OsWAK28	Serine/threonine protein kinase-related domain containing protein
Os04g0301500	DOWN	-	Similar to 30S ribosomal protein S6, chloroplast precursor (Fragment)
Os04g0304250	DOWN	OsHOL1	Methyl chloride transferase
Os04g0316200	UP	-	Pentatricopeptide repeat domain containing protein
Os04g0320700	DOWN	LDC	Similar to Ser/Thr protein phosphatase family protein, expressed
Os04g0322100	UP	OsRad52-2a	Homolog of Rad52 (Radiation sensitive 52), Mediation of homologous recombination and DNA repair
Os04g0326300	UP	-	Pentatricopeptide repeat domain containing protein
Os04g0337000	DOWN	-	Rhodanese-like domain containing protein
Os04g0337201	DOWN	-	Similar to OSIGBa0123D13.3 protein
Os04g0339400	UP	-	Conserved hypothetical protein
Os04g0346800	UP	-	Hypothetical protein
Os04g0360500	UP	Os-COMTL2	Similar to 0-methyltransferase (EC 2.1.1.6) (Fragment)
Os04g0366000	DOWN	-	Similar to B0616E02-H0507E05.3 protein
Os04g0367000	DOWN	-	Similar to ATP sulfurylase (Fragment)
Os04g0368000	DOWN	-	Conserved hypothetical protein
Os04g0369600	UP	-	Hypothetical protein
Os04g0370500	DOWN	-	Pentatricopeptide repeat domain containing protein
Os04g0372700	DOWN	-	NB-ARC domain containing protein
Os04g0372750	DOWN	-	Similar to H0716A07.5 protein
Os04g0376300	UP	OsSLPP1	Similar to H0716A07.11 protein
Os04g0380300	DOWN	-	Hypothetical conserved gene
Os04g0385600	DOWN	-	Hypothetical conserved gene
Os04g0402100	UP	-	Hypothetical protein
Os04g0404400	UP	-	Similar to H0124E07.4 protein
Os04g0405300	UP	OsSub38	Peptidase S8 and S53, subtilisin, kexin, sedolisin domain containing protein
Os04g0412300	DOWN	-	Similar to UDP-glucose:sterol glucosyltransferase
Os04g0415200	UP	OsCAS	Cysteine synthase (EC 2.5.1.47)
Os04g0417600	DOWN	-	Similar to Avr9/Cf-9 rapidly elicited protein 137

Os04g0417800	DOWN	-	Hypothetical protein
Os04g0418000	UP	OsKS4	9-beta-pimara-7,15-diene synthase, Momilactone phytoalexins biosynthesis, Defense respons
Os04g0418800	UP	-	Hypothetical conserved gene
Os04g0419100	UP	-	2OG-Fe(II) oxygenase domain containing protein
Os04g0419400	DOWN	-	Hypothetical gene
Os04g0419900	DOWN	OsABCG8	Similar to ABC transporter-like protein
Os04g0420700	DOWN	OsRLCK135	Similar to H0512B01.12 protein
Os04g0420801	DOWN	-	Hypothetical protein
Os04g0432600	UP	-	UDP-glucuronosyl/UDP-glucosyltransferase family protein
Os04g0439400	DOWN	OsABCC9	Similar to MRP-like ABC transporter
Os04g0442300	UP	-	Conserved hypothetical protein
Os04g0444800	DOWN	rNBS17	Similar to NBS-LRR protein (Fragment)
Os04g0445700	DOWN	OsPIP2	Similar to Arabidopsis thaliana (Fragment)
Os04g0448300	UP	-	Similar to H0315A08.1 protein
Os04g0448800	UP	H1	Similar to Histone H1
Os04g0452700	DOWN	-	Similar to OSIGBa0145C02.7 protein
Os04g0460300	UP	-	Similar to Auxin-binding protein ABP20
Os04g0464800	DOWN	-	Hypothetical protein
Os04g0469000	DOWN	-	Similar to Tropinone reductase-I (EC 1.1.1.206) (TR-I) (Tropine dehydrogenase)
Os04g0469700	DOWN	OsbHLH006	Helix-loop-helix DNA-binding domain containing protein
Os04g0469800	DOWN	-	Hypothetical protein
Os04g0474300	UP	-	Protein of unknown function DUF26 domain containing protein
Os04g0474800	UP	-	Similar to Glucosyltransferase (Fragment)
Os04g0482900	UP	-	Protein of unknown function DUF26 domain containing protein
Os04g0483200	DOWN	-	Hypothetical conserved gene
Os04g0488100	DOWN	OsSTA120	Peptidase aspartic, catalytic domain containing protein
Os04g0490500	DOWN	-	Similar to OSIGBa0137O04.8 protein
Os04g0494100	UP	-	Aldo/keto reductase family protein
Os04g0494300	DOWN	OsAKR	Aldo/keto reductase family protein
Os04g0511200	UP	-	Similar to OSIGBa0111I14.3 protein
Os04g0513400	UP	-	Similar to OSIGBa0111E13.1 protein
Os04g0514600	DOWN	-	Similar to OSIGBa0110B10.6 protein
Os04g0515400	UP	-	Hypothetical conserved gene
Os04g0517100	DOWN	OsWAK43	Serine/threonine protein kinase-related domain containing protein
Os04g0517700	UP	-	Similar to OSIGBa0107E14.3 protein
Os04g0519700	DOWN	Os_F0607	Similar to OSIGBa0107E14.10 protein
Os04g0524600	DOWN	-	Similar to Quinone-oxidoreductase QR1 (Fragment)

Os04g0527400	UP	-	Non-protein coding transcript
Os04g0527700	UP	-	Similar to 3-oxoacyl-reductase
Os04g0528800	UP	OsFbox203	Kelch-type beta propeller domain containing protein
Os04g0535200	UP	Os_F0796	Tetratricopeptide-like helical domain containing protein
Os04g0535600	UP	OsCNX	Similar to Calnexin (Fragment)
Os04g0538166	DOWN	OsEnS-64	Similar to H0502B11.4 protein
Os04g0544400	DOWN	Os-SDR110C-MS3	Similar to Stem secoisolariciresinol dehydrogenase (Fragment)
Os04g0550600	UP	-	Similar to H0717B12.10 protein
Os04g0551700	DOWN	-	TB2/DP1 and HVA22 related protein family protein
Os04g0555300	DOWN	OsVQ15	Similar to OSIGBa0092M08.12 protein
Os04g0556400	DOWN	OsWD40-100	WD40 repeat-like domain containing protein
Os04g0556500	DOWN	-	Conserved hypothetical protein
Os04g0557000	DOWN	-	Similar to Hydroxyproline-rich glycoprotein
Os04g0559550	UP	-	Conserved hypothetical protein
Os04g0559700	UP	-	Conserved hypothetical protein
Os04g0559750	UP	YK18	Similar to H0525E10.7 protein
Os04g0561500	DOWN	-	Hypothetical protein
Os04g0565200	UP	-	Hypothetical protein
Os04g0565500	DOWN	-	Similar to H0315E07.2 protein
Os04g0566600	UP	-	Similar to H0525C06.6 protein
Os04g0571600	DOWN	OSRR1	A-type response regulator, Cytokinin signalin
Os04g0572200	DOWN	OsFRO7	Ferric reductase-like transmembrane component family protein
Os04g0572600	DOWN	-	Similar to OSIGBa0140O07.8 protein
Os04g0583700	DOWN	-	Peptidase A1 domain containing protein
Os04g0584600	DOWN	OsPT17	Similar to Mitochondrial phosphate transporter (Fragment)
Os04g0585900	UP	OsMST1	Similar to Monosaccharide transporter 1
Os04g0586500	DOWN	OsATL13	Amino acid transporter, transmembrane domain containing protein
Os04g0588500	DOWN	-	Similar to OSIGBa0130P02.2 protein
Os04g0597501	DOWN	-	Heavy metal transport/detoxification protein domain containing protein
Os04g0601700	UP	-	Hypothetical protein
Os04g0601800	DOWN	D11	Cytochrome P450 superfamily protein, CYP724B1, Brassinosteroid (BR) biosynthesis, Control of panicle architecture and seed siz
Os04g0604300	DOWN	Os4bglu9	Glycoside hydrolase, family 1 protein
Os04g0610400	DOWN	Os4bglu12	Similar to OSIGBa0135C13.7 protein
Os04g0610500	DOWN	-	Similar to H0525D09.12 protein
Os04g0611700	UP	-	Steroid nuclear receptor, ligand-binding domain containing protein

Os04g0612000	UP	OsRad21-2	Rad21/Rec8 like protein, C-terminal domain containing protein
Os04g0614300	UP	OsRLCK152	Protein kinase-like domain domain containing protein
Os04g0615500	DOWN	Cht5	Similar to Chitinase
Os04g0616200	UP	-	Hypothetical gene
Os04g0616600	UP	EFA27	EFA27 for EF hand, abscisic acid, 27kD
Os04g0617200	DOWN	Os4bglu16	Similar to Beta-glucosidase
Os04g0617500	DOWN	-	Similar to NBS-LRR type disease resistance protein O2 (Fragment)
Os04g0617900	UP	-	NTP pyrophosphohydrolase MazG, putative catalytic core domain containing protein
Os04g0619400	DOWN	-	Similar to OSIGBa0145M07.3 protein
Os04g0619900	DOWN	OsMyb4	Similar to OSIGBa0145M07.4 protein
Os04g0621500	DOWN	OsWAK51	Similar to OSIGBa0145M07.8 protein
Os04g0623066	DOWN	OsARF10	Similar to Auxin response factor 10
Os04g0629600	DOWN	OsS40-13	Protein of unknown function DUF584 family protein
Os04g0630600	DOWN	OsYSL12	Oligopeptide transporter OPT superfamily protein
Os04g0630900	DOWN	-	BRO1 domain containing protein
Os04g0632400	DOWN	-	CHCH domain containing protein
Os04g0636300	DOWN	-	Similar to Ferredoxin-thioredoxin reductase, variable chain (FTR-V) (Ferredoxin- thioredoxin reductase subunit A) (FTR-A)
Os04g0639300	DOWN	-	Similar to OSIGBa0125M19.13 protein
Os04g0643200	DOWN	-	Peptidase A1 domain containing protein
Os04g0644900	UP	OSINV2	Similar to Beta-fructofuranosidase 1 precursor (EC 3.2.1.26) (Sucrose 1) (Invertase 1)
Os04g0645200	UP	-	Similar to Elongation factor G, chloroplastic
Os04g0647900	DOWN	-	Hypothetical conserved gene
Os04g0648400	DOWN	HTD1	Carotenoid cleavage dioxygenase, Negative regulation of the outgrowth of axillary buds, Strigolactones biosynthesi
Os04g0651000	UP	-	PAP fibrillin family protein
Os04g0657500	DOWN	FCO11	Similar to glycerol 3-phosphate permease
Os04g0660600	UP	-	Similar to UDP-glycosyltransferase UGT93B9
Os04g0664500	UP	CZOGT1	cis-Zeatin-O-glucosyltransferase, Catalyze O-glucosylation of cZ and cZ-ribosid
Os04g0667600	DOWN	-	Similar to Genetic modifier
Os04g0673300	UP	-	Conserved hypothetical protein
Os04g0674700	UP	OsPIP1	Similar to Aquaporin PIP1.1
Os04g0674800	UP	-	Hypothetical protein
Os04g0675200	DOWN	OsPOP9	Similar to Prolyl endopeptidase (EC 3.4.21.26) (Post-proline cleaving enzyme) (PE)
Os04g0678700	DOWN	-	Similar to Cis-zeatin O-glucosyltransferase 1 (EC 2.4.1.215) (cisZOG1)

Os04g0680700	DOWN	OsANT4	Similar to OSIGBa0158F05.8 protein
Os04g0684500	DOWN	-	Homeodomain-like containing protein
Os04g0684900	DOWN	-	Multi antimicrobial extrusion protein MatE family protein
Os04g0685300	DOWN	OsERF	-
Os04g0687800	UP	-	Similar to DNA-directed RNA polymerase
Os04g0691800	UP	-	Hypothetical conserved gene
Os05g0100100	UP	-	Zinc finger, FYVE-type domain containing protein
Os05g0103800	UP	Os-CDPK13	Similar to Calcium dependent protein kinase
Os05g0104200	UP	-	Protein of unknown function DUF581 family protein
Os05g0106200	UP	-	Malectin-like carbohydrate-binding domain domain containing protein
Os05g0109825	DOWN	-	Hypothetical gene
Os05g0112800	UP	-	Similar to OSJNba0093F12.10 protein
Os05g0116000	UP	-	Similar to Vacuolar ATP synthase subunit G 1 (EC 3.6.3.14) (V-ATPase G subunit 1) (Vacuolar proton pump G subunit 1)
Os05g0122800	UP	-	Similar to Plastid protein
Os05g0123000	UP	OsXTH9	Similar to Xyloglucan endotransglucosylase/hydrolase protein 24 precursor (EC 2.4.1.207) (At-XTH24) (XTH-24) (Meristem protein 5) (MERI-5 protein) (MERI5 protein) (Endo-xyloglucan transferase) (Xyloglucan endo-1,4-beta-D-glucanase)
Os05g0126400	DOWN	ERF77	APETALA-2-like transcription Factor, Control of the abscisic acid (ABA)/gibberellin (GA) balanc
Os05g0127200	UP	-	Peptidase M24A, methionine aminopeptidase, subfamily 1 protein
Os05g0127300	DOWN	OsKS3	Similar to Ent-kaurene synthase-like 3
Os05g0127400	UP	KS2	Ent-beyerene synthase, Phytoalexin biosynthesis, Defense respons
Os05g0127500	UP	-	Hypothetical conserved gene
Os05g0128100	DOWN	OsISC39	Similar to H0525G02.2 protein
Os05g0131500	DOWN	OsRLCK159	Similar to H0525G02.9 protein
Os05g0132100	DOWN	-	Serine/threonine protein kinase-related domain containing protein
Os05g0132800	UP	-	Conserved hypothetical protein
Os05g0134400	UP	-	Similar to H0313F03.4 protein
Os05g0135500	UP	OsGLP4-1	Similar to germin-like protein subfamily 1 member 11
Os05g0135700	UP	OsRLCK160	Protein kinase, core domain containing protein
Os05g0137200	DOWN	-	Similar to H0114G12.10 protein
Os05g0142400	UP	-	Disease resistance protein domain containing protein
Os05g0142500	UP	-	Similar to XA1
Os05g0145100	UP	GLO3	Similar to H0215F08.7 protein
Os05g0147500	DOWN	-	Conserved hypothetical protein
Os05g0152201	UP	OsSTA134	Similar to H0323C08.16 protein

Os05g0155100	UP	-	Similar to H0105C05.3 protein
Os05g0156500	UP	-	Hypothetical protein
Os05g0156700	DOWN	-	Similar to H0410G08.15 protein
Os05g0157500	DOWN	-	Conserved hypothetical protein
Os05g0165900	DOWN	-	Amidase family protein
Os05g0166450	UP	Os-NTMC2T2.1	C2 domain containing protein
Os05g0176600	UP	OsVQ16	VQ domain containing protein
Os05g0179100	UP	-	Leucine-rich repeat, N-terminal domain containing protein
Os05g0179300	UP	-	Similar to H0811D08.17 protein
Os05g0181300	DOWN	OsGT43I	Similar to H0212B02.9 protein
Os05g0181700	DOWN	PRX57	Similar to Peroxidase
Os05g0184901	UP	-	Lipase, class 3 family protein
Os05g0195101	DOWN	-	ATPase, V0 complex, subunit E domain containing protein
Os05g0195700	UP	Os-AHT1	Similar to H1005F08.2 protein
Os05g0202800	DOWN	-	Heavy metal transport/detoxification protein domain containing protein
Os05g0203900	DOWN	RR6	A-type response regulator, Cytokinin signalin
Os05g0207300	UP	-	Similar to AMP-binding protein (Adenosine monophosphate binding protein 5 AMPBP5)
Os05g0215300	DOWN	OsGH9C1	Similar to CEL1=CELLULASE 1 (Fragment)
Os05g0222200	DOWN	-	Similar to H0103C06.6 protein
Os05g0226300	DOWN	OsDof17	Zinc finger, Dof-type domain containing protein
Os05g0226900	UP	OsPorA	Similar to Protochlorophyllide reductase (EC 1.3.1.33) (PCR) (NADPH- protochlorophyllide oxidoreductase) (POR) (Fragment)
Os05g0227600	UP	-	DNA mismatch repair protein MutS, core domain containing protein
Os05g0230700	DOWN	WSL5	Pentatricopeptide repeat protein, Chloroplast development under cold stres
Os05g0244700	UP	OsCAF1B	Component of the CCR4-NOTcomplex, Deadenylase, Deadenylation (poly(A) tail shortening), Development and stress respons
Os05g0245700	UP	-	Harpin-induced 1 domain containing protein
Os05g0247100	UP	OsUMA-MIT7	Protein of unknown function DUF6, transmembrane domain containing protein
Os05g0253200	DOWN	-	C2 domain containing protein
Os05g0256100	DOWN	-	Similar to cDNA clone:006-204-G09, full insert sequence
Os05g0256500	DOWN	-	Conserved hypothetical protein
Os05g0275000	DOWN	PGIP2	Leucine-rich repeat (LRR) protein, Inhibitor of fungal polygalacturonase, Defence respons
Os05g0275100	DOWN	PGIP1	Polygalacturonase inhibiting protein, Inhibitor of fungal polygalacturonase, Sheath blight resistanc
Os05g0304600	DOWN	OsUMA-MIT8	Protein of unknown function DUF6, transmembrane domain containing protein

Os05g0315100	DOWN	-	Hypothetical protein
Os05g0323400	UP	-	Protein of unknown function DUF26 domain containing protein
Os05g0323900	UP	-	Cupin, RmlC-type domain containing protein
Os05g0349700	DOWN	OsTET7	Tetraspanin domain containing protein
Os05g0361900	DOWN	-	Similar to Lateral organ boundaries domain 1
Os05g0362300	UP	OsGT43A	Glycosyl transferase, family 43 protein
Os05g0365300	DOWN	-	Conserved hypothetical protein
Os05g0368300	UP	OsPLC4	Similar to Phospholipase C (Fragment)
Os05g0369900	UP	OsCRR3	Serine/threonine protein kinase domain containing protein
Os05g0371166	UP	OsDjB5	Similar to DnaJ subfamily B member 13
Os05g0374500	DOWN	-	Similar to Leucoanthocyanidin dioxygenase-like protein
Os05g0377000	UP	-	Hypothetical conserved gene
Os05g0380850	DOWN	-	Ferroporti-1 domain containing protein
Os05g0381500	DOWN	OsLACS1	AMP-dependent synthetase and ligase domain containing protein
Os05g0388500	DOWN	-	Conserved hypothetical protein
Os05g0390100	DOWN	PRX65	Haem peroxidase, plant/fungal/bacterial family protein
Os05g0390550	DOWN	PRX71	Haem peroxidase family protein
Os05g0390800	DOWN	OsSAMS1	S-adenosylmethionine synthetase 1 (EC 2.5.1.6) (Methionine adenosyltransfer-ase 1) (AdoMet synthetase 1)
Os05g0397300	DOWN	OsA-BCB17	Similar to P-glycoprotein ABCB5
Os05g0400400	UP	Os-MYB2P-1	R2R3 MYB transcription factor, Regulation of phosphate-starvation responses and root architectur
Os05g0402700	UP	-	Conserved hypothetical protein
Os05g0405400	DOWN	-	Hypothetical conserved gene
Os05g0406100	UP	-	Conserved hypothetical protein
Os05g0406200	UP	OsDegp6	Similar to DEGP2 (DEGP PROTEASE 2); serine-type peptidase/ trypsin
Os05g0411200	DOWN	-	Conserved hypothetical protein
Os05g0414600	DOWN	RPL18	Similar to 60S ribosomal protein L18
Os05g0420600	UP	OsDjB6	Similar to Apobec-1 binding protein 2
Os05g0423701	DOWN	-	Similar to Dihydrolipoyl dehydrogenase
Os05g0426350	UP	-	Conserved hypothetical protein
Os05g0427100	DOWN	-	Serine/threonine protein kinase-related domain containing protein
Os05g0429400	DOWN	-	Hypothetical gene
Os05g0430400	DOWN	-	Conserved hypothetical protein
Os05g0431700	UP	-	DVL family protein
Os05g0432200	UP	OsAT7	Transferase family protein
Os05g0438800	UP	-	Conserved hypothetical protein
Os05g0442400	UP	-	Conserved hypothetical protein
Os05g0442700	DOWN	-	Phospholipase A2, active site domain containing protein
Os05g0445900	DOWN	-	Vegetative storage protein/acid phosphatase domain containing protein

Os05g0446550	DOWN	-	Similar to Stem 28 kDa glycoprotein
Os05g0449900	DOWN	-	Non-protein coding transcript
Os05g0452500	DOWN	-	Similar to Transcription factor MYBS2
Os05g0453300	DOWN	-	Conserved hypothetical protein
Os05g0464001	DOWN	MTd	Similar to Metallothionein-like protein 3B
Os05g0464300	UP	-	Conserved hypothetical protein
Os05g0469800	UP	OsRPL5	Similar to 60S ribosomal protein L11-2 (L16). Splice isoform 2
Os05g0471000	DOWN	OsGELP64	Lipase, GDSL domain containing protein
Os05g0471350	DOWN	-	UDP-glucuronosyl/UDP-glucosyltransferase family protein
Os05g0473500	DOWN	OsA-BCG12	ABC transporter-like domain containing protein
Os05g0474800	DOWN	-	Similar to FIP1
Os05g0477600	DOWN	OsRePRP1.1	Proline-rich glycoprotein, ABA-dependent inhibition of root growt
Os05g0482501	UP	OsRePRP1.2	Proline-rich glycoprotein, ABA-dependent inhibition of root growt
Os05g0488600	UP	OsIAA17	Similar to Auxin-responsive protein IAA17
Os05g0494000	UP	OsAT-IV	Aminotransferase, class IV family protein
Os05g0495600	DOWN	Os-miR1425	Hypothetical protein
Os05g0498900	DOWN	DIP3	Similar to Glycosyl hydrolases family 18
Os05g0506200	DOWN	-	Conserved hypothetical protein
Os05g0506500	UP	-	Similar to SHR5-receptor-like kinase (Fragment)
Os05g0506800	UP	-	Protein kinase, catalytic domain domain containing protein
Os05g0511700	DOWN	-	Pentatricopeptide repeat domain containing protein
Os05g0514600	UP	OsPPR920	Pentatricopeptide repeat domain containing protein
Os05g0514800	UP	OsLOX7	Similar to Lipoxygenase (Fragment)
Os05g0515600	UP	-	Pentatricopeptide repeat domain containing protein
Os05g0518600	UP	-	Hypothetical protein
Os05g0519600	DOWN	SODA1	Manganese superoxide dismutase, Heat-stress toleranc
Os05g0521700	DOWN	YGL1	Chl synthase, Chl biosynthesi
Os05g0526600	DOWN	UspA	UspA domain containing protein
Os05g0527000	UP	-	Mitochondrial carrier protein domain containing protein
Os05g0533800	UP	-	Protein of unknown function DUF538 family protein
Os05g0535700	UP	OsBBR1	Nucleotide-binding site leucine-rich repeat (NBS-LRR) protein, Bacterial blight resistanc
Os05g0535800	DOWN	-	Conserved hypothetical protein
Os05g0541301	UP	RPS4	Similar to 40S ribosomal protein S4, X isoform (Single copy abundant mRNA protein) (SCR10)
Os05g0549800	DOWN	OsEXO70FX1	Exo70 exocyst complex subunit domain containing protein

Os05g0555600	DOWN	-	Non-protein coding transcript
Os05g0555900	UP	OsBON3	Similar to predicted protein
Os05g0557400	DOWN	OsDJC31	Tetratricopeptide-like helical domain containing protein
Os05g0562000	DOWN	-	Similar to Acyl carrier protein
Os05g0563400	DOWN	-	Hypothetical protein
Os05g0563600	UP	-	Similar to predicted protein
Os05g0567300	DOWN	OsANN3	Similar to Annexin-like protein RJ4
Os05g0574400	UP	-	Similar to 50S ribosomal protein L1
Os05g0574800	DOWN	-	Dynamin family protein
Os05g0575300	DOWN	-	Conserved hypothetical protein
Os05g0575800	DOWN	OsVQ19	Similar to VQ motif family protein
Os05g0578500	UP	-	Conserved hypothetical protein
Os05g0582300	DOWN	-	Similar to Ubiquinol-cytochrome c reductase complex 8.0 kDa protein (EC 1.10.2.2)
Os05g0584400	UP	GSC	Similar to Fructose-bisphosphate aldolase, cytoplasmic isozyme (EC 4.1.2.13)
Os05g0586900	DOWN	-	Hypothetical conserved gene
Os05g0587300	DOWN	-	Inosine/uridine-preferring nucleoside hydrolase domain containing protein
Os05g0594200	DOWN	-	Non-protein coding transcript
Os05g0594500	DOWN	TCD5	Similar to electron carrier/ oxidoreductase
Os06g0103500	UP	-	Conserved hypothetical protein
Os06g0105400	UP	OsCc1	Cytochrome c
Os06g0106800	UP	-	Similar to Endoglucanase 3
Os06g0111300	DOWN	-	Hypothetical protein
Os06g0111500	UP	-	Octicosapeptide/Phox/Bem1p domain containing protein
Os06g0113900	DOWN	-	Phosphatidylinositol transfer protein-like, N-terminal domain containing protein
Os06g0115400	DOWN	-	GTP1/OBG domain containing protein
Os06g0116400	DOWN	-	Similar to peptide transporter PTR2-B
Os06g0116500	DOWN	-	Similar to Gibberellin-regulated protein 2 precursor
Os06g0126500	UP	-	Similar to Actin 1
Os06g0128600	DOWN	MID1	R-R-type MYB-like transcription factor, Response to drought stress during reproductive developmen
Os06g0129400	UP	ONAC012	No apical meristem (NAM) protein domain containing protein
Os06g0132400	DOWN	ROS1c	5-methylcytosine DNA glycosylase/lyase, Demethylation of retrotransposon Tos17, Promotion of Tos17 transposition, Seed developmen
Os06g0133200	UP	ROS1D	Similar to DNA glycosylase/lyase 701
Os06g0133900	UP	-	Homeodomain-like containing protein
Os06g0137600	DOWN	-	Conserved hypothetical protein
Os06g0138600	DOWN	-	Conserved hypothetical protein
Os06g0140700	UP	-	Conserved hypothetical protein
Os06g0146100	DOWN	-	Conserved hypothetical protein

Os06g0146600	UP	-	Pyruvate decarboxylase (EC 4.1.1.1) (Fragment)
Os06g0146800	UP	-	Similar to Ser-thr protein kinase (Fragment)
Os06g0146900	UP	OsGRL11	Similar to electron transporter
Os06g0149100	DOWN	OsZIP5	Similar to Metal transport protein
Os06g0150400	UP	OsEXO70 B3	Exo70 exocyst complex subunit family protein
Os06g0156600	UP	OsWRKY7 0	WRKY transcription factor 70
Os06g0163200	DOWN	OsEXP4	Alpha-expansin OsEXPA4
Os06g0164500	UP	-	Hypothetical conserved gene
Os06g0166500	DOWN	-	Similar to Phosphate starvation regulator protein (Regulatory protein of P-starvation acclimation response Psr1)
Os06g0167150	UP	OsC3'H1	p-Coumaroyl ester 3-hydroxylase, Determination of lignin content, composition and the degree of cell wall cross-linkin
Os06g0167200	UP	osACA7	Similar to autoinhibited calcium ATPase
Os06g0167400	UP	OsRLCK1 89	Serine/threonine protein kinase domain containing protein
Os06g0172000	DOWN	-	KOW domain containing protein
Os06g0175900	UP	OsGELP69	Conserved hypothetical protein
Os06g0177000	UP	OsGELP72	Hypothetical conserved gene
Os06g0180666	UP	-	Putative rRNA methylase family protein
Os06g0183700	DOWN	GA2OX4	GA 2-oxidase4, GA metabolis
Os06g0187950	DOWN	-	Hypothetical conserved gene
Os06g0191800	UP	ZRP4	Similar to O-methyltransferase ZRP4 (EC 2.1.1.-
Os06g0192800	DOWN	OsFTL10	Similar to ZCN12 protein
Os06g0194900	UP	OsTPS1	Similar to SL-TPS/P
Os06g0199100	DOWN	OsDJ-1D	Similar to 4-methyl-5(B-hydroxyethyl)-thiazol monophosphate biosynthesis enzyme
Os06g0200350	DOWN	-	Conserved hypothetical protein
Os06g0200700	DOWN	-	Zinc finger, RING/FYVE/PHD-type domain containing protein
Os06g0202900	DOWN	OsUGT70 6C3	UDP-glucuronosyl/UDP-glucosyltransferase family protein
Os06g0203000	DOWN	-	Similar to hydrogen-transporting ATP synthase, rotational mechanism
Os06g0205250	UP	-	Conserved hypothetical protein
Os06g0206900	UP	-	Similar to F14O23.10 protein
Os06g0207950	DOWN	-	Hypothetical gene
Os06g0208151	DOWN	OsMYB86-L2	Similar to Myb-related protein
Os06g0215600	UP	AP2/EREB P	-
Os06g0217700	DOWN	OsGLT2	Similar to Glutamate synthase [NADH], amyloplastic

Os06g0218900	DOWN	-	Non-protein coding transcript
Os06g0219400	DOWN	-	Membrane attack complex component/perforin/complement C9 family protein
Os06g0220000	UP	-	Conserved hypothetical protein
Os06g0220500	UP	OsARF15	Similar to Auxin response factor 5
Os06g0226000	UP	OsFLA6	FAS1 domain domain containing protein
Os06g0228500	UP	-	Protein of unknown function DUF561 family protein
Os06g0230000	UP	-	Similar to GTP-binding protein era
Os06g0233800	UP	-	Similar to 50S ribosomal protein L12, chloroplast precursor (CL12)
Os06g0239100	DOWN	-	Similar to Malate dehydrogenase
Os06g0241100	UP	OsPPR6	Hypothetical conserved gene
Os06g0243100	UP	-	Similar to Translation initiation factor IF-2
Os06g0244000	UP	-	Hypothetical conserved gene
Os06g0245800	DOWN	OsCCR23	NAD(P)-binding domain containing protein
Os06g0246200	DOWN	OsSPS2	Similar to prenyl transferase
Os06g0248500	DOWN	-	Conserved hypothetical protein
Os06g0253350	UP	-	Hypothetical conserved gene
Os06g0253600	DOWN	-	Molecular chaperone, heat shock protein, Hsp40, DnaJ domain containing protein
Os06g0255900	DOWN	OsCAX1b	Similar to Cation/proton exchanger 1a
Os06g0256500	UP	-	Invasin/intimin cell-adhesion domain containing protein
Os06g0266800	DOWN	ACX1	Similar to Acyl-coenzyme A oxidase 1, peroxisomal (EC 1.3.3.6) (AOX 1) (Long- chain acyl-CoA oxidase) (AtCX1)
Os06g0272700	DOWN	-	Similar to dihydrolipoamide S-acetyltransferase
Os06g0284500	DOWN	-	Similar to glycoside hydrolase, family 28
Os06g0289300	UP	-	Hypothetical conserved gene
Os06g0292400	DOWN	Os6PGDH1	Cytosolic 6-phosphogluconate dehydrogenase
Os06g0294600	UP	-	Major facilitator superfamily, general substrate transporter domain containing protein
Os06g0295900	DOWN	OsFSD1.1	Similar to Superoxide dismutase [Fe], chloroplast (EC 1.15.1.1) (Fragment)
Os06g0306300	DOWN	ZN	Nickel/cobalt transporter, high-affinity domain containing protein
Os06g0314501	DOWN	-	Pleckstrin homology-type domain containing protein
Os06g0316800	DOWN	OsBAG3	Ubiquitin domain containing protein
Os06g0326400	DOWN	-	Ankyrin repeat containing protein
Os06g0329900	DOWN	OsSPX-MFS3	Splicing variant of SPX-MFS protein
Os06g0334100	DOWN	ChlM	Similar to Magnesium-protoporphyrin O-methyltransferase (EC 2.1.1.11) (Magnesium- protoporphyrin IX methyltransferase)
Os06g0335950	DOWN	-	Conserved hypothetical protein
Os06g0346300	UP	EPSPS	5-enolpyruvylshikimate-3-phosphate synthas

Os06g0367100	DOWN	-	K homology-like, alpha/beta domain containing protein
Os06g0483500	DOWN	-	Protein of unknown function DUF248, methyltransferase putative domain containing protein
Os06g0494400	DOWN	Oshox2	Similar to Homeodomain leucine zipper protein (Fragment)
Os06g0498900	DOWN	Pi9(t)	NB-ARC domain containing protein
Os06g0505202	DOWN	-	Hypothetical protein
Os06g0505700	UP	-	Conserved hypothetical protein
Os06g0521900	DOWN	-	Hypothetical protein
Os06g0536800	DOWN	OsGELP75	Similar to mRNA, clone: RTFL01-05-E01
Os06g0538900	UP	-	Conserved hypothetical protein
Os06g0542300	DOWN	OsGELP77	Lipase, GDSL domain containing protein
Os06g0547400	UP	OsWRKY113	Similar to WRKY transcription factor 63
Os06g0551800	DOWN	OsPOP11	Esterase/lipase/thioesterase domain containing protein
Os06g0557700	DOWN	-	Conserved hypothetical protein
Os06g0558766	DOWN	OsIAA20	Similar to Auxin-responsive protein IAA20 (Indoleacetic acid-induced protein 20)
Os06g0563000	UP	-	Hypothetical gene
Os06g0564700	UP	-	Similar to RING-H2 finger protein ATL1R (RING-H2 finger protein ATL8)
Os06g0567900	UP	-	Di-trans-poly-cis-decaprenylcistransferase family protein
Os06g0569500	UP	-	Pentatricopeptide repeat domain containing protein
Os06g0569900	DOWN	-	Similar to Cytochrome b-c1 complex subunit 8
Os06g0577350	UP	-	Similar to Cytochrome b-c1 complex subunit 8
Os06g0579800	UP	-	Hypothetical gene
Os06g0581500	DOWN	-	Hypothetical conserved gene
Os06g0587900	DOWN	-	Hypothetical protein
Os06g0591400	UP	OsGLR4.8	Hypothetical conserved gene
Os06g0592200	UP	Os-CYP709C11	Cytochrome P450 family protein
Os06g0592400	UP	-	Similar to RING-H2 finger protein ATL1R (RING-H2 finger protein ATL8)
Os06g0593100	UP	SUS2	Sucrose synthase 2 (EC 2.4.1.13)
Os06g0605900	DOWN	-	Helix-turn-helix, AraC type, subdomain 2 domain containing protein
Os06g0606700	UP	-	Conserved hypothetical protein
Os06g0612900	DOWN	-	Hypothetical protein
Os06g0622000	DOWN	OsRLCK203	Serine/threonine protein kinase domain containing protein
Os06g0626600	UP	-	Hypothetical protein
Os06g0632200	UP	-	Conserved hypothetical protein
Os06g0634700	DOWN	-	Conserved hypothetical protein
Os06g0638700	DOWN	-	Hypothetical gene

Os06g0639500	DOWN	-	Similar to quinone oxidoreductase
Os06g0642500	DOWN	OsOPR5	Similar to Oxo-phytodienoic acid reductase
Os06g0644400	DOWN	-	Conserved hypothetical protein
Os06g0645901	DOWN	-	Similar to predicted protein
Os06g0648200	DOWN	-	GDP-fucose protein O-fucosyltransferase domain containing protein
Os06g0649000	DOWN	-	Similar to Phi-1 protein
Os06g0656300	DOWN	-	UDP-glucuronosyl/UDP-glucosyltransferase family protein
Os06g0662200	UP	-	Similar to Transposase (Fragment)
Os06g0664200	DOWN	OsATL12	Amino acid transporter, transmembrane domain containing protein
Os06g0671600	DOWN	-	Hypothetical conserved gene
Os06g0671700	DOWN	-	Conserved hypothetical protein
Os06g0675700	UP	-	Peptidase M10A and M12B, matrixin and adamalysin family protein
Os06g0676400	UP	-	Protein kinase, core domain containing protein
Os06g0697000	UP	-	Non-protein coding transcript
Os06g0697600	UP	-	Similar to anthranilic acid methyltransferase 3
Os06g0698600	DOWN	-	Similar to Alanyl-tRNA synthetase
Os06g0698674	UP	-	Similar to Formin-like protein 14
Os06g0698748	DOWN	OsPUB39	U box domain containing protein
Os06g0704800	DOWN	-	Hypothetical gene
Os06g0705400	UP	-	Similar to secretory protein-like
Os06g0705600	UP	OsEXO70 F5	Conserved hypothetical protein
Os06g0707400	DOWN	Pgi2(Pgib)	Similar to Glucose-6-phosphate isomerase
Os06g0710700	UP	OsGSR1	Similar to Gibberellin-stimulated protein
Os06g0715500	UP	-	Similar to OSIGBa0139J17.1 protein
Os06g0724800	UP	OsTBL64	Protein of unknown function DUF231, plant domain containing protein
Os06g0725000	UP	OsDof20	Zinc finger, Dof-type family protein
Os06g0725300	UP	-	Hypothetical conserved gene
Os06g0726100	UP	-	Embryogenesis transmembrane protein
Os06g0728700	UP	-	Cytochrome P450 family protein
Os06g0729500	DOWN	-	Hypothetical conserved gene
Os06g0729800	DOWN	PRX78	Haem peroxidase domain containing protein
Os06g0729900	DOWN	-	Conserved hypothetical protein
Os06g0731200	DOWN	-	Similar to Glycine-rich protein (Fragment)
Os06g0731300	DOWN	OsPFPA2	Phosphofructokinase domain containing protein
Os06g0731600	DOWN	-	SAM dependent carboxyl methyltransferase family protein
Os07g0100300	DOWN	-	Hypothetical gene
Os07g0103000	UP	OsXTH15	Concanavalin A-like lectin/glucanase domain containing protein
Os07g0103100	UP	-	Non-protein coding transcript
Os07g0107300	UP	-	Similar to ACX4 (ACYL-COA OXIDASE 4); acyl-CoA oxidase/ oxidoreductase
Os07g0114000	DOWN	-	Similar to Xylanase inhibitor protein I precursor

Os07g0117900	UP	-	Glycoside hydrolase, subgroup, catalytic core domain containing protein
Os07g0120700	DOWN	-	Similar to H0124B04.15 protein
Os07g0129200	UP	-	Multi antimicrobial extrusion protein MatE family protein
Os07g0132001	UP	-	GTP-binding protein, HSR1-related domain containing protein
Os07g0133100	DOWN	-	Hypothetical conserved gene
Os07g0137500	DOWN	OsAGP26	Similar to AGP16
Os07g0147700	DOWN	PRX82	Plant peroxidase domain containing protein
Os07g0147800	DOWN	-	Hypothetical protein
Os07g0152500	DOWN	-	Protein of unknown function DUF538 domain containing protein
Os07g0158300	DOWN	-	Heavy metal transport/detoxification protein domain containing protein
Os07g0158400	UP	prx86	Similar to Peroxidase P7 (EC 1.11.1.7) (TP7)
Os07g0159200	UP	-	Bulb-type lectin domain domain containing protein
Os07g0160600	UP	-	Protein kinase, catalytic domain domain containing protein
Os07g0162400	UP	-	Similar to predicted protein
Os07g0168000	DOWN	OsMIOX	Myo-inositol oxygenase, Drought stress toleranc
Os07g0176600	UP	-	Ndr family protein
Os07g0181100	DOWN	-	Similar to Cysteine synthase (EC 4.2.99.8)
Os07g0182100	UP	AO	L-ascorbate oxidase, Cold, salt, drought stress respons
Os07g0185000	UP	KO4	Ent-kaurene oxidase, Diterpenoid phytoalexin biosynthesi
Os07g0188233	DOWN	OsKO1	Similar to Ent-kaurene oxidase 1 (Fragment)
Os07g0188249	DOWN	-	Hypothetical protein
Os07g0195300	UP	-	Conserved hypothetical protein
Os07g0207400	UP	-	Hypothetical conserved gene
Os07g0208000	UP	-	Protein kinase, core domain containing protein
Os07g0213300	DOWN	-	Hypothetical conserved gene
Os07g0215200	UP	-	Conserved hypothetical protein
Os07g0218200	UP	-	Hypothetical protein
Os07g0229700	DOWN	OsALDH2B2	Similar to Cytosolic aldehyde dehydrogenase RF2C
Os07g0236700	UP	-	Similar to UDP-galactose/UDP-glucose transporter
Os07g0240300	UP	OsFbox316	Leucine-rich repeat, cysteine-containing subtype containing protein
Os07g0246200	UP	-	Tetratricopeptide-like helical domain containing protein
Os07g0263400	UP	-	Similar to predicted protein
Os07g0275100	DOWN	OsCW-ZF6	Zinc finger, CW-type domain containing protein
Os07g0282300	DOWN	-	Sulfotransferase family protein
Os07g0283125	UP	TS	Similar to tryptophan synthase-related
Os07g0287000	DOWN	-	Conserved hypothetical protein
Os07g0297400	UP	OsCYL2	Putative cyclase family protein
Os07g0416700	DOWN	-	Protein kinase, core domain containing protein

Os07g0418500	UP	-	Cytochrome P450 domain containing protein
Os07g0421932	DOWN	-	Non-protein coding transcript
Os07g0438500	DOWN	-	Similar to Transcription initiation factor TFIID subunit 1
Os07g0438700	UP	-	Conserved hypothetical protein
Os07g0445600	UP	WRKY28	PAMP (pathogen-associated molecular pattern)-responsive transrepressor, Defense respons
Os07g0448800	UP	-	Uncharacterised protein family UPF0497, trans-membrane plant subgroup domain containing protein
Os07g0449100	UP	OsbZIP52	Basic leucine zipper (bZIP) transcription factor, Negative regulator of cold and drought stress respons
Os07g0450000	UP	-	Inositol monophosphatase/Fructose-1,6-bisphosphatase domain containing protein
Os07g0452801	DOWN	Os-SAUR26	Beta tubulin, autoregulation binding site domain containing protein
Os07g0464200	DOWN	-	Similar to toprim domain-containing protein
Os07g0464600	DOWN	-	Similar to High pI alpha-glucosidase
Os07g0469200	DOWN	-	Conserved hypothetical protein
Os07g0471300	DOWN	-	Similar to 50S ribosomal protein L24, chloroplast precursor (CL24)
Os07g0471900	UP	OsXTH10	Similar to Xyloglucan endo-transglucosylase/hydrolase 1
Os07g0480300	UP	-	ATPase, AAA-type, core domain containing protein
Os07g0490400	DOWN	OsEXO70G2	Exo70 exocyst complex subunit family protein
Os07g0491500	DOWN	-	eIF4-gamma/eIF5/eIF2-epsilon domain containing protein
Os07g0492000	UP	-	Similar to cDNA clone:J023083M02, full insert sequence
Os07g0496250	UP	-	Tautomerase domain containing protein
Os07g0498400	DOWN	-	Conserved hypothetical protein
Os07g0510400	DOWN	OsHAC704	Probable histone acetyltransferase HAC-like 2
Os07g0511500	DOWN	OsLTP2.6	Hypothetical conserved gene
Os07g0515200	UP	-	Oligopeptide transporter domain containing protein
Os07g0518500	DOWN	-	Similar to NBS-LRR disease resistance protein homologue
Os07g0519600	UP	-	Conserved hypothetical protein
Os07g0523100	DOWN	-	Similar to Benzoyl coenzyme A: benzyl alcohol benzoyl transferase (Benzoyl-CoA:benzyl alcohol/phenylethanol benzoyltransferase)
Os07g0523150	DOWN	-	Similar to Translocon-associated protein alpha subunit precursor (TRAP-alpha) (Signal sequence receptor alpha subunit) (SSR-alpha)
Os07g0524900	UP	-	Similar to Chaperone protein dnaJ
Os07g0527000	DOWN	OsbHLH155	Basic helix-loop-helix dimerisation region bHLH domain containing protein
Os07g0528200	DOWN	OsStr9	Similar to Senescence-associated protein DIN1
Os07g0530400	DOWN	-	Barwin-related endoglucanase domain containing protein

Os07g0534000	DOWN	Cht3*	Similar to Seed chitinase-c
Os07g0534300	UP	-	Homeodomain-like containing protein
Os07g0534700	UP	-	Protein of unknown function DUF789 domain containing protein
Os07g0539300	UP	OsIPT10	Hypothetical gene
Os07g0545800	DOWN	-	Similar to LysM domain containing protein
Os07g0548300	DOWN	OsA-BCG16	Similar to ABC transporter-like protein
Os07g0550500	UP	-	Surface protein from Gram-positive cocci, anchor region domain containing protein
Os07g0558100	UP	-	ARID/BRIGHT DNA-binding domain domain containing protein
Os07g0560100	DOWN	SQD2.1	Conserved hypothetical protein
Os07g0565400	DOWN	-	Similar to GRF zinc finger family protein, expressed
Os07g0565600	DOWN	OsGLR3.6	Similar to Glutamate receptor 3.4 precursor (Ligand-gated ion channel 3.4) (AtGLR4). Splice isoform 2
Os07g0566150	DOWN	-	Plant disease resistance response protein family protein
Os07g0567700	DOWN	-	Conserved hypothetical protein
Os07g0572100	UP	OsPP2C61	Similar to Protein phosphatase 2C-like
Os07g0573200	DOWN	-	NB-ARC domain containing protein
Os07g0573700	DOWN	-	Conserved hypothetical protein
Os07g0573900	DOWN	PR1A	Pathogenesis-related 1a protein, Regulation of abiotic stress response
Os07g0577700	UP	-	Non-protein coding transcript
Os07g0578333	DOWN	-	Concanavalin A-like lectin/glucanase, subgroup domain containing protein
Os07g0586200	DOWN	-	Transposase, MuDR, plant domain containing protein
Os07g0587500	DOWN	PsbR	Similar to Photosystem II 10 kDa polypeptide, chloroplast precursor
Os07g0590600	DOWN	-	ATPase, BadF/BadG/BcrA/BcrD type domain containing protein
Os07g0593400	UP	-	Hypothetical conserved gene
Os07g0597400	UP	-	Similar to H0315A08.1 protein
Os07g0607400	UP	-	Similar to RNA binding protein
Os07g0614000	DOWN	-	Hypothetical conserved gene
Os07g0614700	UP	-	Conserved hypothetical protein
Os07g0616200	DOWN	OsFLA16	FAS1 domain domain containing protein
Os07g0617800	UP	OsCDAP1	Alpha/beta hydrolase fold-3 domain containing protein
Os07g0619100	DOWN	-	Similar to Polyribonucleotide phophorylase (Fragment)
Os07g0621600	UP	OsNek3	Serine/threonine protein kinase, Never-in-mitosis A (NIMA)-related kinase, Pollen germinatio
Os07g0622700	UP	SLAC	C4-dicarboxylate transporter/malic acid transport protein family protein
Os07g0625400	UP	OsTSA	Similar to Tryptophan synthase alpha chain
Os07g0626700	DOWN	OsWNK3	Similar to MAP kinase (Ser/Thr kinase)
Os07g0626800	DOWN	-	Conserved hypothetical protein
Os07g0627100	UP	-	Non-protein coding transcript

Os07g0628700	DOWN	OsFbox347	Hypothetical conserved gene
Os07g0631300	UP	-	Similar to RF12 protein (Fragment)
Os07g0631900	DOWN	-	Similar to 40S ribosomal protein S15A
Os07g0638400	UP	-	Pentatricopeptide repeat domain containing protein
Os07g0642200	UP	-	Conserved hypothetical protein
Os07g0642800	DOWN	TPS3	Similar to terpene synthase 7
Os07g0643400	UP	OsTFIIS	Similar to DNA-directed RNA polymerase I subunit 12 (EC 2.7.7.6) (Nuclear RNA polymerase I small specific subunit Rpa12) (Zinc ribbon domain containing protein 1)
Os07g0643700	UP	OsDof21	Zinc finger, Dof-type domain containing protein
Os07g0643800	UP	-	Similar to OSIGBa0153E02-OSIGBa0093I20.13 protein
Os07g0645000	UP	CRO1	Similar to Calreticulin (Fragment)
Os07g0646100	UP	-	Similar to oxidoreductase family protein
Os07g0650100	UP	-	Similar to predicted protein
Os07g0656900	UP	OsAAO3	Similar to Aldehyde oxidase 1 homolog (Fragment)
Os07g0664400	UP	-	Similar to lectin-like receptor kinase 7
Os07g0669200	DOWN	OsFbox367	Cyclin-like F-box domain containing protein
Os07g0669250	DOWN	-	Conserved hypothetical protein
Os07g0669700	DOWN	OsFAD2-4	Conserved hypothetical protein
Os07g0669800	DOWN	OsCYP709C9	Similar to Cytochrome P450
Os07g0673400	DOWN	OsRePRP2.2	Proline-rich glycoprotein, ABA-dependent inhibition of root growt
Os07g0674100	UP	-	Hypothetical gene
Os07g0676900	UP	OsCesA3	Similar to Cellulose synthase-7
Os07g0677500	DOWN	-	Octicosapeptide/Phox/Bem1p domain containing protein
Os07g0680400	DOWN	-	Hypothetical conserved gene
Os07g0680600	DOWN	-	Conserved hypothetical protein
Os07g0680800	DOWN	OsPIP2a	Aquaporin
Os07g0681400	DOWN	-	Similar to Light induced protein like
Os07g0686400	DOWN	-	Similar to 60S ribosomal protein L44
Os07g0689400	DOWN	-	Conserved hypothetical protein
Os07g0689950	DOWN	-	Similar to O-methyltransferase ZRP4
Os07g0690900	DOWN	OsABCB26	ABC transporter-like domain containing protein
Os07g0691800	UP	-	Similar to predicted protein
Os07g0694700	UP	OsAGO18	Similar to Protein argonaute 18
Os07g0695400	DOWN	OsbHLH077	Helix-loop-helix DNA-binding domain containing protein

Os08g0106300	UP	-	Hypothetical conserved gene
Os08g0110400	DOWN	OsFKBP20-2	Peptidyl-prolyl cis-trans isomerase, FKBP-type domain containing protein
Os08g0111300	DOWN	-	Hypothetical conserved gene
Os08g0112300	UP	OsNDPK1	Nucleoside diphosphate kinase, Cell elongation process in coleoptil
Os08g0113100	UP	-	Similar to Expansin-like B1
Os08g0117100	DOWN	-	Leucine-rich repeat, typical subtype domain containing protein
Os08g0137900	DOWN	-	UDP-glucuronosyl/UDP-glucosyltransferase family protein
Os08g0138100	DOWN	-	Hypothetical protein
Os08g0138700	UP	-	Zinc finger, RING/FYVE/PHD-type domain containing protein
Os08g0138900	UP	-	Conserved hypothetical protein
Os08g0153900	UP	-	Similar to taxane 10-beta-hydroxylase
Os08g0154300	DOWN	-	Similar to taxane 10-beta-hydroxylase
Os08g0155400	UP	-	Similar to 60S ribosomal protein L44
Os08g0158200	UP	-	Similar to 60S ribosomal protein L44
Os08g0162800	UP	-	Protein of unknown function DUF6, transmembrane domain containing protein
Os08g0173300	DOWN	-	Similar to nodulin-like protein
Os08g0175600	DOWN	-	Conserved hypothetical protein
Os08g0176200	DOWN	Os_F0782	Ribosomal protein S14, conserved site domain containing protein
Os08g0182400	UP	-	Similar to permeases of the major facilitator superfamily
Os08g0183900	DOWN	-	Hypothetical conserved gene
Os08g0185300	UP	-	Serine/threonine protein kinase-related domain containing protein
Os08g0185701	DOWN	-	Similar to hydrolase, hydrolyzing O-glycosyl compounds
Os08g0186200	DOWN	Gns13	Glycoside hydrolase, family 17 protein
Os08g0187800	UP	CIGR1	Similar to Chitin-inducible gibberellin-responsive protein
Os08g0189500	UP	-	Hypothetical conserved gene
Os08g0191100	UP	-	Hypothetical conserved gene
Os08g0191433	DOWN	OsMyb7	Similar to Myb-related transcription factor LBM1
Os08g0191900	DOWN	-	Conserved hypothetical protein
Os08g0201100	UP	-	Similar to SRF8 (STRUBBELIG-RECEPTOR FAMILY 8)
Os08g0202300	DOWN	OsCYP37	Similar to predicted protein
Os08g0203300	DOWN	-	Hypothetical conserved gene
Os08g0203400	DOWN	-	Similar to SCARECROW
Os08g0206700	DOWN	DAO	Similar to Amine oxidase like protein (EC 1.4.3.6) (Copper amine oxidase)
Os08g0215300	DOWN	-	Similar to F20D23.3 protein
Os08g0224300	DOWN	-	Similar to Adenylyl-sulfate kinase
Os08g0229601	DOWN	-	Nucleotide-sugar transporter family protein
Os08g0232700	DOWN	OsAGP27	Hypothetical conserved gene
Os08g0240200	DOWN	-	Succinyl-CoA synthetase-like domain containing protein
Os08g0240500	DOWN	-	Conserved hypothetical protein

Os08g0254500	DOWN	OsGELP92	Similar to Esterase precursor (EC 3.1.1.-) (Early nodule-specific protein homolog) (Latex allergen Hev b 13)
Os08g0265000	DOWN	-	Armadillo-like helical domain containing protein
Os08g0266400	DOWN	-	Tetratricopeptide-like helical domain containing protein
Os08g0269700	DOWN	-	Similar to golgi transport 1 protein B
Os08g0272000	UP	-	Conserved hypothetical protein
Os08g0275200	UP	OsPME20	Pectin lyase fold/virulence factor domain containing protein
Os08g0277200	UP	OsSTRL12	Six-bladed beta-propeller, TolB-like domain containing protein
Os08g0278900	UP	OsSPX6	SPX, N-terminal domain containing protein
Os08g0293100	DOWN	-	Similar to Beta-ketoacyl-ACP synthase
Os08g0296900	DOWN	OsAlaAT3	Similar to Alanine aminotransferase
Os08g0297800	UP	-	Protein of unknown function DUF827, plant family protein
Os08g0298700	UP	-	Non-protein coding transcript
Os08g0320400	UP	-	Alpha/beta hydrolase fold-1 domain containing protein
Os08g0323600	DOWN	OSK28	SKP1 component domain containing protein
Os08g0330900	UP	-	Similar to Peroxidase (EC 1.11.1.7)
Os08g0331000	UP	-	Amine oxidase domain containing protein
Os08g0332700	DOWN	-	MULE transposase, conserved domain domain containing protein
Os08g0334900	UP	-	Similar to Receptor protein kinase
Os08g0339200	UP	-	Similar to KI domain interacting kinase 1
Os08g0351300	DOWN	-	Conserved hypothetical protein
Os08g0359200	DOWN	-	Nucleotide-binding, alpha-beta plait domain containing protein
Os08g0360300	DOWN	-	HAD-superfamily hydrolase, subfamily IA, variant 3 domain containing protein
Os08g0371200	UP	OsMYB86	Hypothetical conserved gene
Os08g0375400	UP	Os1-Cys	Similar to 1-Cys peroxiredoxin
Os08g0378000	DOWN	OsGELP93	Lipase, GDSL domain containing protein
Os08g0386200	DOWN	-	Similar to plectin-related
Os08g0387050	UP	-	Alpha/beta hydrolase fold-3 domain containing protein
Os08g0389700	UP	-	Alpha/beta hydrolase fold-3 domain containing protein
Os08g0392500	DOWN	-	Similar to Dirigent protein
Os08g0398350	DOWN	-	Allergen V5/Tpx-1 related family protein
Os08g0400200	DOWN	OsPP2C65	Similar to predicted protein
Os08g0411000	DOWN	-	Armadillo-like helical domain containing protein
Os08g0413850	DOWN	OsSCP40	Similar to Serine carboxypepsidase (Fragment)
Os08g0423600	DOWN	-	Short-chain dehydrogenase/reductase SDR domain containing protein
Os08g0423850	DOWN	OsObgC1	Similar to GTP1/OBG family protein
Os08g0425500	UP	-	Similar to predicted protein
Os08g0425700	UP	OsHAK7	Potassium transporter 4 (AtPOT4) (AtKUP3) (AtKT4)
Os08g0429200	UP	OsCW-ZF7	Zinc finger, CW-type domain containing protein

Os08g0433300	DOWN	OsUsp1	Rossmann-like alpha/beta/alpha sandwich fold domain containing protein
Os08g0434100	UP	OsUXS6	Hypothetical conserved gene
Os08g0434632	UP	prx109	Similar to Peroxidase (EC 1.11.1.7)
Os08g0439600	DOWN	POX3006	Similar to Peroxidase precursor (EC 1.11.1.7)
Os08g0442400	DOWN	PRX115	Similar to Cationic peroxidase
Os08g0445700	DOWN	WRKY47	WRKY transcription factor, Drought toleranc
Os08g0450700	DOWN	-	Protein of unknown function DUF3133 domain containing protein
Os08g0457400	DOWN	-	Hypothetical protein
Os08g0461600	DOWN	OsCML24	Similar to Calcium-binding protein CAST
Os08g0465800	DOWN	ONAC015	Similar to NAM / CUC2-like protein
Os08g0474866	UP	-	Conserved hypothetical protein
Os08g0475400	UP	OsPOP17	Similar to predicted protein
Os08g0480000	UP	-	BRCT domain containing protein
Os08g0482600	DOWN	-	Glycosyl-phosphatidyl inositol-anchored, plant domain containing protein
Os08g0484100	DOWN	TBPOs-2	Similar to 26S proteasome subunit 4-like protein (26S proteasome subunit AtRPT2a)
Os08g0487900	DOWN	APX2	Ascorbate peroxidase, ROS homeostasis, Chloroplast protection, Carbohydrate metabolism, Plant architecture, Fertility maintenanc
Os08g0490100	DOWN	-	KIP1-like domain containing protein
Os08g0490900	DOWN	-	Similar to ZIP transporter
Os08g0491000	DOWN	-	Non-protein coding transcript
Os08g0499300	DOWN	-	Similar to Cytochrome P450
Os08g0502700	DOWN	-	Protein of unknown function DUF266, plant family protein
Os08g0505200	UP	-	Transferase family protein
Os08g0508000	UP	-	Transferase domain containing protein
Os08g0508100	DOWN	OsFKII	Similar to Fructokinase (Fragment)
Os08g0509200	UP	-	Nucleotide-binding, alpha-beta plait domain containing protein
Os08g0510300	DOWN	OsUCL25	Similar to Chemocyanin precursor (Basic blue protein) (Plantacyanin)
Os08g0512700	UP	OsUCL26	Similar to blue copper protein
Os08g0515600	DOWN	OsRLCK245	Serine/threonine protein kinase-related domain containing protein
Os08g0515800	DOWN	-	Conserved hypothetical protein
Os08g0517150	UP	-	Protein of unknown function DUF599 family protein
Os08g0519700	DOWN	-	Aldolase-type TIM barrel domain containing protein
Os08g0520550	DOWN	OsNRT1.1A	Similar to Nitrate/chlorate transporter
Os08g0521000	UP	ROMT9	Caffeic acid O-methyltransferase, Flavonoid 3'-O-methyltransferase, Melatonin biosynthesis, Tricin biosynthesi
Os08g0525700	DOWN	-	FAD linked oxidase, N-terminal domain containing protein
Os08g0526100	DOWN	CSLF6	MLG (mixed-linkage glucan) synthase, Biosynthesis of MLG (cell wall polysaccharide

Os08g0529100	UP	OsACBP1	Acyl-CoA-binding protein , Stress respons
Os08g0531000	UP	OsPRO-PEP3	Small peptides, Anti-herbivore defense response, Response to wounding and oral secretio
Os08g0541300	DOWN	-	Similar to PolI-like DNA polymerase
Os08g0544400	UP	ORK1	Serine/threonine protein kinase-related domain containing protein
Os08g0546100	UP	-	Conserved hypothetical protein
Os08g0547200	UP	OsCCR29	Similar to dihydroflavonol-4-reductase
Os08g0550500	UP	-	Conserved hypothetical protein
Os08g0553500	DOWN	-	Non-protein coding transcript
Os08g0553700	DOWN	-	Hypothetical conserved gene
Os08g0556300	DOWN	OsGPT1	Similar to Glucose-6-phosphate/phosphate-translocator precursor
Os08g0556900	UP	GLP8-6	Germin-like protein 8-6, Disease resistanc
Os09g0116900	DOWN	OsACO1	Similar to Iron-responsive element binding protein (Fragment)
Os09g0130800	DOWN	SSIIIA	Starch synthase, Starch biosynthesi
Os09g0132900	UP	-	Pentatricopeptide repeat domain containing protein
Os09g0135800	DOWN	-	Conserved hypothetical protein
Os09g0248100	UP	-	Hypothetical conserved gene
Os09g0262000	UP	-	Similar to SHR5-receptor-like kinase (Fragment)
Os09g0264400	UP	SHR5	Protein kinase, core domain containing protein
Os09g0268800	DOWN	-	DNA-binding, integrase-type domain containing protein
Os09g0272000	UP	-	Conserved hypothetical protein
Os09g0272900	DOWN	ARE1	Determination of the grain yield, Modulation of nitrogen utilizatio
Os09g0273950	DOWN	-	Hypothetical conserved gene
Os09g0275400	UP	OsEXO70 FX4	Exo70 exocyst complex subunit family protein
Os09g0276600	DOWN	OsXTH17	Xyloglucan endotransglycosylase/hydrolase protein 8 precursor (EC 2.4.1.207) (End-xyloglucan transferase
Os09g0279500	DOWN	-	Conserved hypothetical protein
Os09g0290900	DOWN	OsXTH4	Glycoside hydrolase, family 16 domain containing protein
Os09g0298100	UP	-	Similar to Preprotein translocase secY subunit, chloroplast precursor (CpSecY)
Os09g0305300	UP	-	Conserved hypothetical protein
Os09g0316000	UP	-	Hypothetical gene
Os09g0326900	UP	-	Leucine-rich repeat, N-terminal domain containing protein
Os09g0327575	DOWN	-	Conserved hypothetical protein
Os09g0328332	UP	-	Similar to GAG1At protein
Os09g0328800	UP	-	Protein kinase, core domain containing protein
Os09g0329200	UP	OsCCR	Similar to Cinnamoyl-CoA reductase (EC 1.2.1.44)
Os09g0330000	DOWN	-	MIR domain containing protein
Os09g0337500	DOWN	-	Conserved hypothetical protein
Os09g0341500	DOWN	-	Tetratricopeptide-like helical domain containing protein
Os09g0344800	UP	-	Sulfotransferase family protein

Os09g0351800	DOWN	-	Similar to male sterility protein 2
Os09g0354900	UP	-	Similar to Indole-3-glycerol phosphate synthase-like
Os09g0356000	DOWN	-	Hypothetical conserved gene
Os09g0356200	DOWN	Os_F0451	Conserved hypothetical protein
Os09g0369400	DOWN	-	Conserved hypothetical protein
Os09g0382400	DOWN	-	Conserved hypothetical protein
Os09g0386200	DOWN	-	Xyloglucan fucosyltransferase family protein
Os09g0387000	UP	-	Uncharacterized conserved protein UCP016210 domain containing protein
Os09g0394600	UP	-	Hypothetical conserved gene
Os09g0396900	UP	-	Conserved hypothetical protein
Os09g0400500	DOWN	-	Protein of unknown function DUF247, plant family protein
Os09g0410125	DOWN	OsbZIP65	Conserved hypothetical protein
Os09g0410400	DOWN	-	Non-protein coding transcript
Os09g0412700	UP	-	Hypothetical gene
Os09g0413300	DOWN	-	Calmodulin binding protein-like domain containing protein
Os09g0415700	UP	-	Carotenoid oxygenase domain containing protein
Os09g0418000	DOWN	-	Plant disease resistance response protein family protein
Os09g0421700	UP	-	Conserved hypothetical protein
Os09g0422500	UP	OsWRKY69	WRKY transcription factor 69
Os09g0425300	UP	-	Conserved hypothetical protein
Os09g0425900	DOWN	-	Conserved hypothetical protein
Os09g0426100	UP	-	Hypothetical conserved gene
Os09g0431600	DOWN	OsABCA5	Similar to ABC transporter family, cholesterol/phospholipid flippase
Os09g0432300	UP	OsSET30	Similar to SET domain protein
Os09g0433800	DOWN	-	Prephenate dehydratase domain containing protein
Os09g0447000	DOWN	-	Protein of unknown function DUF1262 domain containing protein
Os09g0449800	UP	-	Conserved hypothetical protein
Os09g0451000	DOWN	Osal-phaCA4	Similar to carbonic anhydrase
Os09g0451500	UP	-	Non-protein coding transcript
Os09g0456700	UP	-	Endonuclease/exonuclease/phosphatase domain containing protein
Os09g0459800	UP	OsANN7	Similar to Annexin-like protein
Os09g0461700	UP	-	Non-protein coding transcript
Os09g0463300	DOWN	-	Sialidase domain containing protein
Os09g0469300	UP	OsRNS3	Similar to S-like ribonuclease (RNase PD2) (Fragment)
Os09g0477900	DOWN	OsCslA11	Conserved hypothetical protein
Os09g0479900	UP	-	Protein of unknown function DUF868, plant family protein
Os09g0482800	UP	AP2/EREBP	-

Os09g0484200	UP	OsTPS8	Class II trehalose-phosphate-synthase (TPS) gene family member, Salt stress tolerance, Control of yield-related trait
Os09g0486375	DOWN	-	Chaperonin Cpn60/TCP-1 family protein
Os09g0486500	DOWN	OsRLCK255	Similar to Avr9/Cf-9 induced kinase 1
Os09g0491100	UP	-	Zinc finger, CCHC-type domain containing protein
Os09g0491740	UP	OsGAD1	Similar to Glutamate decarboxylase
Os09g0491772	UP	Osal-phaCA6	Similar to Nectarin III
Os09g0497000	UP	OsREM5.2	Remorin, C-terminal region domain containing protein
Os09g0501200	UP	-	Similar to gibberellin receptor GID1L2
Os09g0505300	DOWN	-	Alpha/beta hydrolase fold-3 domain containing protein
Os09g0508900	DOWN	-	Multi antimicrobial extrusion protein MatE family protein
Os09g0509200	UP	OsUCL29	Cupredoxin domain containing protein
Os09g0518200	UP	-	Similar to predicted protein
Os09g0520550	DOWN	-	Esterase/lipase/thioesterase domain containing protein
Os09g0520800	UP	OsDof24	Similar to PBF protein
Os09g0522200	DOWN	-	Similar to Histone H2B.2
Os09g0523200	DOWN	-	Hypothetical conserved gene
Os09g0526300	DOWN	-	Myb/SANT-like domain domain containing protein
Os09g0530500	DOWN	OsWRKY30	WRKY transcription factor 30
Os09g0539000	UP	-	Pyridoxal phosphate-dependent transferase, major region, subdomain 1 domain containing protein
Os09g0539800	UP	-	tRNA-binding arm domain containing protein
Os09g0541500	UP	CYP76M2	Cytochrome P450 family protein
Os09g0547500	DOWN	-	Conserved hypothetical protein
Os09g0555100	UP	Os8bglu27	Similar to Beta-primeverosidase (EC 3.2.1.149)
Os09g0555150	UP	Os8bglu28	Similar to Amygdalin hydrolase isoform AH I precursor (EC 3.2.1.117)
Os09g0555800	UP	OsHAK26	Potassium uptake protein, kup domain containing protein
Os09g0556000	UP	HMGR	Similar to 3-hydroxy-3-methylglutaryl coenzyme A reductase (EC 1.1.1.34) (Fragment)
Os09g0556500	DOWN	-	Non-protein coding transcript
Os09g0563250	UP	-	Mitochodrial transcription termination factor-related family protein
Os09g0563800	DOWN	-	Non-protein coding transcript
Os09g0564000	UP	-	Protein of unknown function DUF630 domain containing protein
Os09g0564800	DOWN	-	Similar to Auxin response factor 21
Os09g0565700	UP	-	Protein of unknown function DUF599 family protein
Os09g0566050	UP	-	Conserved hypothetical protein
Os09g0568400	UP	OsUGlcAE2	NAD(P)-binding domain containing protein

Os10g0105900	UP	-	Similar to POT family protein
Os10g0111400	UP	-	Similar to Proteasome subunit beta type 1 (EC 3.4.25.1) (20S proteasome alpha subunit F) (20S proteasome subunit beta-6)
Os10g0112850	DOWN	NPP1	Nucleotide pyrophosphatase/phosphodiesterase, Negative effect on plant growth and starch accumulatio
Os10g0113100	UP	-	Leucine-rich repeat, N-terminal domain containing protein
Os10g0116000	DOWN	OsA-BCG45	ABC-2 type transporter domain containing protein
Os10g0124150	DOWN	OsBAG2	Similar to protein binding protein
Os10g0130500	DOWN	-	RabGAP/TBC domain containing protein
Os10g0131100	DOWN	-	Similar to Glutathione s-transferase (Fragment)
Os10g0132800	DOWN	-	Protein of unknown function DUF241, plant family protein
Os10g0135650	DOWN	-	Protein of unknown function DUF241, plant family protein
Os10g0135833	DOWN	-	Glycolipid transfer protein domain domain containing protein
Os10g0137300	DOWN	CP	Similar to Cysteine proteinase (EC 3.4.22.-)
Os10g0137700	UP	-	Conserved hypothetical protein
Os10g0140700	UP	-	Similar to predicted protein
Os10g0141200	DOWN	OsGELP98	Lipase, GDSL domain containing protein
Os10g0159300	UP	-	Armadillo-like helical domain containing protein
Os10g0162100	UP	OsFLA11	FAS1 domain domain containing protein
Os10g0163300	DOWN	-	Myb/SANT-like domain domain containing protein
Os10g0168900	DOWN	OsCCR24	Similar to Cinnamoyl CoA reductase
Os10g0180800	DOWN	-	Cytochrome P450 family protein
Os10g0188275	DOWN	-	Conserved hypothetical protein
Os10g0189100	DOWN	-	Heavy metal transport/detoxification protein domain containing protein
Os10g0190500	UP	-	Disease resistance protein domain containing protein
Os10g0198600	DOWN	-	Conserved hypothetical protein
Os10g0198900	UP	-	Cytochrome P450 family protein
Os10g0206000	DOWN	-	Conserved hypothetical protein
Os10g0208500	DOWN	-	Similar to Plastid-specific 30S ribosomal protein 2, chloroplast precursor (PSRP- 2)
Os10g0315400	UP	-	Similar to H1005F08.16 protein
Os10g0320001	UP	OsPFPA4	Similar to predicted protein
Os10g0322300	DOWN	-	Protein of unknown function DUF247, plant family protein
Os10g0340800	DOWN	-	Targeting for Xklp2 family protein
Os10g0343951	UP	-	Similar to cupin, RmlC-type
Os10g0344000	UP	BIP107	Similar to Eukaryotic translation initiation factor 5 (eIF-5)
Os10g0348900	DOWN	-	Similar to Pi5-1
Os10g0352000	DOWN	-	Non-protein coding transcript
Os10g0361000	UP	-	Hypothetical conserved gene
Os10g0362700	UP	-	UDP-glucuronosyl/UDP-glucosyltransferase family protein

Os10g0370500	UP	-	UDP-glucuronosyl/UDP-glucosyltransferase family protein
Os10g0371100	UP	-	Conserved hypothetical protein
Os10g0376900	UP	-	F-box domain, cyclin-like domain containing protein
Os10g0389300	UP	-	Ankyrin repeat containing protein
Os10g0393800	UP	-	Protein of unknown function DUF81 family protein
Os10g0394100	DOWN	OsRLCK265	Malectin-like carbohydrate-binding domain domain containing protein
Os10g0395400	DOWN	-	Conserved hypothetical protein
Os10g0396666	DOWN	OsRLCK266	Similar to OsD305
Os10g0404900	DOWN	-	Malectin-like carbohydrate-binding domain domain containing protein
Os10g0412050	DOWN	OsTPP7	Similar to predicted protein
Os10g0418900	UP	-	Conserved hypothetical protein
Os10g0421800	DOWN	OsPUB18	Armadillo-like helical domain containing protein
Os10g0437600	DOWN	-	Conserved hypothetical protein
Os10g0442100	DOWN	-	Endonuclease/exonuclease/phosphatase domain containing protein
Os10g0442400	DOWN	OsVIT2	Protein of unknown function DUF125, transmembrane family protein
Os10g0442800	UP	-	Serine/threonine protein kinase domain containing protein
Os10g0456000	DOWN	-	Similar to membrane alanyl aminopeptidase
Os10g0460900	DOWN	-	Zinc finger, RING-type domain containing protein
Os10g0462800	DOWN	-	Conserved hypothetical protein
Os10g0465700	DOWN	OsPPR1	Pentatricopeptide repeat domain containing protein
Os10g0467900	DOWN	-	Protein of unknown function DUF248, methyltransferase putative family protein
Os10g0479500	DOWN	WRKY76	WRKY transcription factor, Transcriptional repressor, Pathogen defens
Os10g0482000	DOWN	OsCIPK16	Similar to CBL-interacting protein kinase 16
Os10g0482066	DOWN	-	Zinc finger, U1-type domain containing protein
Os10g0486100	DOWN	BC6	Cellulose synthase A catalytic subunit 9, Cell wall biosynthesis and plant growt
Os10g0489500	DOWN	-	Conserved hypothetical protein
Os10g0491450	DOWN	OsSAP	Similar to senescence-associated protein DH
Os10g0493600	DOWN	-	Similar to cDNA, clone: J075189B12, full insert sequence
Os10g0493850	DOWN	OsCBSPPR1	Cystathionine beta-synthase, core domain containing protein
Os10g0496900	DOWN	-	Hypothetical conserved gene
Os10g0497300	DOWN	-	Zinc finger, C2H2-type domain containing protein
Os10g0498100	UP	-	Similar to AAA-type ATPase family protein
Os10g0498300	DOWN	-	Similar to Senescence-associated protein SAG102
Os10g0503100	DOWN	Oshox11	Homeodomain-related domain containing protein
Os10g0506100	UP	-	Conserved hypothetical protein
Os10g0506800	DOWN	ACO1	ACC oxidase, Ethylene biosynthesi

Os10g0509000	DOWN	PDIL2	Similar to protein disulfide isomerase
Os10g0511800	DOWN	-	Conserved hypothetical protein
Os10g0512400	UP	-	Similar to ARP protein
Os10g0512500	DOWN	-	Alpha/beta hydrolase fold-3 domain containing protein
Os10g0520400	UP	-	Domain of unknown function DUF547 domain containing protein
Os10g0521000	UP	OsUCL31	Cupredoxin domain containing protein
Os10g0521500	UP	-	Non-protein coding transcript
Os10g0521900	DOWN	-	Hypothetical protein
Os10g0522700	UP	-	NusB/RsmB/TIM44 domain containing protein
Os10g0524300	UP	OsSub58	Similar to Subtilisin-like protease
Os10g0524700	UP	-	EF hand domain containing protein
Os10g0525800	DOWN	-	Hypothetical protein
Os10g0528200	UP	-	Conserved hypothetical protein
Os10g0528300	UP	SAP1	A20/AN1 zinc-finger protein, Response to multiple biotic stresses, Regulation of abiotic stress response
Os10g0528400	UP	Os9bglu30	Similar to Beta-primeverosidase (EC 3.2.1.149)
Os10g0532100	DOWN	OsPILS1	Auxin efflux carrier domain containing protein
Os10g0532300	UP	OsHsp58.7	Similar to Heat shock protein 70 (Hsc70-5)
Os10g0534801	UP	-	Adenine nucleotide translocator 1 domain containing protein
Os10g0536000	UP	rpL32_9.1	Similar to Ribosomal L32
Os10g0538200	DOWN	-	Similar to Br FatA1
Os10g0544600	UP	-	Similar to cDNA clone:001-044-E12, full insert sequence
Os10g0546600	DOWN	-	Similar to Pyruvate dehydrogenase E1 beta subunit isoform 3 (EC 1.2.4.1)
Os10g0548000	DOWN	-	NB-ARC domain containing protein
Os10g0548700	DOWN	OsSGT	UDP-glucuronosyl/UDP-glucosyltransferase family protein
Os10g0549500	DOWN	-	Similar to predicted protein
Os10g0550100	DOWN	-	Glycoside hydrolase, family 29 (alpha-L-fucosidase) protein
Os10g0553800	UP	OsDREB1A	DRE-binding protein 1A
Os10g0556100	DOWN	-	Hypothetical conserved gene
Os10g0558200	UP	Roc6(t)	Similar to Homeobox protein GLABRA2 (Homeobox-leucine zipper protein ATHB-10) (HD-ZIP protein ATHB-10)
Os10g0559700	DOWN	-	Hypothetical conserved gene
Os10g0560700	UP	-	Hypothetical conserved gene
Os10g0561300	DOWN	-	Similar to Acyl carrier protein III, chloroplast precursor (ACP III)
Os10g0569500	UP	-	Sedlin domain containing protein
Os10g0569600	UP	LOGL9	Lysine decarboxylase-like protein, Response to abiotic stresse
Os10g0571300	UP	-	Hypothetical conserved gene
Os10g0572500	DOWN	-	Zinc finger, RING/FYVE/PHD-type domain containing protein
Os10g0573800	UP	OsPILS7b	Similar to auxin Efflux Carrier family protein

Os10g0574750	DOWN	-	Sulfotransferase family protein
Os10g0578950	DOWN	-	Similar to AMP-binding protein (Adenosine monophosphate binding protein 6 AMPBP6)
Os11g0102200	DOWN	-	Signal peptidase 22 kDa subunit family protein
Os11g0103000	DOWN	-	Cysteinyl-tRNA synthetase, class Ia family protein
Os11g0104400	UP	-	Zinc finger, DHHC-type, palmitoyltransferase domain containing protein
Os11g0104800	UP	-	ATPase, AAA-type, core domain containing protein
Os11g0105750	DOWN	-	Peptidase C1A, papain family protein
Os11g0106200	DOWN	-	Conserved hypothetical protein
Os11g0113700	UP	EIP3	Prephenate dehydratase domain containing protein
Os11g0116300	UP	-	Similar to Prephenate dehydratase
Os11g0118500	DOWN	Ub-CEP52-2	Similar to 60S ribosomal protein L40 (CEP52)
Os11g0124500	UP	OsERF	-
Os11g0128300	UP	-	Hypothetical conserved gene
Os11g0131700	UP	OsWAK95	Similar to Protein kinase domain containing protein, expressed
Os11g0138400	UP	-	Non-protein coding transcript
Os11g0140700	DOWN	-	Aldo/keto reductase family protein
Os11g0144900	DOWN	-	Pentatricopeptide repeat domain containing protein
Os11g0146300	UP	-	Non-protein coding transcript
Os11g0147150	UP	-	Hypothetical protein
Os11g0153000	UP	-	Similar to NB-ARC domain containing protein
Os11g0153600	DOWN	-	Hypothetical protein
Os11g0153700	DOWN	-	Hypothetical conserved gene
Os11g0153800	UP	-	Conserved hypothetical protein
Os11g0154500	DOWN	-	Similar to BLN1-1
Os11g0155500	UP	-	Cyclin-like F-box domain containing protein
Os11g0156300	DOWN	-	Hypothetical conserved gene
Os11g0157000	UP	OsRLCK290	Similar to Protein kinase domain containing protein
Os11g0160400	UP	-	Conserved hypothetical protein
Os11g0161700	UP	-	Hypothetical conserved gene
Os11g0162200	UP	-	Hypothetical protein
Os11g0167500	DOWN	-	Conserved hypothetical protein
Os11g0179000	UP	OsUGT98B3	UDP-glucuronosyl/UDP-glucosyltransferase family protein
Os11g0180900	UP	WAK112	Wall-associated kinase, Negative regulation of rice blast resistanc
Os11g0195600	UP	-	Hypothetical protein
Os11g0199200	UP	OspPGM	Plastidic phosphoglucomutase, Starch synthesis in rice polle
Os11g0201299	DOWN	-	Protein of unknown function DUF594 family protein
Os11g0202600	UP	-	Intron-encoded nuclease 2 domain containing protein

Os11g0204600	DOWN	OsEnS-134	Drug/metabolite transporter domain containing protein
Os11g0204800	DOWN	-	Non-protein coding transcript
Os11g0206850	DOWN	-	Hypothetical protein
Os11g0210300	UP	SIP28	Similar to SKIP interacting protein 28 (Fragment)
Os11g0210500	UP	-	Hypothetical gene
Os11g0211700	UP	-	Conserved hypothetical protein
Os11g0212900	UP	-	Conserved hypothetical protein
Os11g0214400	UP	-	Hypothetical conserved gene
Os11g0215100	UP	-	Similar to transparent testa 12 protein
Os11g0216100	DOWN	-	Hypothetical protein
Os11g0220300	DOWN	-	Conserved hypothetical protein
Os11g0227600	DOWN	-	Lipoxygenase, LH2 domain containing protein
Os11g0227800	DOWN	-	Conserved hypothetical protein
Os11g0228201	UP	-	Conserved hypothetical protein
Os11g0229100	DOWN	-	Glycoside hydrolase, catalytic core domain containing protein
Os11g0229300	DOWN	OsERF	-
Os11g0229333	DOWN	OsbHLH045	Helix-loop-helix DNA-binding domain containing protein
Os11g0229400	DOWN	OsRCCR2	Similar to Red chlorophyll catabolite reductase (Fragment)
Os11g0236932	UP	OsGELP105	Lipase, GDSL domain containing protein
Os11g0242200	DOWN	-	Metallophosphoesterase domain containing protein
Os11g0245200	DOWN	OsGSTU34	Thioredoxin fold domain containing protein
Os11g0270201	UP	OsFbox552	Hypothetical conserved gene
Os11g0274700	DOWN	Oshox23	Similar to HAHB-5 (Fragment)
Os11g0286800	DOWN	-	Similar to aminophospholipid ATPase
Os11g0292050	DOWN	-	Hypothetical conserved gene
Os11g0303600	UP	SIP2	Similar to SIPL
Os11g0305300	DOWN	-	Pentatricopeptide repeat domain containing protein
Os11g0308800	DOWN	OsSSIIc	Similar to Starch synthase II, chloroplast precursor (EC 2.4.1.21) (SS II) (GBSSII) (Granule-bound starch synthase II)
Os11g0425300	DOWN	-	Similar to Phosphoglycerate kinase
Os11g0438700	DOWN	-	Similar to UDP-glucoronosyl and UDP-glucosyl transferase family protein, expressed
Os11g0439600	UP	OsRLCK301	Protein serine/threonine kinase (Protein kinase)
Os11g0441500	UP	GRP	Similar to Glycine-rich cell wall structural protein 2 precursor
Os11g0455500	UP	-	Hypothetical protein
Os11g0463700	DOWN	-	Similar to cDNA clone:J013158D14, full insert sequence

Os11g0475500	UP	WSL3	Component of the plastid-encoded plastid RNA polymerase (PEP), peripheral subunit of PEP complex, OsPAP1/OspTAC3, Early chloroplast developmen
Os11g0481150	DOWN	OsSTA246	Similar to Beta-amylase PCT-BMYI (EC 3.2.1.2)
Os11g0482400	DOWN	-	Protein kinase, catalytic domain domain containing protein
Os11g0484500	UP	LOGL10	Similar to carboxy-lyase
Os11g0484700	UP	-	Conserved hypothetical protein
Os11g0488400	UP	-	Hypothetical genes
Os11g0488900	UP	-	Cytochrome P450-like protein (CYP86B1)
Os11g0489250	UP	-	Similar to terpene synthase 2
Os11g0490300	UP	-	Conserved hypothetical protein
Os11g0490600	DOWN	alphaGa1	Alpha-galactosidase precursor (EC 3.2.1.22) (Melibiase) (Alpha-D- galactoside galactohydrolase)
Os11g0498600	UP	-	Hypothetical protein
Os11g0503900	UP	FGL	Similar to Protochlorophyllide reductase B, chloroplastic
Os11g0514500	UP	PPR794	Pentatricopeptide repeat domain containing protein
Os11g0515700	DOWN	-	Alpha/beta hydrolase fold-1 domain containing protein
Os11g0520500	DOWN	-	Similar to Epoxide hydrolase
Os11g0531800	DOWN	OsPLIM2c	Similar to Transcription factor L2
Os11g0533100	DOWN	-	Similar to 10-deacetylbaccatin III 10-O-acetyltransferase
Os11g0539700	DOWN	-	Heavy metal transport/detoxification protein domain containing protein
Os11g0540100	DOWN	OsSET39	SET domain containing protein
Os11g0544000	DOWN	-	Similar to MutS domain V family protein, expressed
Os11g0544800	DOWN	-	Similar to Plus-3 domain containing protein, expressed
Os11g0549650	UP	CYP84A5	Coniferaldehyde 5-hydroxylase, Lignin biosynthesi
Os11g0549665	UP	-	RNA-binding, CRM domain domain containing protein
Os11g0549667	UP	Os_F0771	Protein of unknown function DUF295 family protein
Os11g0550800	DOWN	OsTRE	Similar to TRE1 protein (Fragment)
Os11g0552500	UP	-	Esterase/lipase/thioesterase domain containing protein
Os11g0555300	DOWN	OsRhmbd 17	Similar to Membrane protein
Os11g0556400	DOWN	-	Conserved hypothetical protein
Os11g0558900	DOWN	OsEMSA1	LysM domain-containing protein, Embryo sac developmen
Os11g0562600	DOWN	-	Cytochrome P450 family protein
Os11g0565920	DOWN	OsGSTU3 1	Similar to Glutathione S-transferase GSTU31 (Fragment)
Os11g0568300	DOWN	OsGSTU1 2	Similar to Tau class GST protein 3
Os11g0569500	DOWN	GST	Similar to Glutathione S-transferase TSI-1 (EC 2.5.1.18) (Glutathione S- transferase 1)
Os11g0569800	UP	OsGSTU4	Tau class GST protein 4
Os11g0570000	UP	OsGSTU3	Glutathione S-transferase, C-terminal-like domain containing protein

Os11g0572200	DOWN	OsHDMA 704	Similar to Amine oxidase, flavin-containing
Os11g0574333	UP	-	Heavy metal transport/detoxification protein domain containing protein
Os11g0576900	UP	-	Hypothetical gene
Os11g0578700	DOWN	OsSce1	Similar to SUMO E2 conjugating enzyme SCE1
Os11g0582000	UP	-	Peptidase aspartic, catalytic domain containing protein
Os11g0586001	DOWN	CDPK	Similar to Calcium-dependent protein kinase 3
Os11g0586300	DOWN	-	Zinc finger, RING/FYVE/PHD-type domain containing protein
Os11g0588300	UP	CYP97C2	Similar to Chloroplast carotenoid epsilon-ring hydroxylase
Os11g0591800	UP	-	Hypothetical conserved gene
Os11g0592000	UP	RLCK306	Receptor-like cytoplasmic kinase, Various stress responses, Regulation of resistance to bacterial leaf streak (BLS
Os11g0592200	UP	-	Similar to Branched-chain-amino-acid aminotransferase
Os11g0593700	UP	Os-MED12_2	Conserved hypothetical protein
Os11g0596250	UP	-	Flavin-containing monooxygenase FMO family protein
Os11g0597700	UP	-	Protein of unknown function DUF566 family protein
Os11g0598000	DOWN	OsGGT	Similar to DNA chromosome 4, ESSA I CONTIG fragment NO. 6 (Glucosyl-transferase like protein)
Os11g0598800	DOWN	OsEXPB4	Similar to beta-expansin EXPB4
Os11g0602500	UP	-	2OG-Fe(II) oxygenase domain containing protein
Os11g0602600	UP	-	Similar to Agenet domain containing protein, expressed
Os11g0605100	DOWN	OsERF	-
Os11g0606400	DOWN	-	Similar to Monosaccharid transporter
Os11g0615900	DOWN	OsRALF-29	Conserved hypothetical protein
Os11g0625801	DOWN	-	Similar to RIR1b protein precursor
Os11g0626700	DOWN	OsRLCK308	Similar to Protein kinase-like protein
Os11g0634200	UP	-	Zinc finger, RING/FYVE/PHD-type domain containing protein
Os11g0635300	UP	-	Mitochondrial carnitine/acylcarnitine carrier-like protein (A BOUT DE SOUFFLE) (Carnitine/acylcarnitine translocase-like protein) (CAC-like protein)
Os11g0635500	UP	-	Hypothetical gene
Os11g0638000	DOWN	OsACS2	Similar to 4-coumarate--CoA ligase-like 2
Os11g0639300	UP	-	Similar to NPH1-1
Os11g0655000	DOWN	-	Pentatricopeptide repeat domain containing protein
Os11g0659700	UP	OsFAD3	Similar to W-3 fatty acid desaturase
Os11g0661101	UP	OsTBL10	Hypothetical conserved gene
Os11g0664400	DOWN	-	Quinonprotein alcohol dehydrogenase-like domain containing protein
Os11g0664500	UP	-	Similar to cDNA clone:J013071M22, full insert sequence
Os11g0664800	DOWN	-	BTB domain containing protein

Os11g0667700	DOWN	CIPK15	Serine/threonine protein kinase, Abiotic stresse
Os11g0668300	DOWN	CHI	Chalcone isomerase domain containing protein
Os11g0668650	DOWN	-	Hypothetical protein
Os11g0669100	DOWN	-	Similar to protein binding / signal transducer
Os11g0670700	DOWN	-	Protein of unknown function DUF607 family protein
Os11g0670800	DOWN	-	Similar to GDA1/CD39 family protein, expressed
Os11g0670900	UP	-	Similar to ZF-HD homeobox protein (Fragment)
Os11g0672400	DOWN	-	Zinc finger, Tim10/DDP-type domain containing protein
Os11g0672600	DOWN	-	Similar to Phosphoglycerate mutase family protein, expressed
Os11g0672700	DOWN	-	Similar to Saccharopine dehydrogenase family protein, expressed
Os11g0678200	DOWN	-	Conserved hypothetical protein
Os11g0681400	DOWN	OsUGT75E1	UDP-glucuronosyl/UDP-glucosyltransferase family protein
Os11g0683500	DOWN	-	Conserved hypothetical protein
Os11g0683600	DOWN	-	Hypothetical gene
Os11g0686100	UP	Os-CYP94C2	Cytochrome P450 family protein
Os11g0689800	UP	-	Conserved hypothetical protein
Os11g0691400	DOWN	-	Protein of unknown function DUF3049 domain containing protein
Os11g0691500	DOWN	YGL138(t)	Signal recognition particle 54 kDa (SRP54) protein, Chloroplast developmen
Os11g0691650	UP	-	Similar to Signal recognition particle 54 kDa protein, chloroplast precursor (SRP54) (54 chloroplast protein) (54CP) (FFC)
Os11g0693800	UP	RPS25	Similar to Ribosomal protein S25 (40S ribosomal 25S subunit)
Os11g0695800	DOWN	NAC17	Blast disease-responsive transcription factor, Disease resistanc
Os11g0702100	UP	OsCAT8	Similar to Amino acid permease family protein, expressed
Os11g0704500	UP	OsOFP18	Protein of unknown function DUF623, plant domain containing protein
Os11g0706100	DOWN	-	Putative 5-3 exonuclease domain containing protein
Os12g0101800	DOWN	-	Conserved hypothetical protein
Os12g0104400	UP	-	Conserved hypothetical protein
Os12g0113001	UP	-	Similar to Ubiquinol-cytochrome c reductase complex 7.8 kDa protein (EC 1.10.2.2) (Mitochondrial hinge protein) (CR7)
Os12g0114000	DOWN	OsSTA250	Similar to Serine esterase family protein, expressed
Os12g0115801	UP	-	Plant disease resistance response protein family protein
Os12g0117600	DOWN	-	Myb transcription factor domain containing protein
Os12g0117700	DOWN	OsAAP11C	Similar to Amino acid carrier (Fragment)
Os12g0124500	UP	ESP2	Protein disulfide isomerase-like enzyme, Starch synthesis, Maturation of proglutelin in endosper
Os12g0125500	UP	Os_F0390	Similar to F-box domain containing protein
Os12g0128600	UP	-	Similar to OSIGBa0126J24.2 protein
Os12g0130100	DOWN	-	Similar to Glucose-1-phosphate adenylyltransferase

Os12g0130200	DOWN	-	Similar to Wali7 protein (Fragment)
Os12g0136900	DOWN	-	Conserved hypothetical protein
Os12g0138050	DOWN	ADH1	Alcohol dehydrogenase 1
Os12g0141000	DOWN	Adh2*	Similar to Alcohol dehydrogenase
Os12g0147800	UP	-	Hypothetical conserved gene
Os12g0148700	DOWN	OsWD40-187	Serine/threonine protein kinase-related domain containing protein
Os12g0153200	UP	OsWD40-188	Similar to Protein kinase domain containing protein, expressed
Os12g0156100	DOWN	-	Plant disease resistance response protein family protein
Os12g0156866	UP	-	Plant disease resistance response protein family protein
Os12g0159600	UP	-	Similar to Heat shock protein binding protein
Os12g0163500	UP	-	Similar to NB-ARC domain containing protein, expressed
Os12g0168100	UP	-	Hypothetical protein
Os12g0169600	DOWN	-	NB-ARC domain containing protein
Os12g0170800	DOWN	-	Hypothetical gene
Os12g0171500	DOWN	-	Similar to B0616E02-H0507E05.11 protein
Os12g0171801	DOWN	-	Similar to NBS-LRR-like protein
Os12g0171900	DOWN	-	Hypothetical gene
Os12g0175400	UP	-	Similar to NBS-LRR protein (Fragment)
Os12g0178700	DOWN	-	Hypothetical conserved gene
Os12g0183300	UP	OsPP2C74	Protein phosphatase 2C-like domain containing protein
Os12g0188566	DOWN	-	Similar to leucine-rich repeat family protein / protein kinase family protein
Os12g0189300	UP	-	Conserved hypothetical protein
Os12g0209000	DOWN	OsRLCK322	Hypothetical conserved gene
Os12g0209200	UP	-	Homeodomain-like containing protein
Os12g0211600	UP	OsOSC10	Squalene cyclase domain containing protein
Os12g0215950	DOWN	-	Similar to PHD-finger family protein, expressed
Os12g0216500	UP	OMT	Similar to Herbicide safener binding protein
Os12g0218100	UP	-	Hypothetical gene
Os12g0218500	UP	-	Hypothetical conserved gene
Os12g0219700	UP	-	Similar to predicted protein
Os12g0221600	UP	-	Similar to alkaline alpha galactosidase 3
Os12g0228800	DOWN	-	Similar to Tropinone reductase-I (EC 1.1.1.206) (TR-I) (Tropine dehydrogenase)
Os12g0229100	UP	-	Similar to Nod factor binding lectin-nucleotide phosphohydrolase
Os12g0230133	UP	-	UDP-glucuronosyl/UDP-glucosyltransferase family protein
Os12g0233300	DOWN	-	Similar to Adenosylhomocysteinase
Os12g0239900	UP	-	Penicillin-binding protein, transpeptidase fold domain containing protein
Os12g0240900	UP	-	Conserved hypothetical protein

Os12g0242100	UP	-	NB-ARC domain containing protein
Os12g0247700	UP	-	Pentatricopeptide repeat domain containing protein
Os12g0254825	UP	Os6PGDH 2	Similar to 6-phosphogluconate dehydrogenase
Os12g0257600	UP	OsLTPd12	Bifunctional inhibitor/plant lipid transfer protein/seed storage domain containing protein
Os12g0260500	UP	-	Conserved hypothetical protein
Os12g0263000	UP	OsFbox60 0	Similar to MYND finger family protein, expressed
Os12g0263050	UP	-	Similar to Cytochrome P450 family protein, expressed
Os12g0263600	DOWN	-	Hypothetical conserved gene
Os12g0267800	DOWN	LA1	Regulation of shoot gravitropism and tiller angle, Regulation of polar auxin transpor
Os12g0267900	DOWN	-	Similar to HVA22 protein
Os12g0281966	DOWN	-	Sulfotransferase family protein
Os12g0289600	DOWN	-	Sorghum bicolor leucine-rich repeat-containing extracellular glycoprotein precursor
Os12g0289800	DOWN	-	Non-protein coding transcript
Os12g0291000	DOWN	-	Conserved hypothetical protein
Os12g0292900	DOWN	-	Hypothetical protein
Os12g0417300	DOWN	-	Similar to CarD-like transcriptional regulator family protein, expressed
Os12g0420300	UP	OsFbox60 8	F-box domain, cyclin-like domain containing protein
Os12g0428000	UP	-	Similar to Xaa-Pro aminopeptidase 2 (EC 3.4.11.9)
Os12g0428200	UP	-	Hypothetical conserved gene
Os12g0433200	DOWN	GATB	Glutamyl-tRNA(Gln) amidotransferase subunit B, Primary root developmen
Os12g0433700	UP	-	Mitochondrial matrix Mmp37 domain containing protein
Os12g0439201	UP	-	Hypothetical conserved gene
Os12g0441300	DOWN	-	Non-protein coding transcript
Os12g0441700	UP	-	Similar to plastid-specific ribosomal protein 6
Os12g0442800	UP	-	Conserved hypothetical protein
Os12g0458100	UP	-	Similar to Rp1-like protein
Os12g0459300	DOWN	OsRLCK3 34	Serine/threonine protein kinase-related domain containing protein
Os12g0467300	DOWN	-	Leucine-rich repeat, N-terminal domain containing protein
Os12g0468300	DOWN	-	Similar to Leucine Rich Repeat family protein
Os12g0470000	DOWN	-	Conserved hypothetical protein
Os12g0477400	UP	-	Leucine-rich repeat domain containing protein
Os12g0480100	DOWN	-	Cell division cycle-associated protein domain containing protein
Os12g0491800	UP	-	Similar to Receptor kinase-like protein
Os12g0507500	DOWN	-	Protein kinase, catalytic domain domain containing protein

Os12g0517800	DOWN	-	Similar to Receptor kinase-like protein
Os12g0520200	UP	OsEXO70 FX8	Exo70 exocyst complex subunit family protein
Os12g0538600	UP	-	Non-protein coding transcript
Os12g0538700	UP	-	Similar to HAT family dimerisation domain containing protein, expressed
Os12g0539700	DOWN	-	Similar to F-box domain containing protein, expressed
Os12g0539751	DOWN	-	Hypothetical conserved gene
Os12g0541500	DOWN	-	Putative protein phosphatase 2C 76
Os12g0547600	DOWN	-	Conserved hypothetical protein
Os12g0548401	UP	OsGSTT1	Similar to Glutathione transferase AtGST 10 (EC 2.5.1.18)
Os12g0548501	UP	OsPR4d	Barwin domain containing protein
Os12g0548700	UP	OsPR4c	Similar to Barwin
Os12g0549700	DOWN	OsPR4	Similar to Chitin-binding allergen Bra r 2 (Fragments)
Os12g0559100	DOWN	OsFbox62 0	Cyclin-like F-box domain containing protein
Os12g0561000	UP	-	Conserved hypothetical protein
Os12g0564400	DOWN	-	Similar to Resistant protein
Os12g0567800	UP	OsFbox62 4	Similar to F-box domain containing protein, expressed
Os12g0569300	UP	-	NB-ARC domain containing protein
Os12g0569500	UP	-	Tetratricopeptide-like helical domain containing protein
Os12g0570700	UP	-	Similar to 40S ribosomal protein S9
Os12g0576300	DOWN	-	Similar to 40S ribosomal protein S9
Os12g0581900	UP	-	Similar to NB-ARC domain containing protein
Os12g0583300	DOWN	-	NB-ARC domain containing protein
Os12g0586300	UP	-	Similar to NB-ARC domain containing protein, expressed
Os12g0598000	UP	-	Conserved hypothetical protein
Os12g0599625	DOWN	-	Non-protein coding transcript
Os12g0600400	UP	-	Hypothetical conserved gene
Os12g0608633	DOWN	-	Conserved hypothetical protein
Os12g0611100	DOWN	-	Similar to Cytochrome P450 family protein
Os12g0611900	UP	-	Cytochrome P450 family protein
Os12g0623900	UP	-	Similar to GTP-binding protein engA
Os12g0628100	UP	-	Protein of unknown function DUF594 family protein
Os12g0628600	UP	OsLAC23	Cupredoxin domain containing protein
Os12g0629700	UP	Os-TBT2	Transferase family protein
Os12g0630100	UP	-	Conserved hypothetical protein
Os12g0632401	UP	-	Similar to Pectinesterase family protein
Os12g0634500	UP	-	Conserved hypothetical protein
Os12g0634600	UP	-	Hypothetical conserved gene
Os12g0635700	UP	-	Hypothetical conserved gene

Os12g0637400	UP	-	Hypothetical conserved gene
Os12g0638300	UP	OsRLCK348	Similar to Protein kinase domain containing protein, expressed
